# Supplementary material for: Antipsychotics result in more weight gain in antipsychotic naive patients than in patients after antipsychotic switch and weight gain is irrespective of psychiatric diagnosis: A meta-analysis
Source: PLoS One. 2021 Feb 17;16(2):e0244944. doi: 10.1371/journal.pone.0244944 (PMC7888647; doi:10.1371/journal.pone.0244944)
Supplement: S2 File — (DOCX) [file pone.0244944.s002.docx]

Supporting information

S1

Search strategies

**Pubmed 30-Jun-2019**

((((((((((((((((((((((((((((("chlorpromazine"[MeSH Terms] OR "chlorpromazine"[All Fields]) OR ("haloperidol"[MeSH Terms] OR "haloperidol"[All Fields])) OR ("bromperidol"[Supplementary Concept] OR "bromperidol"[All Fields])) OR ("fluphenazine"[MeSH Terms] OR "fluphenazine"[All Fields])) OR ("perphenazine"[MeSH Terms] OR "perphenazine"[All Fields])) OR ("clopenthixol"[MeSH Terms] OR "clopenthixol"[All Fields])) OR ("flupenthixol"[MeSH Terms] OR "flupenthixol"[All Fields])) OR ("clopenthixol"[MeSH Terms] OR "clopenthixol"[All Fields] OR "zuclopenthixol"[All Fields])) OR ("pimozide"[MeSH Terms] OR "pimozide"[All Fields])) OR ("penfluridol"[MeSH Terms] OR "penfluridol"[All Fields])) OR ("droperidol"[MeSH Terms] OR "droperidol"[All Fields])) OR ("loxapine"[MeSH Terms] OR "loxapine"[All Fields])) OR ("sulpiride"[MeSH Terms] OR "sulpiride"[All Fields])) OR ("amisulpride"[MeSH Terms] OR "amisulpride"[All Fields])) OR ("Asenapine"[Supplementary Concept] OR "Asenapine"[All Fields] OR ("aripiprazole"[MeSH Terms] OR "aripiprazole"[All Fields])) OR ("clozapine"[MeSH Terms] OR "clozapine"[All Fields])) OR ("olanzapine"[MeSH Terms] OR "olanzapine"[All Fields])) OR ("risperidone"[MeSH Terms] OR "risperidone"[All Fields])) OR ("paliperidone palmitate"[MeSH Terms] OR ("paliperidone"[All Fields] AND "palmitate"[All Fields]) OR "paliperidone palmitate"[All Fields] OR "paliperidone"[All Fields])) OR ("quetiapine fumarate"[MeSH Terms] OR ("quetiapine"[All Fields] AND "fumarate"[All Fields]) OR "quetiapine fumarate"[All Fields] OR "quetiapine"[All Fields])) OR ("brexpiprazole"[Supplementary Concept] OR "brexpiprazole"[All Fields])) OR ("cariprazine"[Supplementary Concept] OR "cariprazine"[All Fields])) OR ("lurasidone hydrochloride"[MeSH Terms] OR ("lurasidone"[All Fields] AND "hydrochloride"[All Fields]) OR "lurasidone hydrochloride"[All Fields] OR "lurasidone"[All Fields])) OR ("sertindole"[Supplementary Concept] OR "sertindole"[All Fields])) OR ("ziprasidone"[Supplementary Concept] OR "ziprasidone"[All Fields])) OR ("zotepine"[Supplementary Concept] OR "zotepine"[All Fields])) OR ("blonanserin"[Supplementary Concept] OR "blonanserin"[All Fields])) AND ("body weight"[MeSH Terms] OR ("body"[All Fields] AND "weight"[All Fields]) OR "body weight"[All Fields])) NOT (((((((((((addition[All Fields] OR augmentation[All Fields]) OR adjunctive[All Fields]) OR ("topiramate"[MeSH Terms] OR "topiramate"[All Fields])) OR ("sibutramine"[Supplementary Concept] OR "sibutramine"[All Fields])) OR ("metformin"[MeSH Terms] OR "metformin"[All Fields])) OR ("modafinil"[MeSH Terms] OR "modafinil"[All Fields])) OR ("lithium"[MeSH Terms] OR "lithium"[All Fields])) OR ("carbamazepine"[MeSH Terms] OR "carbamazepine"[All Fields])) OR ("valproic acid"[MeSH Terms] OR ("valproic"[All Fields] AND "acid"[All Fields]) OR "valproic acid"[All Fields])) OR ("delirium"[MeSH Terms] OR "delirium"[All Fields])) OR ("nausea"[MeSH Terms] OR "nausea"[All Fields]))

| RRecent queries | | | | |
| --- | --- | --- | --- | --- |
| Search | Add to builder | Query | Items found | Time |
| [#7](https://www.ncbi.nlm.nih.gov/pubmed/advanced) | [Add](https://www.ncbi.nlm.nih.gov/pubmed/advanced) | Search **((((((((((((((((((((((((((((((chlorpromazine) OR haloperidol) OR bromperidol) OR fluphenazine) OR perphenazine) OR clopenthixol) OR flupenthixol) OR zuclopenthixol) OR pimozide) OR penfluridol) OR droperidol) OR loxapine) OR sulpiride) OR amisulpride) OR asenapine) OR aripiprazole) OR clozapine) OR olanzapine) OR risperidone) OR paliperidone) OR quetiapine) OR brexpiprazole) OR cariprazine) OR lurasidone) OR sertindole) OR ziprasidone) OR zotepine) OR blonanserin)) AND body weight) NOT ((((((((((((addition) OR augmentation) OR adjunctive) OR topiramate) OR sibutramine) OR metformin) OR modafinil) OR lithium) OR carbamazepine) OR valproic acid) OR delirium) OR nausea)** Filters: | [1594](https://www.ncbi.nlm.nih.gov/pubmed/?cmd=HistorySearch&querykey=7) | 10:34:35 |
| [#6](https://www.ncbi.nlm.nih.gov/pubmed/advanced) | [Add](https://www.ncbi.nlm.nih.gov/pubmed/advanced) | Search **((((((((((((((((((((((((((((((chlorpromazine) OR haloperidol) OR bromperidol) OR fluphenazine) OR perphenazine) OR clopenthixol) OR flupenthixol) OR zuclopenthixol) OR pimozide) OR penfluridol) OR droperidol) OR loxapine) OR sulpiride) OR amisulpride) OR asenapine) OR aripiprazole) OR clozapine) OR olanzapine) OR risperidone) OR paliperidone) OR quetiapine) OR brexpiprazole) OR cariprazine) OR lurasidone) OR sertindole) OR ziprasidone) OR zotepine) OR blonanserin)) AND body weight) NOT ((((((((((((addition) OR augmentation) OR adjunctive) OR topiramate) OR sibutramine) OR metformin) OR modafinil) OR lithium) OR carbamazepine) OR valproic acid) OR delirium) OR nausea)** | [2337](https://www.ncbi.nlm.nih.gov/pubmed/?cmd=HistorySearch&querykey=6) | 10:32:25 |
| [#5](https://www.ncbi.nlm.nih.gov/pubmed/advanced) | [Add](https://www.ncbi.nlm.nih.gov/pubmed/advanced) | Search **(((((((((((addition) OR augmentation) OR adjunctive) OR topiramate) OR sibutramine) OR metformin) OR modafinil) OR lithium) OR carbamazepine) OR valproic acid) OR delirium) OR nausea** | [1806782](https://www.ncbi.nlm.nih.gov/pubmed/?cmd=HistorySearch&querykey=5) | 10:31:34 |
| [#4](https://www.ncbi.nlm.nih.gov/pubmed/advanced) | [Add](https://www.ncbi.nlm.nih.gov/pubmed/advanced) | Search **delirium** | [16522](https://www.ncbi.nlm.nih.gov/pubmed/?cmd=HistorySearch&querykey=4) | 10:28:30 |
| [#3](https://www.ncbi.nlm.nih.gov/pubmed/advanced) | [Add](https://www.ncbi.nlm.nih.gov/pubmed/advanced) | Search **nausea** | [63920](https://www.ncbi.nlm.nih.gov/pubmed/?cmd=HistorySearch&querykey=3) | 10:28:05 |
| [#2](https://www.ncbi.nlm.nih.gov/pubmed/advanced) | [Add](https://www.ncbi.nlm.nih.gov/pubmed/advanced) | Search **body weight** | [611520](https://www.ncbi.nlm.nih.gov/pubmed/?cmd=HistorySearch&querykey=2) | 10:27:31 |
| [#1](https://www.ncbi.nlm.nih.gov/pubmed/advanced) | [Add](https://www.ncbi.nlm.nih.gov/pubmed/advanced) | Search **(((((((((((((((((((((((((((chlorpromazine) OR haloperidol) OR bromperidol) OR fluphenazine) OR perphenazine) OR clopenthixol) OR flupenthixol) OR zuclopenthixol) OR pimozide) OR penfluridol) OR droperidol) OR loxapine) OR sulpiride) OR amisulpride) OR asenapine) OR aripiprazole) OR clozapine) OR olanzapine) OR risperidone) OR paliperidone) OR quetiapine) OR brexpiprazole) OR cariprazine) OR lurasidone) OR sertindole) OR ziprasidone) OR zotepine) OR blonanserin** | [79721](https://www.ncbi.nlm.nih.gov/pubmed/?cmd=HistorySearch&querykey=1) | 10:26:39 |

**Embase 30-Jun-2019**

((((((((((((((((((((((((((((("chlorpromazine"[MeSH Terms] OR "chlorpromazine"[All Fields]) OR ("haloperidol"[MeSH Terms] OR "haloperidol"[All Fields])) OR ("bromperidol"[Supplementary Concept] OR "bromperidol"[All Fields])) OR ("fluphenazine"[MeSH Terms] OR "fluphenazine"[All Fields])) OR ("perphenazine"[MeSH Terms] OR "perphenazine"[All Fields])) OR ("clopenthixol"[MeSH Terms] OR "clopenthixol"[All Fields])) OR ("flupenthixol"[MeSH Terms] OR "flupenthixol"[All Fields])) OR ("clopenthixol"[MeSH Terms] OR "clopenthixol"[All Fields] OR "zuclopenthixol"[All Fields])) OR ("pimozide"[MeSH Terms] OR "pimozide"[All Fields])) OR ("penfluridol"[MeSH Terms] OR "penfluridol"[All Fields])) OR ("droperidol"[MeSH Terms] OR "droperidol"[All Fields])) OR ("loxapine"[MeSH Terms] OR "loxapine"[All Fields])) OR ("sulpiride"[MeSH Terms] OR "sulpiride"[All Fields])) OR ("amisulpride"[MeSH Terms] OR "amisulpride"[All Fields])) OR ("asenapine"[Supplementary Concept] OR "asenapine"[All Fields])) OR ("aripiprazole"[MeSH Terms] OR "aripiprazole"[All Fields])) OR ("clozapine"[MeSH Terms] OR "clozapine"[All Fields])) OR ("olanzapine"[MeSH Terms] OR "olanzapine"[All Fields])) OR ("risperidone"[MeSH Terms] OR "risperidone"[All Fields])) OR ("paliperidone palmitate"[MeSH Terms] OR ("paliperidone"[All Fields] AND "palmitate"[All Fields]) OR "paliperidone palmitate"[All Fields] OR "paliperidone"[All Fields])) OR ("quetiapine fumarate"[MeSH Terms] OR ("quetiapine"[All Fields] AND "fumarate"[All Fields]) OR "quetiapine fumarate"[All Fields] OR "quetiapine"[All Fields])) OR ("brexpiprazole"[Supplementary Concept] OR "brexpiprazole"[All Fields])) OR ("cariprazine"[Supplementary Concept] OR "cariprazine"[All Fields])) OR ("lurasidone hydrochloride"[MeSH Terms] OR ("lurasidone"[All Fields] AND "hydrochloride"[All Fields]) OR "lurasidone hydrochloride"[All Fields] OR "lurasidone"[All Fields])) OR ("sertindole"[Supplementary Concept] OR "sertindole"[All Fields])) OR ("ziprasidone"[Supplementary Concept] OR "ziprasidone"[All Fields])) OR ("zotepine"[Supplementary Concept] OR "zotepine"[All Fields])) OR ("blonanserin"[Supplementary Concept] OR "blonanserin"[All Fields])) AND ("body weight"[MeSH Terms] OR ("body"[All Fields] AND "weight"[All Fields]) OR "body weight"[All Fields])) NOT (((((((((((addition[All Fields] OR augmentation[All Fields]) OR adjunctive[All Fields]) OR ("topiramate"[MeSH Terms] OR "topiramate"[All Fields])) OR ("sibutramine"[Supplementary Concept] OR "sibutramine"[All Fields])) OR ("metformin"[MeSH Terms] OR "metformin"[All Fields])) OR ("modafinil"[MeSH Terms] OR "modafinil"[All Fields])) OR ("lithium"[MeSH Terms] OR "lithium"[All Fields])) OR ("carbamazepine"[MeSH Terms] OR "carbamazepine"[All Fields])) OR ("valproic acid"[MeSH Terms] OR ("valproic"[All Fields] AND "acid"[All Fields]) OR "valproic acid"[All Fields])) OR ("delirium"[MeSH Terms] OR "delirium"[All Fields])) OR ("nausea"[MeSH Terms] OR "nausea"[All Fields]))

S2. Reasons for exclusion after studying publications.

Non-human: animal as test/study object.

Review: meta-analyses, systematics reviews, comment, discussion, guidelines.

Method: Pooled data, post hoc analyses, subgroup analysis, extended study on subpopulation of per protocol data, naturalistic studies, retrospective chart review, cohort studies, cross-sectional data, case report

Age: <18 years: all studies that included study subject of 17 years or younger were excluded

Intervention focussed weight change: Addition medication: studies that focus on weight change by adding another medication to an antipsychotic drug. Weight intervention study with drugs of psychotherapy

Diagnosis: Eating disorders: Anorexia Nervosa, bulimia

Data problem: No data on weight change, Only data on BMI

Protocol description of a study

Single dose study

Brain changes

S3

Publications included in the study

1. Ader M, Garvey WT, Phillips LS, Nemeroff CB, Gharabawi G, Mahmoud R, et al. Ethnic heterogeneity in glucoregulatory function during treatment with atypical antipsychotics in patients with schizophrenia. J Psychiatr Res. 2008;42(13):1076-85.

2. Ak M, Sezlev D, Sutcigil L, Akarsu S, Ozgen F, Yanik T. The investigation of leptin and hypothalamic neuropeptides role in first attack psychotic male patients: olanzapine monotherapy. Psychoneuroendocrinology. 2013;38(3):341-7.

3. Alptekin K, Hafez J, Brook S, Akkaya C, Tzebelikos E, Ucok A, et al. Efficacy and tolerability of switching to ziprasidone from olanzapine, risperidone or haloperidol: an international, multicenter study. Int Clin Psychopharmacol. 2009;24(5):229-38.

4. Al-Thanoon ZA, Mahmood IH. Metabolic changes caused by first generation antipsychotic versus second generation antipsychotic in schizophrenic patients. Journal of Pharmacy Research. 2013:468-71.

5. Anand R, Berggren L, Deix C, Toth A, McDonell P. A 6-year open-label study of the efficacy and safety of olanzapine long-acting injection in patients with schizophrenia: A post hoc analysis based on the European label recommendation. Neuropsychiatric Disease and Treatment. 2015;11:1349-57.

6. Apiquian R, Ulloa E, Fresan A, Loyzaga C, Nicolini H, Kapur S. Amoxapine shows atypical antipsychotic effects in patients with schizophrenia: results from a prospective open-label study. Schizophr Res. 2003;59(1):35-9.

7. Arranz B, San L, Duenas RM, Centeno M, Ramirez N, Salavert J, et al. Lower weight gain with the orally disintegrating olanzapine than with standard tablets in first-episode never treated psychotic patients. Hum Psychopharmacol. 2007;22(1):11-5.

8. Atmaca M, Kuloglu M, Tezcan E, Ustundag B. Serum leptin and triglyceride levels in patients on treatment with atypical antipsychotics. J Clin Psychiatry. 2003;64(5):598-604.

9. Azorin JM, Strub N, Loft H. A double-blind, controlled study of sertindole versus risperidone in the treatment of moderate-to-severe schizophrenia. Int Clin Psychopharmacol. 2006;21(1):49-56.

10. Ballon JS, Pajvani UB, Mayer LE, Freyberg Z, Freyberg R, Contreras I, et al. Pathophysiology of drug induced weight and metabolic effects: findings from an RCT in healthy volunteers treated with olanzapine, iloperidone, or placebo. J Psychopharmacol. 2018;32(5):533-40.

11. Baptista T, Alastre T, Contreras Q, Martinez JL, Araujo de Baptista E, Paez X, et al. Effects of the antipsychotic drug sulpiride on reproductive hormones in healthy men: relationship with body weight regulation. Pharmacopsychiatry. 1997;30(6):250-5.

12. Baptista T, Alastre T, Contreras Q, Martinez JL, Araujo de Baptista E, Paez X, et al. Effects of the antipsychotic drug sulpiride on reproductive hormones in healthy men: relationship with body weight regulation. Pharmacopsychiatry. 1997;30(6):250-5.

13. Baptista T, Davila A, El Fakih Y, Uzcategui E, Rangel NN, Olivares Y, et al. Similar frequency of abnormal correlation between serum leptin levels and BMI before and after olanzapine treatment in schizophrenia. Int Clin Psychopharmacol. 2007;22(4):205-11.

14. Baptista T, Martinez M, Lacruz A, Arellano A, Mendoza S, Beaulieu S, et al. Insulin resistance index and counter-regulatory factors during olanzapine or risperidone administration in subjects with schizophrenia. Schizophr Res. 2007;89(1-3):350-2.

15. Barak Y, Shamir E, Zemishlani H, Mirecki I, Toren P, Weizman R. Olanzapine vs. haloperidol in the treatment of elderly chronic schizophrenia patients. Prog Neuropsychopharmacol Biol Psychiatry. 2002;26(6):1199-202.

16. Barak Y. No weight gain among elderly schizophrenia patients after 1 year of risperidone treatment. J Clin Psychiatry. 2002;63(2):117-9.

17. Barak Y, Aizenberg D. Effects of olanzapine on lipid abnormalities in elderly psychotic patients. Drugs Aging. 2003;20(12):893-6.

18. Barak Y, Shamir E, Mirecki I, Weizman R, Aizenberg D. Switching elderly chronic psychotic patients to olanzapine. Int J Neuropsychopharmacol. 2004;7(2):165-9.

19. Basson BR, Kinon BJ, Taylor CC, Szymanski KA, Gilmore JA, Tollefson GD. Factors influencing acute weight change in patients with schizophrenia treated with olanzapine, haloperidol, or risperidone. J Clin Psychiatry. 2001;62(4):231-8.

20. Baymiller SP, Ball P, McMahon RP, Buchanan RW. Weight and blood pressure change during clozapine treatment. Clin Neuropharmacol. 2002;25(4):202-6.

21. Beasly Jr CM, Sanger T, Satterlee W, Tollefson G, Tran P, Hamilton S. Olanzapine versus placebo: results of a double-blind, fixed-dose olanzapine trial. Psychopharmacoly. 1996;124:159 - 67.

22. Beasley CM, Jr., Sutton VK, Hamilton SH, Walker DJ, Dossenbach M, Taylor CC, et al. A double-blind, randomized, placebo-controlled trial of olanzapine in the prevention of psychotic relapse. J Clin Psychopharmacol. 2003;23(6):582-94.

23. Begic D, Bise S, Cemalovic O. Risperidone and olanzapine comparison for weight changes in a 24 months open extension study. European Neuropsychopharmacology 2010;20(August 2010):s463.

24. Bergmann F, Zacher A, Nass A, Urban R, Werner C, Spevakne-Gorocs T, et al. Psychosocial functioning in patients with schizophrenia treated with aripiprazole - an office-based real-world setting. Results from the German post-marketing surveillance study. Pharmacopsychiatry. 2009;42(3):101-8.

25. Berwaerts J, Melkote R, Nuamah I, Lim P. A randomized, placebo- and active-controlled study of paliperidone extended-release as maintenance treatment in patients with bipolar I disorder after an acute manic or mixed episode. J Affect Disord. 2012;138(3):247-58.

26. Berwaerts J, Xu H, Nuamah I, Lim P, Hough D. Evaluation of the efficacy and safety of paliperidone extended-release in the treatment of acute mania: a randomized, double-blind, dose-response study. J Affect Disord. 2012;136(1-2):e51-e60.

27. Birur B, Thirthalli J, Janakiramaiah N, Shelton RC, Gangadhar BN. Dimensions of schizophrenia and their time course of response to a second generation antipsychotic olanzapine-A clinical study. Asian J Psychiatr. 2016;24:17-22.

28. Bitter I, Dossenbach MR, Brook S, Feldman PD, Metcalfe S, Gagiano CA, et al. Olanzapine versus clozapine in treatment-resistant or treatment-intolerant schizophrenia. Prog Neuropsychopharmacol Biol Psychiatry. 2004;28(1):173-80.

29. Bitter I, Treuer T, Dilbaz N, Oyffe I, Ciorabai EM, Gonzalez SL, et al. Patients' preference for olanzapine orodispersible tablet compared with conventional oral tablet in a multinational, randomized, crossover study. World J Biol Psychiatry. 2010;11(7):894-903.

30. Bobes J, Rejas J, Garcia-Garcia M, Rico-Villademoros F, Garcia-Portilla MP, Fernandez I, et al. Weight gain in patients with schizophrenia treated with risperidone, olanzapine, quetiapine or haloperidol: results of the EIRE study. Schizophr Res. 2003;62(1-2):77-88.

31. Bobo WV, Epstein RA, Shelton RC. Olanzapine monotherapy for acute depression in patients with bipolar I or II disorder: results of an 8-week open label trial. Hum Psychopharmacol. 2010;25(1):30-6.

32. Bobo WV, Jayathilake K, Lee MA, Meltzer HY. Changes in weight and body mass index during treatment with melperone, clozapine and typical neuroleptics. Psychiatry Res. 2010;176(2-3):114-9.

33. Bobo WV, Epstein RA, Jr., Shelton RC. Effects of orally disintegrating vs regular olanzapine tablets on body weight, eating behavior, glycemic and lipid indices, and gastrointestinal hormones: a randomized, open comparison in outpatients with bipolar depression. Ann Clin Psychiatry. 2011;23(3):193-201.

34. Bobo WV, Bonaccorso S, Jayathilake K, Meltzer HY. Prediction of long-term metabolic effects of olanzapine and risperidone treatment from baseline body mass index in schizophrenia and bipolar disorder. Psychiatry Res. 2011;189(2):200-7.

35. Bogenschutz MP, George Nurnberg H. Olanzapine versus placebo in the treatment of borderline personality disorder. J Clin Psychiatry. 2004;65(1):104-9.

36. Bortnick B, El-Khalili N, Banov M, Adson D, Datto C, Raines S, et al. Efficacy and tolerability of extended release quetiapine fumarate (quetiapine XR) monotherapy in major depressive disorder: a placebo-controlled, randomized study. J Affect Disord. 2011;128(1-2):83-94.

37. Bozina N, Medved V, Kuzman MR, Sain I, Sertic J. Association study of olanzapine-induced weight gain and therapeutic response with SERT gene polymorphisms in female schizophrenic patients. J Psychopharmacol. 2007;21(7):728-34.

38. Breier A, Sutton VK, Feldman PD, Kadam DL, Ferchland I, Wright P, et al. Olanzapine in the treatment of dopamimetic-induced psychosis in patients with Parkinson's disease. Biol Psychiatry. 2002;52(5):438-45.

39. Briffa D, Meehan T. Weight changes during clozapine treatment. Aust N Z J Psychiatry. 1998;32(5):718-21.

40. Bromel T, Blum WF, Ziegler A, Schulz E, Bender M, Fleischhaker C, et al. Serum leptin levels increae after initiation of clozapine therapy. Molecular Psychiatry. 2998;3:76-80.

41. Brown ES, Chamberlain W, Dhanani N, Paranjpe P, Carmody TJ, Sargeant M. An open-label trial of olanzapine for corticosteroid-induced mood symptoms. J Affect Disord. 2004;83(2-3):277-81.

42. Buchanan RW, Ball MP, Weiner E, Kirkpatrick B, Gold JM, McMahon RP, et al. Olanzapine treatment of residual positive and negative symptoms. Am J Psychiatry. 2005;162(1):124-9.

43. Buchanan RW, Panagides J, Zhao J, Phiri P, den Hollander W, Ha X, et al. Asenapine versus olanzapine in people with persistent negative symptoms of schizophrenia. J Clin Psychopharmacol. 2012;32(1):36-45.

44. Buckley PF, Goldstein JM, Emsley RA. Efficacy and tolerability of quetiapine in poorly responsive, chronic schizophrenia. Schizophr Res. 2004;66(2-3):143-50.

45. Budman CL, Gayer A, Lesser M, Shi Q, Bruun RD. An open-label study of the treatment efficacy of olanzapine for Tourette's disorder. J Clin Psychiatry. 2001;62(4):290-4.

46. Bushe C, Sniadecki J, Bradley AJ, Poole Hoffmann V. Comparison of metabolic and prolactin variables from a six-month randomised trial of olanzapine and quetiapine in schizophrenia. J Psychopharmacol. 2010;24(7):1001-9.

47. Bustillo JR, Buchanan RW, Irish D, Breier A. Differential effect of clozapine on weight: a controlled study. Am J Psychiatry. 1996;153(6):817-9.

48. Butterfield MI, Becker ME, Connor KM, Sutherland S, Churchill LE, Davidson JR. Olanzapine in the treatment of post-traumatic stress disorder: a pilot study. Int Clin Psychopharmacol. 2001;16(4):197-203.

49. Calabrese JR, Keck PE, Jr., Macfadden W, Minkwitz M, Ketter TA, Weisler RH, et al. A randomized, double-blind, placebo-controlled trial of quetiapine in the treatment of bipolar I or II depression. Am J Psychiatry. 2005;162(7):1351-60.

50. Calabrese JR, Sanchez R, Jin N, Amatniek J, Cox K, Johnson B, et al. Efficacy and Safety of Aripiprazole Once-Monthly in the Maintenance Treatment of Bipolar I Disorder: A Double-Blind, Placebo-Controlled, 52-Week Randomized Withdrawal Study. J Clin Psychiatry. 2017;78(3):324-31.

51. Cantillon M, Prakash A, Alexander A, Ings R, Sweitzer D, Bhat L. Dopamine serotonin stabilizer RP5063: A randomized, double-blind, placebo-controlled multicenter trial of safety and efficacy in exacerbation of schizophrenia or schizoaffective disorder. Schizophr Res. 2017;189:126-33.

52. Canuso CM, Grinspan A, Kalali A, Damaraju CV, Merriman U, Alphs L, et al. Medication satisfaction in schizophrenia: a blinded-initiation study of paliperidone extended release in patients suboptimally responsive to risperidone. Int Clin Psychopharmacol. 2010;25(3):155-64.

53. Carey P, Suliman S, Ganesan K, Seedat S, Stein DJ. Olanzapine monotherapy in posttraumatic stress disorder: efficacy in a randomized, double-blind, placebo-controlled study. Hum Psychopharmacol. 2012;27(4):386-91.

54. Chan HY, Lin WW, Lin SK, Hwang TJ, Su TP, Chiang SC, et al. Efficacy and safety of aripiprazole in the acute treatment of schizophrenia in Chinese patients with risperidone as an active control: a randomized trial. J Clin Psychiatry. 2007;68(1):29-36.

55. Chan HY, Lin AS, Chen KP, Cheng JS, Chen YY, Tsai CJ. An open-label, randomized, controlled trial of zotepine and risperidone for acutely ill, hospitalized, schizophrenic patients with symptoms of agitation. J Clin Psychopharmacol. 2013;33(6):747-52.

56. Chawla B, Luxton-Andrew H. Long-term weight loss observed with olanzapine orally disintegrating tablets in overweight patients with chronic schizophrenia. A 1 year open-label, prospective trial. Hum Psychopharmacol. 2008;23(3):211-6.

57. Chen CH, Lin TY, Chen TT, Chen VC, Lin NC, Shao WC, et al. A prospective study of glucose homeostasis in quetiapine-treated schizophrenic patients by using the intravenous glucose tolerance test. Prog Neuropsychopharmacol Biol Psychiatry. 2011;35(4):965-9.

58. Chen JJ, Chan HY, Chen CH, Gau SS, Hwu HG. Risperidone and olanzapine versus another first generation antipsychotic in patients with schizophrenia inadequately responsive to first generation antipsychotics. Pharmacopsychiatry. 2012;45(2):64-71.

59. Chen Y, Bobo WV, Watts K, Jayathilake K, Tang T, Meltzer HY. Comparative effectiveness of switching antipsychotic drug treatment to aripiprazole or ziprasidone for improving metabolic profile and atherogenic dyslipidemia: a 12-month, prospective, open-label study. J Psychopharmacol. 2012;26(9):1201-10.

60. Chen CY, Tang TC, Chen TT, Bai YM, Tsai HH, Chen HL, et al. Efficacy, tolerability, and safety of oral paliperidone extended release in the treatment of schizophrenia: a 24-week, open-label, prospective switch study in different settings in Taiwan. Neuropsychiatr Dis Treat. 2018;14:725-32.

61. Chengappa KN, Parepally H, Brar JS, Mullen J, Shilling A, Goldstein JM. A random-assignment, double-blind, clinical trial of once- vs twice-daily administration of quetiapine fumarate in patients with schizophrenia or schizoaffective disorder: a pilot study. Can J Psychiatry. 2003;48(3):187-94.

62. Chiliza B, Asmal L, Oosthuizen P, van Niekerk E, Erasmus R, Kidd M, et al. Changes in body mass and metabolic profiles in patients with first-episode schizophrenia treated for 12 months with a first-generation antipsychotic. Eur Psychiatry. 2015;30(2):277-83.

63. Chiu NY, Yang YK, Chen PS, Chang CC, Lee IH, Lee JR. Olanzapine in Chinese treatment-resistant patients with schizophrenia: an open-label, prospective trial. Psychiatry Clin Neurosci. 2003;57(5):478-84.

64. Chiu CC, Chen KP, Liu HC, Lu ML. The early effect of olanzapine and risperidone on insulin secretion in atypical-naive schizophrenic patients. J Clin Psychopharmacol. 2006;26(5):504-7.

65. Chiu CC, Chen CH, Chen BY, Yu SH, Lu ML. The time-dependent change of insulin secretion in schizophrenic patients treated with olanzapine. Prog Neuropsychopharmacol Biol Psychiatry. 2010;34(6):866-70.

66. Chouinard G, Kopala L, Labelle A, Beauclair L, Johnson SV, Singh KI. Phase-IV multicentre clinical study of risperidone in the treatment of outpatients with schizophrenia. The RIS-CAN-3 Study Group. Can J Psychiatry. 1998;43(10):1018-25.

67. Christensen AF, Poulsen J, Nielsen CT, Bork B, Christensen A, Christensen M. Patients with schizophrenia treated with aripiprazole, a multicentre naturalistic study. Acta Psychiatr Scand. 2006;113(2):148-53.

68. Chrzanowski WK, Marcus RN, Torbeyns A, Nyilas M, McQuade RD. Effectiveness of long-term aripiprazole therapy in patients with acutely relapsing or chronic, stable schizophrenia: a 52-week, open-label comparison with olanzapine. Psychopharmacology (Berl). 2006;189(2):259-66.

69. Chue P. Study of long-term quetiapine treatment The journal of applied research. 2005;5(2):246 - 52.

70. Chue P, Eerdekens M, Augustyns I, Lachaux B, Molcan P, Eriksson L, et al. Comparative efficacy and safety of long-acting risperidone and risperidone oral tablets. Eur Neuropsychopharmacol. 2005;15(1):111-7.

71. Citrome L, Stauffer VL, Chen L, Kinon BJ, Kurtz DL, Jacobson JG, et al. Olanzapine plasma concentrations after treatment with 10, 20, and 40 mg/d in patients with schizophrenia: an analysis of correlations with efficacy, weight gain, and prolactin concentration. J Clin Psychopharmacol. 2009;29(3):278-83.

72. Citrome L, Ota A, Nagamizu K, Perry P, Weiller E, Baker RA. The effect of brexpiprazole (OPC-34712) and aripiprazole in adult patients with acute schizophrenia: results from a randomized, exploratory study. Int Clin Psychopharmacol. 2016;31(4):192-201.

73. Ciudad A, Olivares JM, Bousono M, Gomez JC, Alvarez E. Improvement in social functioning in outpatients with schizophrenia with prominent negative symptoms treated with olanzapine or risperidone in a 1 year randomized, open-label trial. Prog Neuropsychopharmacol Biol Psychiatry. 2006;30(8):1515-22.

74. Claus A, Bollen J, De Cuyper H, Eneman M, Malfroid M, Peuskens J, et al. Risperidone versus haloperidol in the treatment of chronic schizophrenic inpatients: a multicentre double-blind comparative study. Acta Psychiatr Scand. 1992;85(4):295-305.

75. Cohen SA, Fitzgerald BJ, Khan SR, Khan A. The effect of a switch to ziprasidone in an adult population with autistic disorder: chart review of naturalistic, open-label treatment. J Clin Psychiatry. 2004;65(1):110-3.

76. Conley RR, Mahmoud R. A randomized double-blind study of risperidone and olanzapine in the treatment of schizophrenia or schizoaffective disorder. Am J Psychiatry. 2001;158(5):765-74.

77. Coppola D, Liu Y, Gopal S, Remmerie B, Samtani MN, Hough DW, et al. A one-year prospective study of the safety, tolerability and pharmacokinetics of the highest available dose of paliperidone palmitate in patients with schizophrenia. BMC Psychiatry. 2012;12:26.

78. Covell NH, Weissman EM, Essock SM. Weight gain with clozapine compared to first generation antipsychotic medications. Schizophr Bull. 2004;30(2):229-40.

79. Cutler AJ, Durgam S, Wang Y, Migliore R, Lu K, Laszlovszky I, et al. Evaluation of the long-term safety and tolerability of cariprazine in patients with schizophrenia: results from a 1-year open-label study. CNS Spectr. 2018;23(1):39-50.

80. Czobor P, Volavka J, Sheitman B, Lindenmayer JP, Citrome L, McEvoy J, et al. Antipsychotic-induced weight gain and therapeutic response: a differential association. J Clin Psychopharmacol. 2002;22(3):244-51.

81. Daurignac E, Leonard KE, BDubovsky SL. Increased lean body mass as an early indicator of olanzapine-induced weight gain in healthy men. International Clinical Psychopharmacology. 2015;30:23 - 8.

82. Davidson M, Harvey PD, Vervarcke J, Gagiano CA, De Hooge JD, Bray G, et al. A long-term, multicenter, open-label study of risperidone in elderly patients with psychosis. On behalf of the Risperidone Working Group. Int J Geriatr Psychiatry. 2000;15(6):506-14.

83. Davidson M, Emsley R, Kramer M, Ford L, Pan G, Lim P, et al. Efficacy, safety and early response of paliperidone extended-release tablets (paliperidone ER): results of a 6-week, randomized, placebo-controlled study. Schizophr Res. 2007;93(1-3):117-30.

84. De Deyn PP, Carrasco MM, Deberdt W, Jeandel C, Hay DP, Feldman PD, et al. Olanzapine versus placebo in the treatment of psychosis with or without associated behavioral disturbances in patients with Alzheimer's disease. Int J Geriatr Psychiatry. 2004;19(2):115-26.

85. de Haan L, van Amelsvoort T, Rosien K, Linszen D. Weight loss after switching from conventional olanzapine tablets to orally disintegrating olanzapine tablets. Psychopharmacology (Berl). 2004;175(3):389-90.

86. De Hert M, Schreurs V, Sweers K, Van Eyck D, Hanssens L, Sinko S, et al. Typical and atypical antipsychotics differentially affect long-term incidence rates of the metabolic syndrome in first-episode patients with schizophrenia: a retrospective chart review. Schizophr Res. 2008;101(1-3):295-303.

87. de Jesus Mari J, Lima MS, Costa AN, Alexandrino N, Rodrigues-Filho S, de Oliveira IR, et al. The prevalence of tardive dyskinesia after a nine month naturalistic randomized trial comparing olanzapine with conventional treatment for schizophrenia and related disorders. Eur Arch Psychiatry Clin Neurosci. 2004;254(6):356-61.

88. Deberdt WG, Dysken MW, Rappaport SA, Feldman PD, Young CA, Hay DP, et al. Comparison of olanzapine and risperidone in the treatment of psychosis and associated behavioral disturbances in patients with dementia. Am J Geriatr Psychiatry. 2005;13(8):722-30.

89. Deepak TS, Raveesh RS, Parashivamurty BM, Kumar MS, Majgi SM, Nagesh HN. Clinical assessment of weight gain with atypical antipsychotics - blonanserine vs amisulpride. Journal of Clinical and Diagnotic Research. 2015;9(6):7-10.

90. Dossenbach MR, Kratky P, Schneidman M, Grundy SL, Metcalfe S, Tollefson GD, et al. Evidence for the effectiveness of olanzapine among patients nonresponsive and/or intolerant to risperidone. J Clin Psychiatry. 2001;62 Suppl 2:28-34.

91. Dossenbach M, Treuer T, Kryzhanovskaya L, Saylan M, Dominguez S, Huang X. Olanzapine versus chlorpromazine in the treatment of schizophrenia: a pooled analysis of four 6-week, randomized, open-label studies in the Middle East and North Africa. Journal of Clinical Psychopharmacology. 2007;27(4):329-37.

92. Durgam S, Earley W, Lipschitz A, Guo H, Laszlovszky I, Nemeth G, et al. An 8-Week Randomized, Double-Blind, Placebo-Controlled Evaluation of the Safety and Efficacy of Cariprazine in Patients With Bipolar I Depression. Am J Psychiatry. 2016;173(3):271-81.

93. Durgam S, Greenberg WM, Li D, Lu K, Laszlovszky I, Nemeth G, et al. Safety and tolerability of cariprazine in the long-term treatment of schizophrenia: results from a 48-week, single-arm, open-label extension study. Psychopharmacology (Berl). 2017;234(2):199-209.

94. Ebenbichler CF, Laimer M, Eder U, Mangweth B, Weiss E, Hofer A, et al. Olanzapine induces insulin resistance: results from a prospective study. J Clin Psychiatry. 2003;64(12):1436-9.

95. Emsley R, Turner HJ, Schronen J, Botha K, Smit R, Oosthuizen PP. A single-blind, randomized trial comparing quetiapine and haloperidol in the treatment of tardive dyskinesia. J Clin Psychiatry. 2004;65(5):696-701.

96. Emsley R, Medori R, Koen L, Oosthuizen PP, Niehaus DJ, Rabinowitz J. Long-acting injectable risperidone in the treatment of subjects with recent-onset psychosis: a preliminary study. J Clin Psychopharmacol. 2008;28(2):210-3.

97. Faries DE, Ascher-Svanum H, Nyhuis AW, Kinon BJ. Switching from risperidone to olanzapine in a one-year, randomized, open-label effectiveness study of schizophrenia. Curr Med Res Opin. 2008;24(5):1399-405.

98. Fleischhacker WW, McQuade RD, Marcus RN, Archibald D, Swanink R, Carson WH. A double-blind, randomized comparative study of aripiprazole and olanzapine in patients with schizophrenia. Biol Psychiatry. 2009;65(6):510-7.

99. Fleischhacker WW, Gopal S, Lane R, Gassmann-Mayer C, Lim P, Hough D, et al. A randomized trial of paliperidone palmitate and risperidone long-acting injectable in schizophrenia. Int J Neuropsychopharmacol. 2012;15(1):107-18.

100. Fleischhacker WW, Sanchez R, Perry PP, Jin N, Peters-Strickland T, Johnson BR, et al. Aripiprazole once-monthly for treatment of schizophrenia: double-blind, randomised, non-inferiority study. Br J Psychiatry. 2014;205(2):135-44.

101. Forsthoff A, Grunze H, Seemuller F, Stampfer R, Dittmann S, Amann B, et al. Risperidone monotherapy in manic inpatients: an open label, multicentre trial. World J Biol Psychiatry. 2007;8(4):256-61.

102. Frankenburg FR, Zanarini MC, Kando J, Centorrino F. Clozapine and body mass change. Biol Psychiatry. 1998;43(7):520-4.

103. Fu DJ, Bossie CA, Sliwa JK, Ma YW, Alphs L. Paliperidone palmitate versus oral risperidone and risperidone long-acting injection in patients with recently diagnosed schizophrenia: a tolerability and efficacy comparison. Int Clin Psychopharmacol. 2014;29(1):45-55.

104. Fu DJ, Turkoz I, Simonson RB, Walling DP, Schooler NR, Lindenmayer JP, et al. Paliperidone palmitate once-monthly reduces risk of relapse of psychotic, depressive, and manic symptoms and maintains functioning in a double-blind, randomized study of schizoaffective disorder. J Clin Psychiatry. 2015;76(3):253-62.

105. Gaebel W, Schreiner A, Bergmans P, de Arce R, Rouillon F, Cordes J, et al. Relapse prevention in schizophrenia and schizoaffective disorder with risperidone long-acting injectable vs quetiapine: results of a long-term, open-label, randomized clinical trial. Neuropsychopharmacology. 2010;35(12):2367-77.

106. Gaebel W, Schreiner A, Bergmans P, de Arce R, Rouillon F, Cordes J, et al. Relapse prevention in schizophrenia and schizoaffective disorder with ripseridone long-acting injectable vs quetiapine: Results of a long-term open label randomized clinical trial. Neuropsychopharmacology. 2011;25:2367 - 77.

107. Ganguli R, Brar JS, Ayrton Z. Weight gain over 4 months in schizophrenia patients: a comparison of olanzapine and risperidone. Schizophr Res. 2001;49(3):261-7.

108. Garriga M, Mallorqui A, Serrano L, Rios J, Salamero M, Parellada E, et al. Food craving and consumption evolution in patients starting treatment with clozapine. Psychopharmacology (Berl). 2019;236(11):3317-27.

109. Garyfallos G, Dimelis D, Kouniakis P, Sidiropoulos N, Karastergiou A, Lavrentiadis G, et al. Olanzapine versus risperidone: weight gain and elevation of serum triglyceride levels. Eur Psychiatry. 2003;18(6):320-1.

110. Gastpar M, Masiak M, Latif MA, Frazzingaro S, Medori R, Lombertie ER. Sustained improvement of clinical outcome with risperidone long-acting injectable in psychotic patients previously treated with olanzapine. J Psychopharmacol. 2005;19(5 Suppl):32-8.

111. Gharabawi GM, Gearhart NC, Lasser RA, Mahmoud RA, Zhu Y, Mannaert E, et al. Maintenance therapy with once-monthly administration of long-acting injectable risperidone in patients with schizophrenia or schizoaffective disorder: a pilot study of an extended dosing interval. Ann Gen Psychiatry. 2007;6:3.

112. Godleski LS, Goldsmith LJ, Vieweg WV, Zettwoch NC, Stikovac DM, Lewis SJ. Switching from depot antipsychotic drugs to olanzapine in patients with chronic schizophrenia. J Clin Psychiatry. 2003;64(2):119-22.

113. Gomez-Esteban JC, Zarranz JJ, Velasco F, Lezcano E, Lachen MC, Rouco I, et al. Use of ziprasidone in parkinsonian patients with psychosis. Clin Neuropharmacol. 2005;28(3):111-4.

114. Gopal S, Vijapurkar U, Lim P, Morozova M, Eerdekens M, Hough D. A 52-week open-label study of the safety and tolerability of paliperidone palmitate in patients with schizophrenia. J Psychopharmacol. 2011;25(5):685-97.

115. Gorobets LN. Contribution of Leptin to the Formation of Neuroleptic Obesity in Patients with Schizophrenia during Antipsychotic Therapy. Experimental Methods for Clinical Practice. 2008;146(3):348-50.

116. Grootens KP, van Veelen NM, Peuskens J, Sabbe BG, Thys E, Buitelaar JK, et al. Ziprasidone vs olanzapine in recent-onset schizophrenia and schizoaffective disorder: results of an 8-week double-blind randomized controlled trial. Schizophr Bull. 2011;37(2):352-61.

117. Guille C, Sachs GS, Ghaemi SN. A naturalistic comparison of clozapine, risperidone, and olanzapine in the treatment of bipolar disorder. J Clin Psychiatry. 2000;61(9):638-42.

118. Guo X, Fang M, Zhai J, Wang B, Wang C, Hu B, et al. Effectiveness of maintenance treatments with atypical and typical antipsychotics in stable schizophrenia with early stage: 1-year naturalistic study. Psychopharmacology (Berl). 2011;216(4):475-84.

119. Gupta S, Masand PS, Virk S, Schwartz T, Hameed A, Frank BL, et al. Weight decline in patients switching from olanzapine to quetiapine. Schizophr Res. 2004;70(1):57-62.

120. Gutierrez Fraile M. SWITCHING TO ZIPRASIDONE IN THE CLINICAL

PRACTICE SETTING: AN OPEN-LABEL STUDY. International Journal of Psychiatry in Medicine. 2013;45(2):125 - 42.

121. Haessler F, Glaser T, Pap AF, Diefenbacher A, Rets O. A double-blind placebo-controlled discontinuation study of zuclopenthixol for the theratment of agressive and disruptive behaviours in adults with mental retardation: Seondary parameter analyses. Pharmacopsychiatry. 2008;41:232 - 9.

122. Hale AS, Azorin JM, Lemming OM, Maehlum E. Sertindole in the long-term treatment of schizophrenia. Int Clin Psychopharmacol. 2012;27(4):231-7.

123. Han C, Lee MS, Pae CU, Ko YH, Patkar AA, Jung IK. Usefulness of long-acting injectable risperidone during 12-month maintenance therapy of bipolar disorder. Prog Neuropsychopharmacol Biol Psychiatry. 2007;31(6):1219-23.

124. Hardy TA, Henry RR, Forrester TD, Kryzhanovskaya LA, Campbell GM, Marks DM, et al. Impact of olanzapine or risperidone treatment on insulin sensitivity in schizophrenia or schizoaffective disorder. Diabetes Obes Metab. 2011;13(8):726-35.

125. Hargarter L, Cherubin P, Bergmans P, Keim S, Rancans E, Bez Y, et al. Intramuscular long-acting paliperidone palmitate in acute patients with schizophrenia unsuccessfully treated with oral antipsychotics. Prog Neuropsychopharmacol Biol Psychiatry. 2015;58:1-7.

126. Harris E, Eth E. Weight gian during neuroleptic treatment. International Journal of Nursing Students. 1981;18(3):171-5.

127. Hashimoto N, Toyomaki A, Honda M, Miyano S, Nitta N, Sawayama H, et al. Long-term efficacy and tolerability of quetiapine in patients with schizophrenia who switched from other antipsychotics because of inadequate therapeutic response-a prospective open-label study. Ann Gen Psychiatry. 2015;14(1):1.

128. Hatta K, Sato K, Hamakawa H, Takebayashi H, Kimura N, Ochi S, et al. Effectiveness of second-generation antipsychotics with acute-phase schizophrenia. Schizophr Res. 2009;113(1):49-55.

129. Henderson DC, Cagliero E, Gray C, Nasrallah RA, Hayden DL, Schoenfeld DA, et al. Clozapine, diabetes mellitus, weight gain, and lipid abnormalities: A five-year naturalistic study. Am J Psychiatry. 2000;157(6):975-81.

130. Herran A, Garcia-Unzueta MT, Amado JA, de La Maza MT, Alvarez C, Vazquez-Barquero JL. Effects of long-term treatment with antipsychotics on serum leptin levels. Br J Psychiatry. 2001;179:59-62.

131. Herrera-Estrella M, Apiquian R, Fresan A, Sanchez-Torres I. The effects of amisulpride on five dimensions of psychopathology in patients with schizophrenia: a prospective open-label study. BMC Psychiatry. 2005;5:22.

132. Hirsch SR, Kissling W, Bauml J, Power A, O'Connor R. A 28-week comparison of ziprasidone and haloperidol in outpatients with stable schizophrenia. J Clin Psychiatry. 2002;63(6):516-23.

133. Hollifield M, Thompson PM, Ruiz JE, Uhlenhuth EH. Potential effectiveness and safety of olanzapine in refractory panic disorder. Depress Anxiety. 2005;21(1):33-40.

134. Honer WG, MacEwan GW, Gendron A, Stip E, Labelle A, Williams R, et al. A randomized, double-blind, placebo-controlled study of the safety and tolerability of high-dose quetiapine in patients with persistent symptoms of schizophrenia or schizoaffective disorder. J Clin Psychiatry. 2012;73(1):13-20.

135. Hong CJ, Lin CH, Yu YW, Yang KH, Tsai SJ. Genetic variants of the serotonin system and weight change during clozapine treatment. Pharmacogenetics. 2001;11(3):265-8.

136. Hori H, Ueda N, Yoshimura R, Yamamoto H, Wani K, Etoh Y, et al. Olanzapine orally disintegrating tablets (Zyprexa Zydis) rapidly improve excitement components in the acute phase of first-episode schizophrenic patients: an open-label prospective study. World J Biol Psychiatry. 2009;10(4 Pt 3):741-5.

137. Hosojima H, Togo T, Odawara T, Hasegawa K, Miura S, Kato Y, et al. Early effects of olanzapine on serum levels of ghrelin, adiponectin and leptin in patients with schizophrenia. J Psychopharmacol. 2006;20(1):75-9.

138. Hsieh MH, Lin WW, Chen ST, Chen KC, Chen KP, Chiu NY, et al. A 64-week, multicenter, open-label study of aripiprazole effectiveness in the management of patients with schizophrenia or schizoaffective disorder in a general psychiatric outpatient setting. Ann Gen Psychiatry. 2010;9:35.

139. Hu S, Yao M, Peterson BS, Xu D, Hu J, Tang J, et al. A randomized, 12-week study of the effects of extendedrelease paliperidone (paliperidone ER) and olanzapine on metabolic profile, weight, insulin resistance, and β-cell function in schizophrenic patients. Psychopharmacoly. 2013;230:3 - 13.

140. Huang M, Yu L, Pan F, Lu S, Hu S, Hu J, et al. A randomized, 13-week study assessing the efficacy and metabolic effects of paliperidone palmitate injection and olanzapine in first-episode schizophrenia patients. Prog Neuropsychopharmacol Biol Psychiatry. 2018;81:122-30.

141. Hummer M, Kemmler G, Kurz M, Kurzthaler I, Oberbauer H, Fleischhacker WW. Weight gain induced by clozapine. Eur Neuropsychopharmacol. 1995;5(4):437-40.

142. Hwang JP, Yang CH, Lee TW, Tsai SJ. The efficacy and safety of olanzapine for the treatment of geriatric psychosis. J Clin Psychopharmacol. 2003;23(2):113-8.

143. Hwang TJ, Lee SM, Sun HJ, Lin HN, Tsai SJ, Lee YC, et al. Amisulpride versus risperidone in the treatment of schizophrenic patients: a double-blind pilot study in Taiwan. J Formos Med Assoc. 2003;102(1):30-6.

144. Ingole S, Belorkar NR, Waradkar P, Shrivastava M. Comparison of effects of olanzapine and risperidone on body mass index and blood sugar level in schizophrenic patients. Indian J Physiol Pharmacol. 2009;53(1):47-54.

145. Ishigooka J, Inada T, Miura S. Olanzapine versus haloperidol in the treatment of patients with chronic schizophrenia: results of the Japan multicenter, double-blind olanzapine trial. Psychiatry Clin Neurosci. 2001;55(4):403-14.

146. Ishigooka J, Nakamura J, Fujii Y, Iwata N, Kishimoto T, Iyo M, et al. Efficacy and safety of aripiprazole once-monthly in Asian patients with schizophrenia: a multicenter, randomized, double-blind, non-inferiority study versus oral aripiprazole. Schizophr Res. 2015;161(2-3):421-8.

147. Ishigooka J, Iwashita S, Tadori Y. Efficacy and safety of brexpiprazole for the treatment of acute schizophrenia in Japan: A 6-week, randomized, double-blind, placebo-controlled study. Psychiatry Clin Neurosci. 2018.

148. Jain S, Bhargava M, Gautam S. Weight gain with olanzapine: Drug, gender or age? Indian J Psychiatry. 2006;48(1):39-42.

149. Janenawasin S, Wang PW, Lembke A, Schumacher M, Das B, Santosa CM, et al. Olanzapine in diverse syndromal and subsyndromal exacerbations of bipolar disorders. Bipolar Disord. 2002;4(5):328-34.

150. Jeong JH, Bahk WM, Woo YS, Seo HJ, Hong SC, Jon DI, et al. Efficacy of quetiapine in patients with bipolar I and II depression: a multicenter, prospective, open-label, observational study. Neuropsychiatr Dis Treat. 2013;9:197-204.

151. Jessen K, Rostrup E, Mandl RCW, Nielsen MO, Bak N, Fagerlund B, et al. Cortical structures and their clinical correlates in antipsychotic-naive schizophrenia patients before and after 6 weeks of dopamine D2/3 receptor antagonist treatment. Psychol Med. 2019;49(5):754-63.

152. Jeste DV, Barak Y, Madhusoodanan S, Grossman F, Gharabawi G. International multisite double-blind trial of the atypical antipsychotics risperidone and olanzapine in 175 elderly patients with chronic schizophrenia. Am J Geriatr Psychiatry. 2003;11(6):638-47.

153. John JP, Chengappa KN, Baker RW, Gupta B, Mortimer MT. Assessment of changes in both weight and frequency of Use of medications for the treatment of gastro-instestinal symptoms among clozapine treated patients. Annals of Clinical Psychiatry 1995;7(3):1995.

154. Joseph AM, Venkatasubramanian G, Sharma PS. A six-to-ten weeks' follow-up study on the effects of olanzapine on abdominal fat and other metabolic parameters in patients with psychoses--an imaging-based study with controls. East Asian Arch Psychiatry. 2011;21(1):10-6.

155. Josiassen RC, Shaughnessy RA, Filymer DM, Donohue AM, Kacso M, Finkel N, et al. Early intervention with second-generation antipsychotics in first-episode psychosis: results of an 8-week naturalistic study. Early Interv Psychiatry. 2010;4(1):57-63.

156. Kahn RS, Fleischhacker WW, Boter H, Davidson M, Vergouwe Y, Keet IP, et al. Effectiveness of antipsychotic drugs in first-episode schizophrenia and schizophreniform disorder: an open randomised clinical trial. Lancet. 2008;371(9618):1085-97.

157. Kane JM, Carson WH, Saha AR, McQuade RD, Ingenito GG, Zimbroff DL, et al. Efficacy and safety of aripiprazole and haloperidol versus placebo in patients with schizophrenia and schizoaffective disorder. J Clin Psychiatry. 2002;63(9):763-71.

158. Kane JM, Crandall DT, Marcus RN, Eudicone J, Pikalov A, 3rd, Carson WH, et al. Symptomatic remission in schizophrenia patients treated with aripiprazole or haloperidol for up to 52 weeks. Schizophr Res. 2007;95(1-3):143-50.

159. Kane JM, Lauriello J, Laska E, Di Marino M, Wolfgang CD. Long-term efficacy and safety of iloperidone: results from 3 clinical trials for the treatment of schizophrenia. J Clin Psychopharmacol. 2008;28(2 Suppl 1):S29-35.

160. Kane JM, Cohen M, Zhao J, Alphs L, Panagides J. Efficacy and safety of asenapine in a placebo- and haloperidol-controlled trial in patients with acute exacerbation of schizophrenia. J Clin Psychopharmacol. 2010;30(2):106-15.

161. Kane JM, Detke HC, Naber D, Sethuraman G, Lin DY, Bergstrom RF, et al. Olanzapine long-acting injection: a 24-week, randomized, double-blind trial of maintenance treatment in patients with schizophrenia. Am J Psychiatry. 2010;167(2):181-9.

162. Kane JM, Mackle M, Snow-Adami L, Zhao J, Szegedi A, Panagides J. A randomized placebo-controlled trial of asenapine for the prevention of relapse of schizophrenia after long-term treatment. J Clin Psychiatry. 2011;72(3):349-55.

163. Kane JM, Potkin SG, Daniel DG, Buckley PF. A double-blind, randomized study comparing the efficacy and safety of sertindole and risperidone in patients with treatment-resistant schizophrenia. J Clin Psychiatry. 2011;72(2):194-204.

164. Kane JM, Peters-Strickland T, Baker RA, Hertel P, Eramo A, Jin N, et al. Aripiprazole once-monthly in the acute treatment of schizophrenia: findings from a 12-week, randomized, double-blind, placebo-controlled study. J Clin Psychiatry. 2014;75(11):1254-60.

165. Karagianis J, Grossman L, Landry J, Reed VA, de Haan L, Maguire GA, et al. A randomized controlled trial of the effect of sublingual orally disintegrating olanzapine versus oral olanzapine on body mass index: the PLATYPUS Study. Schizophr Res. 2009;113(1):41-8.

166. Karagianis J, Williams R, Davis L, Procyshyn R, Monga N, Hanley J, et al. Antipsychotic switching: results from a one-year prospective, observational study of patients with schizophrenia. Curr Med Res Opin. 2009;25(9):2121-32.

167. Karayal ON, Glue P, Bachinsky M, Stewart M, Chappell P, Kolluri S, et al. Switching from quetiapine to ziprasidone: a sixteen-week, open-label, multicenter study evaluating the effectiveness and safety of ziprasidone in outpatient subjects with schizophrenia or schizoaffective disorder. J Psychiatr Pract. 2011;17(2):100-9.

168. Kasper S, Lerman MN, McQuade RD, Saha A, Carson WH, Ali M, et al. Efficacy and safety of aripiprazole vs. haloperidol for long-term maintenance treatment following acute relapse of schizophrenia. Int J Neuropsychopharmacol. 2003;6(4):325-37.

169. Katagiri H, Tohen M, McDonnell DP, Fujikoshi S, Case M, Kanba S, et al. Efficacy and safety of olanzapine for treatment of patients with bipolar depression: Japanese subpopulation analysis of a randomized, double-blind, placebo-controlled study. BMC Psychiatry. 2013;13:138.

170. Keck PE, Jr., Marcus R, Tourkodimitris S, Ali M, Liebeskind A, Saha A, et al. A placebo-controlled, double-blind study of the efficacy and safety of aripiprazole in patients with acute bipolar mania. Am J Psychiatry. 2003;160(9):1651-8.

171. Keck PE, Jr., Calabrese JR, McIntyre RS, McQuade RD, Carson WH, Eudicone JM, et al. Aripiprazole monotherapy for maintenance therapy in bipolar I disorder: a 100-week, double-blind study versus placebo. J Clin Psychiatry. 2007;68(10):1480-91.

172. Keck PE, Orsulak PJ, Cutler AJ, Sanchez R, Torbeyns A, Marcus RN, et al. Aripiprazole monotherapy in the treatment of acute bipolar I mania: a randomized, double-blind, placebo- and lithium-controlled study. J Affect Disord. 2009;112(1-3):36-49.

173. Keefe RS, Young CA, Rock SL, Purdon SE, Gold JM, Breier A. One-year double-blind study of the neurocognitive efficacy of olanzapine, risperidone, and haloperidol in schizophrenia. Schizophr Res. 2006;81(1):1-15.

174. Kelly DL, Conley RR, Richardson CM, Tamminga CA, Carpenter WT, Jr. Adverse effects and laboratory parameters of high-dose olanzapine vs. clozapine in treatment-resistant schizophrenia. Ann Clin Psychiatry. 2003;15(3-4):181-6.

175. Kelly DL, Conley RR, Love RC, Morrison JA, McMahon RP. Metabolic risk with second-generation antipsychotic treatment: a double-blind randomized 8-week trial of risperidone and olanzapine. Ann Clin Psychiatry. 2008;20(2):71-8.

176. Kemp DE, Calabrese JR, Tran QV, Pikalov A, Eudicone JM, Baker RA. Metabolic syndrome in patients enrolled in a clinical trial of aripiprazole in the maintenance treatment of bipolar I disorder: a post hoc analysis of a randomized, double-blind, placebo-controlled trial. J Clin Psychiatry. 2010;71(9):1138-44.

177. Kim SW, Shin IS, Kim JM, Lee SH, Lee YH, Yang SJ, et al. Effects of switching to long-acting injectable risperidone from oral atypical antipsychotics on cognitive function in patients with schizophrenia. Hum Psychopharmacol. 2009;24(7):565-73.

178. Kim SW, Shin IS, Kim JM, Bae KY, Yang SJ, Yoon JS. Effectiveness of switching from aripiprazole to ziprasidone in patients with schizophrenia. Clin Neuropharmacol. 2010;33(3):121-5.

179. Kim SW, Yoon JS, Kim YS, Ahn YM, Kim CE, Go HJ, et al. The effect of paliperidone extended release on subjective well-being and responses in patients with schizophrenia. Prog Neuropsychopharmacol Biol Psychiatry. 2012;38(2):228-35.

180. Kim EY, Chang SM, Shim JC, Joo EJ, Kim JJ, Kim YS, et al. Long-term effectiveness of flexibly dosed paliperidone extended-release: comparison among patients with schizophrenia switching from risperidone and other antipsychotic agents. Curr Med Res Opin. 2013;29(10):1231-40.

181. Kingsbury SJ, Fayek M, Trufasiu D, Zada J, Simpson GM. The apparent effects of ziprasidone on plasma lipids and glucose. J Clin Psychiatry. 2001;62(5):347-9.

182. Kingstone E, Kolivakis T, Kossatz I. Double blind study of clopenthixol and chlorpromazine in acute hospitalized schizophhrenics. Internationale Zeitschrift fur Klinische Pharmakologie, Therapie und Toxikologie. 1970;3(1):41 - 5.

183. Kinon BJ, Basson BR, Gilmore JA, Malcolm S, Stauffer VL. Strategies for switching from conventional antipsychotic drugs or risperidone to olanzapine. J Clin Psychiatry. 2000;61(11):833-40.

184. Kinon BJ, Hill AL, Liu H, Kollack-Walker S. Olanzapine orally disintegrating tablets in the treatment of acutely ill non-compliant patients with schizophrenia. Int J Neuropsychopharmacol. 2003;6(2):97-102.

185. Kinon BJ, Jeste DV, Kollack-Walker S, Stauffer V, Liu-Seifert H. Olanzapine treatment for tardive dyskinesia in schizophrenia patients: a prospective clinical trial with patients randomized to blinded dose reduction periods. Prog Neuropsychopharmacol Biol Psychiatry. 2004;28(6):985-96.

186. Kinon BJ, Lipkovich I, Edwards SB, Adams DH, Ascher-Svanum H, Siris SG. A 24-week randomized study of olanzapine versus ziprasidone in the treatment of schizophrenia or schizoaffective disorder in patients with prominent depressive symptoms. J Clin Psychopharmacol. 2006;26(2):157-62.

187. Kinon BJ, Kollack-Walker S, Jeste D, Gupta S, Chen L, Case M, et al. Incidence of tardive dyskinesia in older adult patients treated with olanzapine or conventional antipsychotics. J Geriatr Psychiatry Neurol Journal Translated Name Journal of Geriatric Psychiatry and Neurology. 2015;28(1):67-79.

188. Kinoshita T, Bai YM, Kim JH, Miyake M, Oshima N. Efficacy and safety of asenapine in Asian patients with an acute exacerbation of schizophrenia: a multicentre, randomized, double-blind, 6-week, placebo-controlled study. Psychopharmacology (Berl). 2016;233(14):2663-74.

189. Kissling W, Glue P, Medori R, Simpson S. Long-term safety and efficacy of long-acting risperidone in elderly psychotic patients. Hum Psychopharmacol. 2007;22(8):505-13.

190. Kivircik BB, Alptekin K, Caliskan S, Comlekci A, Oruk G, Tumuklu M, et al. Effect of clozapine on serum leptin, insulin levels, and body weight and composition in patients with schizophrenia. Prog Neuropsychopharmacol Biol Psychiatry. 2003;27(5):795-9.

191. Kluge M, Schuld A, Himmerich H, Dalal M, Schacht A, Wehmeier PM, et al. Clozapine and olanzapine are associated with food craving and binge eating: results from a randomized double-blind study. J Clin Psychopharmacol. 2007;27(6):662-6.

192. Kluge M, Schuld A, Schacht A, Himmerich H, Dalal MA, Wehmeier PM, et al. Effects of clozapine and olanzapine on cytokine systems are closely linked to weight gain and drug-induced fever. Psychoneuroendocrinology. 2009;34(1):118-28.

193. Krakowski M, Czobor P, Citrome L. Weight gain, metabolic parameters, and the impact of race in aggressive inpatients randomized to double-blind clozapine, olanzapine or haloperidol. Schizophr Res. 2009;110(1-3):95-102.

194. Kraus T, Haack M, Schuld A, Hinze-Selch D, Kuhn M, Uhr M, et al. Body weight and leptin plasma levels during treatment with antipsychotic drugs. Am J Psychiatry. 1999;156(2):312-4.

195. Kumar PBS, Pandey RS, Thirthalli J, Kumar PTS, Kumar CN. A Comparative Study of Short Term Efficacy of Aripiprazole and Risperidone in Schizophrenia. Curr Neuropharmacol. 2017;15(8):1073 - 84.

196. Kusumi I, Honda M, Uemura K, Sugawara Y, Kohsaka M, Tochigi A, et al. Effect of olanzapine orally disintegrating tablet versus oral standard tablet on body weight in patients with schizophrenia: a randomized open-label trial. Prog Neuropsychopharmacol Biol Psychiatry. 2012;36(2):313-7.

197. Lal S, Thavundayil JX, Nair NP, Annable L, Ng Ying Kin NM, Gabriel A, et al. Levomepromazine versus chlorpromazine in treatment-resistant schizophrenia: a double-blind randomized trial. J Psychiatry Neurosci. 2006;31(4):271-9.

198. Lambert M, Haro JM, Novick D, Edgell ET, Kennedy L, Ratcliffe M, et al. Olanzapine vs. other antipsychotics in actual out-patient settings: six months tolerability results from the European Schizophrenia Out-patient Health Outcomes study. Acta Psychiatr Scand. 2005;111(3):232-43.

199. Lamberti JS, Bellnier T, Schwarzkopf SB. Weight gain among schizophrenic patients treated with clozapine. Am J Psychiatry. 1992;149(5):689-90.

200. Lane HY, Chang YC, Cheng YC, Liu GC, Lin XR, Chang WH. Effects of patient demographics, risperidone dosage, and clinical outcome on body weight in acutely exacerbated schizophrenia. J Clin Psychiatry. 2003;64(3):316-20.

201. Lane HY, Chang YC, Chiu CC, Lee SH, Lin CY, Chang WH. Fine-tuning risperidone dosage for acutely exacerbated schizophrenia: clinical determinants. Psychopharmacology (Berl). 2004;172(4):393-9.

202. Lasser RA, Bossie CA, Gharabawi GM, Turner M. Patients with schizophrenia previously stabilized on conventional depot antipsychotics experience significant clinical improvements following treatment with long-acting risperidone. Eur Psychiatry. 2004;19(4):219-25.

203. Lasser R, Bossie CA, Gharabawi G, Eerdekens M, Nasrallah HA. Efficacy and safety of long-acting risperidone in stable patients with schizoaffective disorder. J Affect Disord. 2004;83(2-3):263-75.

204. Lasser RA, Mao L, Gharabawi G. Smokers and nonsmokers equally affected by olanzapine-induced weight gain: metabolic implications. Schizophr Res. 2004;66(2-3):163-7.

205. Lauriello J, Lambert T, Andersen S, Lin D, Taylor CC, McDonnell D. An 8-week, double-blind, randomized, placebo-controlled study of olanzapine long-acting injection in acutely ill patients with schizophrenia. J Clin Psychiatry. 2008;69(5):790-9.

206. Lecrubier Y, Quintin P, Bouhassira M, Perrin E, Lancrenon S. The treatment of negative symptoms and deficit states of chronic schizophrenia: olanzapine compared to amisulpride and placebo in a 6-month double-blind controlled clinical trial. Acta Psychiatr Scand. 2006;114(5):319-27.

207. Lee CT, Conde BJ, Mazlan M, Visanuyothin T, Wang A, Wong MM, et al. Switching to olanzapine from previous antipsychotics: a regional collaborative multicenter trial assessing 2 switching techniques in Asia Pacific. J Clin Psychiatry. 2002;63(7):569-76.

208. Lee KU, Jeon YW, Lee HK, Jun TY. Efficacy and safety of quetiapine for depressive symptoms in patients with schizophrenia. Hum Psychopharmacol. 2009;24(6):447-52.

209. Lee HY, Ham BJ, Kang RH, Paik JW, Hahn SW, Lee MS, et al. Trial of aripiprazole in the treatment of first-episode schizophrenia. Psychiatry Clin Neurosci. 2010;64(1):38-43.

210. Lee JS, Ahn JH, Lee JI, Kim JH, Jung I, Lee CU, et al. Dose pattern and effectiveness of paliperidone extended-release tablets in patients with schizophrenia. Clin Neuropharmacol. 2011;34(5):186-90.

211. Lee SJ, Lee JH, Jung SW, Koo BH, Choi TY, Lee KH. A 6-Week, Randomized, Multicentre, Open-Label Study Comparing Efficacy and Tolerability of Amisulpride at a Starting Dose of 400 mg/day versus 800 mg/day in Patients with Acute Exacerbations of Schizophrenia. Clinical Drug Investigation. 2012;32:735 - 45.

212. Lee HB, Yoon BH, Kwon YJ, Woo YS, Lee JG, Kim MD, et al. The Efficacy and Safety of Switching to Ziprasidone from Olanzapine in Patients with Bipolar I Disorder: An 8-Week, Multicenter, Open-Label Study. Clin Drug Investig. 2013;33:743 - 53.

213. Leelahanaj T, Kongsakon R, Netrakom P. A 4-week, double-blind comparison of olanzapine with haloperidol in the treatment of amphetamine psychosis. Journal of the Medical Association of Thailand = Chotmaihet thangphaet. 2005;88 Suppl 3:S43-52.

214. Li YM, Zhao JP, Ou JJ, Wu RR. Efficacy and tolerability of ziprasidone vs. olanzapine in naive first-episode schizophrenia: a 6-week, randomized, open-label, flexible-dose study. Pharmacopsychiatry. 2012;45(5):177-81.

215. Li H, Luo J, Wang C, Xie S, Xu X, Wang X, et al. Efficacy and safety of aripiprazole in Chinese Han schizophrenia subjects: a randomized, double-blind, active parallel-controlled, multicenter clinical trial. Schizophr Res. 2014;157(1-3):112-9.

216. Li H, Turkoz I, Zhang F. Efficacy and safety of once-monthly injection of paliperidone palmitate in hospitalized Asian patients with acute exacerbated schizophrenia: an open-label, prospective, noncomparative study. Neuropsychiatr Dis Treat. 2016;12:15-24.

217. Lieberman JA, Stroup TS, McEvoy JP, Swartz MS, Rosenheck RA, Perkins DO, et al. Effectiveness of antipsychotic drugs in patients with chronic schizophrenia. N Engl J Med. 2005;353(12):1209-23.

218. Lieberman JA, Davis RE, Correll CU, Goff DC, Kane JM, Tamminga CA, et al. ITI-007 for the Treatment of Schizophrenia: A 4-Week Randomized, Double-Blind, Controlled Trial. Biol Psychiatry. 2016;79(12):952-61.

219. Lin CC, Bai YM, Wang YC, Chen TT, Lai IC, Chen JY, et al. Improved body weight and metabolic outcomes in overweight or obese psychiatric patients switched to amisulpride from other atypical antipsychotics. J Clin Psychopharmacol. 2009;29(6):529-36.

220. Lin CC, Chiu HJ, Chen JY, Liou YJ, Wang YC, Chen TT, et al. Switching from clozapine to zotepine in patients with schizophrenia: a 12-week prospective, randomized, rater blind, and parallel study. J Clin Psychopharmacol. 2013;33(2):211-4.

221. Lin CH, Wang FC, Lin SC, Huang YH, Chen CC. A randomized, double-blind, comparison of the efficacy and safety of low-dose olanzapine plus low-dose trifluoperazine versus full-dose olanzapine in the acute treatment of schizophrenia. Schizophr Res. 2017;185:80-7.

222. Lindenmayer JP, Volavka J, Lieberman J, Sheitman B, Citrome L, Chakos M, et al. Olanzapine for schizophrenia refractory to typical and atypical antipsychotics: an open-label, prospective trial. J Clin Psychopharmacol. 2001;21(4):448-53.

223. Lindenmayer JP, Czobor P, Volavka J, Lieberman JA, Citrome L, Sheitman B, et al. Olanzapine in refractory schizophrenia after failure of typical or atypical antipsychotic treatment: an open-label switch study. J Clin Psychiatry. 2002;63(10):931-5.

224. Lindenmayer JP, Eerdekens E, Berry SA, Eerdekens M. Safety and efficacy of long-acting risperidone in schizophrenia: a 12-week, multicenter, open-label study in stable patients switched from typical and atypical oral antipsychotics. J Clin Psychiatry. 2004;65(8):1084-9.

225. Lindenmayer JP, Khan A, Iskander A, Abad MT, Parker B. A randomized controlled trial of olanzapine versus haloperidol in the treatment of primary negative symptoms and neurocognitive deficits in schizophrenia. J Clin Psychiatry. 2007;68(3):368-79.

226. Lindenmayer JP, Khan A, Eerdekens M, Van Hove I, Kushner S. Long-term safety and tolerability of long-acting injectable risperidone in patients with schizophrenia or schizoaffective disorder. Eur Neuropsychopharmacol. 2007;17(2):138-44.

227. Lindenmayer JP, Citrome L, Khan A, Kaushik S. A randomized, double-blind, parallel-group, fixed-dose, clinical trial of quetiapine at 600 versus 1200 mg/d for patients with treatment-resistant schizophrenia or schizoaffective disorder. J Clin Psychopharmacol. 2011;31(2):160-8.

228. Littrell KH, Petty RG, Hilligoss NM, Peabody CD, Johnson CG. Olanzapine treatment for patients with schizophrenia and substance abuse. J Subst Abuse Treat. 2001;21(4):217-21.

229. Llorca PM, Lancon C, Disdier B, Farisse J, Sapin C, Auquier P. Effectiveness of clozapine in neuroleptic-resistant schizophrenia: clinical response and plasma concentrations. J Psychiatry Neurosci. 2002;27(1):30-7.

230. Loebel A, Cucchiaro J, Sarma K, Xu L, Hsu C, Kalali AH, et al. Efficacy and safety of lurasidone 80 mg/day and 160 mg/day in the treatment of schizophrenia: a randomized, double-blind, placebo- and active-controlled trial. Schizophr Res. 2013;145(1-3):101-9.

231. Loebel A, Cucchiaro J, Xu J, Sarma K, Pikalov A, Kane JM. Effectiveness of lurasidone vs. quetiapine XR for relapse prevention in schizophrenia: a 12-month, double-blind, noninferiority study. Schizophr Res. 2013;147(1):95-102.

232. Loebel A, Cucchiaro J, Silva R, Kroger H, Hsu J, Sarma K, et al. Lurasidone monotherapy in the treatment of bipolar I depression: a randomized, double-blind, placebo-controlled study. Am J Psychiatry. 2014;171(2):160-8.

233. Loebl T, Angarita GA, Pachas GN, Huang KL, Lee SH, Nino J, et al. A randomized, double-blind, placebo-controlled trial of long-acting risperidone in cocaine-dependent men. J Clin Psychiatry. 2008;69(3):480-6.

234. Malempati RN, Bond DJ, Yatham LN. Depot risperidone in the outpatient management of bipolar disorder: a 2-year study of 10 patients. Int Clin Psychopharmacol. 2008;23(2):88-94.

235. Marder SR, Kramer M, Ford L, Eerdekens E, Lim P, Eerdekens M, et al. Efficacy and safety of paliperidone extended-release tablets: results of a 6-week, randomized, placebo-controlled study. Biol Psychiatry. 2007;62(12):1363-70.

236. Martin S, Ljo H, Peuskens J, Thirumalai S, Giudicelli A, Fleurot O, et al. A double-blind, randomised comparative trial of amisulpride versus olanzapine in the treatment of schizophrenia: short-term results at two months. Curr Med Res Opin. 2002;18(6):355-62.

237. Mauri MC, Laini V, Boscati L, Rudelli R, Salvi V, Orlandi R, et al. Long-term treatment of chronic schizophrenia with risperidone: a study with plasma levels. Eur Psychiatry. 2001;16(1):57-63.

238. Mauri MC, Steinhilber CP, Marino R, Invernizzi E, Fiorentini A, Cerveri G, et al. Clinical outcome and olanzapine plasma levels in acute schizophrenia. Eur Psychiatry. 2005;20(1):55-60.

239. Mauri MC, Colasanti A, Rossattini M, Volonteri LS, Dragogna F, Fiorentini A, et al. Ziprasidone outcome and tolerability: a practical clinical trial with plasma drug levels. Pharmacopsychiatry. 2007;40(3):89-92.

240. Mazeh D, Paleacu D, Barak Y. Quetiapine for elderly non-responsive schizophrenia patients. Psychiatry Res. 2008;157(1-3):265-7.

241. McElroy SL, Suppes T, Frye MA, Altshuler LL, Stanford K, Martens B, et al. Open-label aripiprazole in the treatment of acute bipolar depression: a prospective pilot trial. J Affect Disord. 2007;101(1-3):275-81.

242. McElroy SL, Nelson EB, Welge JA, Kaehler L, Keck PE, Jr. Olanzapine in the treatment of pathological gambling: a negative randomized placebo-controlled trial. J Clin Psychiatry. 2008;69(3):433-40.

243. McEvoy JP, Daniel DG, Carson WH, Jr., McQuade RD, Marcus RN. A randomized, double-blind, placebo-controlled, study of the efficacy and safety of aripiprazole 10, 15 or 20 mg/day for the treatment of patients with acute exacerbations of schizophrenia. J Psychiatr Res. 2007;41(11):895-905.

244. McEvoy JP, Lieberman JA, Perkins DO, Hamer RM, Gu H, Lazarus A, et al. Efficacy and tolerability of olanzapine, quetiapine, and risperidone in the treatment of early psychosis: a randomized, double-blind 52-week comparison. Am J Psychiatry. 2007;164(7):1050-60.

245. McEvoy JP, Citrome L, Hernandez D, Cucchiaro J, Hsu J, Pikalov A, et al. Effectiveness of lurasidone in patients with schizophrenia or schizoaffective disorder switched from other antipsychotics: a randomized, 6-week, open-label study. J Clin Psychiatry. 2013;74(2):170-9.

246. McEvoy JP, Byerly M, Hamer RM, Dominik R, Swartz MS, Rosenheck RA, et al. Effectiveness of paliperidone palmitate vs haloperidol decanoate for maintenance treatment of schizophrenia: a randomized clinical trial. JAMA. 2014;311(19):1978-87.

247. McFadden W, Ma Y-W, Haskins T, Bossie C, Alphs L. A Prospective Study Comparing the Long-term Effectiveness of Injectable Risperidone Long-acting Therapy and Oral Aripiprazole in Patients with Schizophrenia. Psychiatry (Edgmont). 2010;7(11):23 - 31.

248. McGlashan TH, Zipursky RB, Perkins D, Addington J, Miller T, Woods SW, et al. Randomized, double-blind trial of olanzapine versus placebo in patients prodromally symptomatic for psychosis. Am J Psychiatry. 2006;163(5):790-9.

249. McIntyre RS, Mancini DA, Basile VS, Srinivasan J, Kennedy SH. Antipsychotic-induced weight gain: bipolar disorder and leptin. J Clin Psychopharmacol. 2003;23(4):323-7.

250. McIntyre RS, Trakas K, Lin D, Balshaw R, Hwang P, Robinson K, et al. Risk of weight gain associated with antipsychotic treatment: results from the Canadian National Outcomes Measurement Study in Schizophrenia. Can J Psychiatry. 2003;48(10):689-94.

251. McIntyre RS, Mancini DA, Srinivasan J, McCann S, Konarski JZ, Kennedy SH. The antidepressant effects of risperidone and olanzapine in bipolar disorder. Can J Clin Pharmacol. 2004;11(2):e218-26.

252. McIntyre RS, Cohen M, Zhao J, Alphs L, Macek TA, Panagides J. A 3-week, randomized, placebo-controlled trial of asenapine in the treatment of acute mania in bipolar mania and mixed states. Bipolar Disord. 2009;11(7):673-86.

253. McIntyre RS, Cohen M, Zhao J, Alphs L, Macek TA, Panagides J. Asenapine in the treatment of acute mania in bipolar I disorder: a randomized, double-blind, placebo-controlled trial. J Affect Disord. 2010;122(1-2):27-38.

254. Meltzer HY, Perry E, Jayathilake K. Clozapine-induced weight gain predicts improvement in psychopathology. Schizophr Res. 2003;59(1):19-27.

255. Meltzer HY, Bobo WV, Roy A, Jayathilake K, Chen Y, Ertugrul A, et al. A randomized, double-blind comparison of clozapine and high-dose olanzapine in treatment-resistant patients with schizophrenia. J Clin Psychiatry. 2008;69(2):274-85.

256. Meltzer HY, Bobo WV, Lee MA, Cola P, Jayathilake K. A randomized trial comparing clozapine and typical neuroleptic drugs in non-treatment-resistant schizophrenia. Psychiatry Res. 2010;177(3):286-93.

257. Meltzer HY, Cucchiaro J, Silva R, Ogasa M, Phillips D, Xu J, et al. Lurasidone in the treatment of schizophrenia: a randomized, double-blind, placebo- and olanzapine-controlled study. Am J Psychiatry. 2011;168(9):957-67.

258. Merideth C, Cutler AJ, She F, Eriksson H. Efficacy and tolerability of extended release quetiapine fumarate monotherapy in the acute treatment of generalized anxiety disorder: a randomized, placebo controlled and active-controlled study. Int Clin Psychopharmacol. 2012;27(1):40-54.

259. Miller DD, Ellingrod VL, Holman TL, Buckley PF, Arndt S. Clozapine-induced weight gain associated with the 5HT2C receptor -759C/T polymorphism. Am J Med Genet B Neuropsychiatr Genet. 2005;133B(1):97-100.

260. Moller HJ, Llorca PM, Sacchetti E, Martin SD, Medori R, Parellada E. Efficacy and safety of direct transition to risperidone long-acting injectable in patients treated with various antipsychotic therapies. Int Clin Psychopharmacol. 2005;20(3):121-30.

261. Mohl A, Westlye K, Opjordsmoen S, Lex A, Schreiner A, Benoit M, et al. Long-acting risperidone in stable patients with schizoaffective disorder. J Psychopharmacol. 2005;19(5 Suppl):22-31.

262. Molina L, Recinos B, Paz B, Rovelo M, Elias Rodriguez FE, Calderon J, et al. Factors Related to Early Clinical Effects of Quetiapine Extended-Release: A Multinational, Prospective, Observational Study. Clin Drug Investig. 2016;36(6):491-7.

263. Montejo Gonzalez A, Rico-Villademoros F, Tafalla M, Majadas S. A 6-month prospective observational study on the effects quatiapine on sexual functioning. Journal of Clinical Psychopharmacology. 2005;25(6):533 - 8.

264. Moretti R, Torre P, Antonello RM, Cattaruzza T, Cazzato G, Bava A. Olanzapine as a possible treatment for anxiety due to vascular dementia: an open study. Am J Alzheimers Dis Other Demen. 2004;19(2):81-8.

265. Mortimer A, Martin S, Loo H, Peuskens J, Group SS. A double-blind, randomized comparative trial of amisulpride versus olanzapine for 6 months in the treatment of schizophrenia. Int Clin Psychopharmacol. 2004;19(2):63-9.

266. Muller DJ, Klempan TA, De Luca V, Sicard T, Volavka J, Czobor P, et al. The SNAP-25 gene may be associated with clinical response and weight gain in antipsychotic treatment of schizophrenia. Neurosci Lett. 2005;379(2):81-9.

267. Murashita M, Kusumi I, Inoue T, Takahashi Y, Hosoda H, Kangawa K, et al. Olanzapine increases plasma ghrelin level in patients with schizophrenia. Psychoneuroendocrinology. 2005;30(1):106-10.

268. Na KS, Kim WH, Jung HY, Ryu SG, Min KJ, Park KC, et al. Relationship between inflammation and metabolic syndrome following treatment with paliperidone for schizophrenia. Prog Neuropsychopharmacol Biol Psychiatry. 2012;39(2):295-300.

269. Naber D, Hansen K, Forray C, Baker RA, Sapin C, Beillat M, et al. Qualify: a randomized head-to-head study of aripiprazole once-monthly and paliperidone palmitate in the treatment of schizophrenia. Schizophr Res. 2015;168(1-2):498-504.

270. Nagesh HN, Kumar A, Kishore MS, Kumar N. A randomized prospective comparative study of weight gain between asenapine and iloperidone in patients with psychosis. National Journal of Physiology, Pharmacy and Pharmacology. 2017;7(94 - 98 ).

271. Nasrallah HA, Gopal S, Gassmann-Mayer C, Quiroz JA, Lim P, Eerdekens M, et al. A controlled, evidence-based trial of paliperidone palmitate, a long-acting injectable antipsychotic, in schizophrenia. Neuropsychopharmacology. 2010;35(10):2072-82.

272. Nasrallah HA, Silva R, Phillips D, Cucchiaro J, Hsu J, Xu J, et al. Lurasidone for the treatment of acutely psychotic patients with schizophrenia: a 6-week, randomized, placebo-controlled study. J Psychiatr Res. 2013;47(5):670-7.

273. Nasrallah HA, Newcomer JW, Risinger R, Du Y, Zummo J, Bose A, et al. Effect of Aripiprazole Lauroxil on Metabolic and Endocrine Profiles and Related Safety Considerations Among Patients With Acute Schizophrenia. J Clin Psychiatry. 2016;77(11):1519-25.

274. Nasrallah HA, Earley W, Cutler AJ, Wang Y, Lu K, Laszlovszky I, et al. The safety and tolerability of cariprazine in long-term treatment of schizophrenia: a post hoc pooled analysis. BMC Psychiatry. 2017;17(1):305.

275. Neovius M, Eberhard J, Lindstrom E, Levander S. Weight development in patients treated with risperidone: a 5-year naturalistic study. Acta Psychiatr Scand. 2007;115(4):277-85.

276. Newcomer JW, Campos JA, Marcus RN, Breder C, Berman RM, Kerselaers W, et al. A multicenter, randomized, double-blind study of the effects of aripiprazole in overweight subjects with schizophrenia or schizoaffective disorder switched from olanzapine. J Clin Psychiatry. 2008;69(7):1046-56.

277. Newcomer JW, Ratner RE, Eriksson JW, Emsley R, Meulien D, Miller F, et al. A 24-week, multicenter, open-label, randomized study to compare changes in glucose metabolism in patients with schizophrenia receiving treatment with olanzapine, quetiapine, or risperidone. J Clin Psychiatry. 2009;70(4):487-99.

278. Nick B, Vauth R, Braendle D, Riecher-Rossler A. Symptom control, functioning and satisfaction among Swiss patients treated with risperidone long-acting injectable. International Journal of Psychiatry and Clinical Practice. 2005;10(3):174 - 81.

279. Nielsen MO, Rostrup E, Wulff S, Glenthoj B, Ebdrup BH. Striatal Reward Activity and Antipsychotic-Associated Weight Change in Patients With Schizophrenia Undergoing Initial Treatment. JAMA Psychiatry. 2016;73(2):121-8.

280. Ogasa M, Kimura T, Nakamura M, Guarino J. Lurasidone in the treatment of schizophrenia: a 6-week, placebo-controlled study. Psychopharmacology (Berl). 2013;225(3):519-30.

281. Osser DN, Najarian DM, Dufresne RL. Olanzapine increases weight and serum triglyceride levels. J Clin Psychiatry. 1999;60(11):767-70.

282. Monreal Ortiz J, Chesa Vela D, Surribas Lez E, Franquelo Cruz C. THe Effects of Amisulpride on weight and metabolic parameters. Eur Neuropsychopharmacol. 2009:S548-S9.

283. Ou JJ, Xu Y, Chen HH, Fan X, Gao K, Wang J, et al. Comparison of metabolic effects of ziprasidone versus olanzapine treatment in patients with first-episode schizophrenia. Psychopharmacoly. 2013;225:727 - 635.

284. Ozguven HD, Baskak B, Oner O, Atbasoglu C. Metabolic effects of olanzapine and quetiapine: a six week randomized, single blind, controlled study. 2011. 2011;4:10 - 7.

285. Pae CU, Serretti A, Chiesa A, Mandelli L, Lee C, Kim J, et al. Immediate versus gradual suspension of previous treatments during switch to aripiprazole: results of a randomized, open label study. Eur Neuropsychopharmacol. 2009;19(8):562-70.

286. Pandina G, Lane R, Gopal S, Gassmann-Mayer C, Hough D, Remmerie B, et al. A double-blind study of paliperidone palmitate and risperidone long-acting injectable in adults with schizophrenia. Prog Neuropsychopharmacol Biol Psychiatry. 2011;35(1):218-26.

287. Papakostas GI, Vitolo OV, Ishak WW, Rapaport MH, Zajecka JM, Kinrys G, et al. A 12-week, randomized, double-blind, placebo-controlled, sequential parallel comparison trial of ziprasidone as monotherapy for major depressive disorder. J Clin Psychiatry. 2012;73(12):1541-7.

288. Parellada E, Andrezina R, Milanova V, Glue P, Masiak M, Turner MS, et al. Patients in the early phases of schizophrenia and schizoaffective disorders effectively treated with risperidone long-acting injectable. J Psychopharmacol. 2005;19(5 Suppl):5-14.

289. Parellada E, Kouniakis F, Siurkute A, Schreiner A, Don L. Safety and efficacy of long-acting injectable risperidone in daily practice: an open-label, noninterventional, prospective study in schizophrenia and related disorders. Int Clin Psychopharmacol. 2010;25(3):149-54.

290. Park YM, Chung YC, Lee SH, Lee KJ, Kim H, Byun YC, et al. Weight gain associated with the alpha2a-adrenergic receptor -1,291 C/G polymorphism and olanzapine treatment. Am J Med Genet B Neuropsychiatr Genet. 2006;141B(4):394-7.

291. Perez-Iglesias R, Crespo-Facorro B, Amado JA, Garcia-Unzueta MT, Ramirez-Bonilla ML, Gonzalez-Blanch C, et al. A 12-week randomized clinical trial to evaluate metabolic changes in drug-naive, first-episode psychosis patients treated with haloperidol, olanzapine, or risperidone. J Clin Psychiatry. 2007;68(11):1733-40.

292. Perez-Iglesias R, Crespo-Facorro B, Martinez-Garcia O, Ramirez-Bonilla ML, Alvarez-Jimenez M, Pelayo-Teran JM, et al. Weight gain induced by haloperidol, risperidone and olanzapine after 1 year: findings of a randomized clinical trial in a drug-naive population. Schizophr Res. 2008;99(1-3):13-22.

293. Perez-Iglesias R, Ortiz-Garcia de la Foz V, Martinez Garcia O, Amado JA, Garcia-Unzueta MT, Ayesa-Arriola R, et al. Comparison of metabolic effects of aripiprazole, quetiapine and ziprasidone after 12 weeks of treatment in first treated episode of psychosis. Schizophr Res. 2014;159(1):90-4.

294. Perlis RH, Baker RW, Zarate CA, Jr., Brown EB, Schuh LM, Jamal HH, et al. Olanzapine versus risperidone in the treatment of manic or mixed States in bipolar I disorder: a randomized, double-blind trial. J Clin Psychiatry. 2006;67(11):1747-53.

295. Peuskens J, Bech P, Moller HJ, Bale R, Fleurot O, Rein W. Amisulpride vs. risperidone in the treatment of acute exacerbations of schizophrenia. Amisulpride study group. Psychiatry Res. 1999;88(2):107-17.

296. Peuskens J, De Hert M, Mortimer A. Metabolic control in patients with schizophrenia treated with amisulpride or olanzapine. Int Clin Psychopharmacol. 2007;22(3):145-52.

297. Peuskens J, Gillain B, De Graeve D, Van Vleymen B, Albert A. Belgian Schizophrenia Outcome Survey - results of a 2-year naturalistic study in patients stabilised on monotherapy with olanzapine, risperidone or haloperidol. Eur Psychiatry. 2009;24(3):154-63.

298. Pigott TA, Carson WH, Saha AR, Torbeyns AF, Stock EG, Ingenito GG, et al. Aripiprazole for the prevention of relapse in stabilized patients with chronic schizophrenia: a placebo-controlled 26-week study. J Clin Psychiatry. 2003;64(9):1048-56.

299. Popovic V, Doknic M, Maric N, Pekic S, Damjanovic A, Miljic D, et al. Changes in neuroendocrine and metabolic hormones induced by atypical antipsychotics in normal-weight patients with schizophrenia. Neuroendocrinology. 2007;85(4):249-56.

300. Potkin SG, Saha AR, Kujawa MJ, Carson WH, Ali M, Stock E, et al. Aripiprazole, an antipsychotic with a novel mechanism of action, and risperidone vs placebo in patients with schizophrenia and schizoaffective disorder. Arch Gen Psychiatry. 2003;60(7):681-90.

301. Potkin SG, Gharabawi GM, Greenspan AJ, Mahmoud R, Kosik-Gonzalez C, Rupnow MF, et al. A double-blind comparison of risperidone, quetiapine and placebo in patients with schizophrenia experiencing an acute exacerbation requiring hospitalization. Schizophr Res. 2006;85(1-3):254-65.

302. Potvin S, Morin M, Cloutier C, Gendron A, Bissonnette A, Marchand S. Add-on treatment of quetiapine for fibromyalgia: a pilot, randomized, double-blind, placebo-controlled 12-week trial. J Clin Psychopharmacol. 2012;32(5):684-7.

303. Quiroz JA, Yatham LN, Palumbo JM, Karcher K, Kushner S, Kusumakar V. Risperidone long-acting injectable monotherapy in the maintenance treatment of bipolar I disorder. Biol Psychiatry. 2010;68(2):156-62.

304. Ratner Y, Gibel A, Yorkov V, Ritsner MS. Effectiveness, safety, and tolerability of ziprasidone for treating schizophrenia patients undergoing usual care: a 12-month, open-label, flexible-dose, naturalistic observational trial. Prog Neuropsychopharmacol Biol Psychiatry. 2007;31(7):1401-9.

305. Reich DB, Winternitz S, Hennen J, Watts T, Stanculescu C. A preliminary study of risperidone in the treatment of posttraumatic stress disorder related to childhood abuse in women. J Clin Psychiatry. 2004;65(12):1601-6.

306. Rettenbacher M, Baumgartner S, Eder-Ischia U, Edlinger M, Graziadei I, Hofer A, et al. Association between antipsychotic-induced elevation of liver enzymes and weight gain. Journal of Clinical Psychopharmacology. 2007;26(5):500 - 3.

307. Revicky DA, Genduso LA, Hamilton AH, Ganzoczy D, Beasly Jr CM. Olanzapine versus haloperidol in thetreatment of schizophrenia and other psychotic disorders: Quality of life and clinical outcomes of a randomized clinical trial. Quality of Life Research. 1999;8:417-26.

308. Riedel M, Muller N, Strassnig M, Spellmann I, Engel RR, Musil R, et al. Quetiapine has equivalent efficacy and superior tolerability to risperidone in the treatment of schizophrenia with predominantly negative symptoms. Eur Arch Psychiatry Clin Neurosci. 2005;255(6):432-7.

309. Rodrigues Louza M, Elkis H, Ruschel S, Reis de Oliveira I, Affonseca Bressan R, Belmonte-de-Abreu P, et al. Long-acting injectable risperidone in partially adherent and non-adherent patients with schizophrenia. Neuropsychiatric Disease and Treatment. 2011;7:391 - 8.

310. Rodriguez-Perez V, Lopez A, Blanco C, Pena C, Abel A, Gomez Y, et al. Olanzapine for the treatment of chronic refractory schizophrenia: a 12-month follow-up naturalistic study. Prog Neuropsychopharmacol Biol Psychiatry. 2002;26(6):1055-62.

311. Roerig JL, Steffen KJ, Mitchell JE, Crosby RD, Gosnell BA. A comparison of the effects of olanzapine and risperidone versus placebo on ghrelin plasma levels. J Clin Psychopharmacol. 2008;28(1):21-6.

312. Rosa F, Schreiner A, Thomas P, Sherif T. Switching patients with stable schizophrenia or schizoaffective disorder from olanzapine to risperidone long-acting injectable. Clin Drug Investig. 2012;32(4):267-79.

313. Rossi A, Bagala A, Del Curatolo V, Scapati F, Bernareggi MM, Giustra MG. Remission in schizophrenia: one-year Italian prospective study of risperidone long-acting injectable (RLAI) in patients with schizophrenia or schizoaffective disorder. Hum Psychopharmacol. 2009;24(7):574-83.

314. Sacchetti E, Valsecchi P, Parrinello G. A randomized, flexible-dose, quasi-naturalistic comparison of quetiapine, risperidone, and olanzapine in the short-term treatment of schizophrenia: the QUERISOLA trial. Schizophr Res. 2008;98(1-3):55-65.

315. Sacchetti E, Galluzzo A, Valsecchi P, Romeo F, Gorini B, Warrington L. Ziprasidone vs clozapine in schizophrenia patients refractory to multiple antipsychotic treatments: the MOZART study. Schizophr Res. 2009;113(1):112-21.

316. Sachs G, Sanchez R, Marcus R, Stock E, McQuade R, Carson W, et al. Aripiprazole in the treatment of acute manic or mixed episodes in patients with bipolar I disorder: a 3-week placebo-controlled study. J Psychopharmacol. 2006;20(4):536-46.

317. Saddichha S, Manjunatha N, Ameen S, Akhtar S. Diabetes and schizophrenia - effect of disease or drug? Results from a randomized, double-blind, controlled prospective study in first-episode schizophrenia. Acta Psychiatr Scand. 2008;117(5):342-7.

318. Sajatovic M, Brescan DW, Perez DE, DiGiovanni SK, Hattab H, Ray JB, et al. Quetiapine alone and added to a mood stabilizer for serious mood disorders. J Clin Psychiatry. 2001;62(9):728-32.

319. Sajatovic M, Coconcea N, Ignacio RV, Blow FC, Hays RW, Cassidy KA, et al. Aripiprazole therapy in 20 older adults with bipolar disorder: a 12-week, open-label trial. J Clin Psychiatry. 2008;69(1):41-6.

320. San L, Arranz B, Perez V, Safont G, Corripio I, Ramirez N, et al. One-year, randomized, open trial comparing olanzapine, quetiapine, risperidone and ziprasidone effectiveness in antipsychotic-naive patients with a first-episode psychosis. Psychiatry Res. 2012;200(2-3):693-701.

321. Sanger TM, Grundy SL, Gibson PJ, Namjoshi MA, Greaney MG, Tohen MF. Long-term olanzapine therapy in the treatment of bipolar I disorder: an open-label continuation phase study. J Clin Psychiatry. 2001;62(4):273-81.

322. Sanger TM, Tohen M, Vieta E, Dunner DL, Bowden CL, Calabrese JR, et al. Olanzapine in the acute treatment of bipolar I disorder with a history of rapid cycling. J Affect Disord. 2003;73(1-2):155-61.

323. Sanz-Fuentenebro J, Taboada D, Palomo T, Aragues M, Ovejero S, Del Alamo C, et al. Randomized trial of clozapine vs. risperidone in treatment-naive first-episode schizophrenia: results after one year. Schizophr Res. 2013;149(1-3):156-61.

324. Schneider LS, Tariot PN, Dagerman KS, Davis SM, Hsiao JK, Ismail MS, et al. Effectiveness of atypical antipsychotic drugs in patients with Alzheimer's disease. N Engl J Med. 2006;355(15):1525-38.

325. Schoemaker J, Naber D, Vrijland P, Panagides J, Emsley R. Long-term assessment of Asenapine vs. Olanzapine in patients with schizophrenia or schizoaffective disorder. Pharmacopsychiatry. 2010;43(4):138-46.

326. Schoemaker J, Stet L, Vrijland P, Naber D, Panagides J, Emsley R. Long-term efficacy and safety of asenapine or olanzapine in patients with schizophrenia or schizoaffective disorder: an extension study. Pharmacopsychiatry. 2012;45(5):196-203.

327. Schooler N, Rabinowitz J, Davidson M, Emsley R, Harvey PD, Kopala L, et al. Risperidone and haloperidol in first-episode psychosis: a long-term randomized trial. Am J Psychiatry. 2005;162(5):947-53.

328. Schreiner A, Niehaus D, Shuriquie NA, Aadamsoo K, Korcsog P, Salinas R, et al. Metabolic effects of paliperidone extended release versus oral olanzapine in patients with schizophrenia: a prospective, randomized, controlled trial. J Clin Psychopharmacol. 2012;32(4):449-57.

329. Schreiner A, Bergmans P, Cherubin P, Keim S, Rancans E, Bez Y, et al. A prospective flexible-dose study of paliperidone palmitate in nonacute but symptomatic patients with schizophrenia previously unsuccessfully treated with oral antipsychotic agents. Clin Ther. 2014;36(10):1372-88 e1.

330. Schulz SC, Camlin KL, Berry SA, Jesberger JA. Olanzapine safety and efficacy in patients with borderline personality disorder and comorbid dysthymia. Biol Psychiatry. 1999;46(10):1429-35.

331. Schulz SC, Zanarini MC, Bateman A, Bohus M, Detke HC, Trzaskoma Q, et al. Olanzapine for the treatment of borderline personality disorder: variable dose 12-week randomised double-blind placebo-controlled study. Br J Psychiatry. 2008;193(6):485-92.

332. Sechter D, Peuskens J, Fleurot O, Rein W, Lecrubier Y. Amisulpride vs. risperidone in chronic schizophrenia: results of a 6-month double-blind study. Neuropsychopharmacology. 2002;27(6):1071-81.

333. Sengupta SM, Klink R, Stip E, Baptista T, Malla A, Joober R. Weight gain and lipid metabolic abnormalities induced by olanzapine in first-episode, drug-naive patients with psychotic disorders. Schizophr Res. 2005;80(1):131-3.

334. Sevy S, Robinson DG, Sunday S, Napolitano B, Miller R, McCormack J, et al. Olanzapine vs. risperidone in patients with first-episode schizophrenia and a lifetime history of cannabis use disorders: 16-week clinical and substance use outcomes. Psychiatry Res. 2011;188(3):310-4.

335. Si T, Zhang K, Tang J, Fang M, Li K, Zhuo J, et al. Efficacy and safety of flexibly dosed paliperidone palmitate in Chinese patients with acute schizophrenia: an open-label, single-arm, prospective, interventional study. Neuropsychiatr Dis Treat. 2015;11:1483-92.

336. Simpson MM, Goetz RR, Devlin MJ, Goetz SA, Walsh BT. Weight gain and antipsychotic medication: differences between antipsychotic-free and treatment periods. J Clin Psychiatry. 2001;62(9):694-700.

337. Simpson GM, Glick ID, Weiden PJ, Romano SJ, Siu CO. Randomized, controlled, double-blind multicenter comparison of the efficacy and tolerability of ziprasidone and olanzapine in acutely ill inpatients with schizophrenia or schizoaffective disorder. Am J Psychiatry. 2004;161(10):1837-47.

338. Singh A, Chandra S, Kapoor AK, Singh HK. Metabolic effects of Olanzapine versus Iloperidone: A 24 weeks randomized, prospective, interventional study. Internet Journal of Medical Update. 2016;11(2):17 - 24.

339. Smulevich AB, Khanna S, Eerdekens M, Karcher K, Kramer M, Grossman F. Acute and continuation risperidone monotherapy in bipolar mania: a 3-week placebo-controlled trial followed by a 9-week double-blind trial of risperidone and haloperidol. Eur Neuropsychopharmacol. 2005;15(1):75-84.

340. Sobis J, Rykaczewska-Czerwinska M, Swietochowska E, Gorczyca P. Therapeutic effect of aripiprazole in chronic schizophrenia is accompanied by anti-inflammatory activity. Pharmacol Rep. 2015;67(2):353-9.

341. Soler J, Pascual JC, Campins J, Barrachina J, Puigdemont D, Alvarez E, et al. Double-blind, placebo-controlled study of dialectical behavior therapy plus olanzapine for borderline personality disorder. Am J Psychiatry. 2005;162(6):1221-4.

342. Song X, Fan X, Li X, Zhang W, Gao J, Zhao J, et al. Changes in pro-inflammatory cytokines and body weight during 6-month risperidone treatment in drug naive, first-episode schizophrenia. Psychopharmacology (Berl). 2014;231(2):319-25.

343. Sowell MO, Mukhopadhyay N, Cavazzoni P, Shankar S, Steinberg HO, Breier A, et al. Hyperglycemic clamp assessment of insulin secretory responses in normal subjects treated with olanzapine, risperidone, or placebo. J Clin Endocrinol Metab. 2002;87(6):2918-23.

344. Spivak B, Musin E, Mester R, Gonen N, Talmon Y, Guy N, et al. The effect of long-term antipsychotic treatment on the body weight of patients suffering from chronic schizophrenia: clozapine versus classical antipsychotic agents. Int Clin Psychopharmacol. 1999;14(4):229-32.

345. Stip E, Lungu OV, Anselmo K, Letourneau G, Mendrek A, Stip B, et al. Neural changes associated with appetite information processing in schizophrenic patients after 16 weeks of olanzapine treatment. Transl Psychiatry. 2012;2:e128.

346. Strassnig M, Miewald J, Keshavan M, Ganguli R. Weight gain in newly diagnosed first-episode psychosis patients and healthy comparisons: one-year analysis. Schizophr Res. 2007;93(1-3):90-8.

347. Street JS, Clark WS, Kadam DL, Mitan SJ, Juliar BE, Feldman PD, et al. Long-term efficacy of olanzapine in the control of psychotic and behavioral symptoms in nursing home patients with Alzheimer's dementia. Int J Geriatr Psychiatry. 2001;16 Suppl 1:S62-70.

348. Stroup TS, McEvoy JP, Ring KD, Hamer RH, LaVange LM, Swartz MS, et al. A randomized trial examining the effectiveness of switching from olanzapine, quetiapine, or risperidone to aripiprazole to reduce metabolic risk: comparison of antipsychotics for metabolic problems (CAMP). Am J Psychiatry. 2011;168(9):947-56.

349. Strous RD, Kupchik M, Roitman S, Schwartz S, Gonen N, Mester R, et al. Comparison between risperidone, olanzapine, and clozapine in the management of chronic schizophrenia: a naturalistic prospective 12-week observational study. Hum Psychopharmacol. 2006;21(4):235-43.

350. Suppes T, Datto C, Minkwitz M, Nordenhem A, Walker C, Darko D. Effectiveness of the extended release formulation of quetiapine as monotherapy for the treatment of acute bipolar depression. J Affect Disord. 2010;121(1-2):106-15.

351. Suppes T, McElroy SL, Sheehan DV, Hidalgo RB, Cosgrove VE, Gwizdowski IS, et al. A randomized, double-blind, placebo-controlled study of ziprasidone monotherapy in bipolar disorder with co-occurring lifetime panic or generalized anxiety disorder. J Clin Psychiatry. 2014;75(1):77-84.

352. Suresh Kumar PN, Anish PK, Rajmohan V. Olanzapine has better efficacy compared to risperidone for treatment of negative symptoms in schizophrenia. Indian Journal of Psychiatry. 2016;58(3):311-6.

353. Suzuki H, Inoue Y, Gen K. A study of the efficacy and safety of switching from oral risperidone to risperidone long-acting injection in older patients with schizophrenia. Therapeutic Advanced in Psychopharmacology. 2012;2(6):227 - 34.

354. Takahashi H, Oshimo T, Ishigooka J. Efficacy and tolerability of aripiprazole in first-episode drug-naive patients with schizophrenia: an open-label trial. Clin Neuropharmacol. 2009;32(3):149-50.

355. Takeuchi H, Uchida H, Suzuki T, Watanabe K, Kashima H. Changes in metabolic parameters following a switch to aripiprazole in Japanese patients with schizophrenia: One-year follow-up study. Psychiatry Clin Neurosci. 2010;64(1):104-6.

356. Tamayo JM, Mazzotti G, Tohen M, Gattaz WF, Zapata R, Castillo JJ, et al. Outcomes for Latin American versus White patients suffering from acute mania in a randomized, double-blind trial comparing olanzapine and haloperidol. J Clin Psychopharmacol. 2007;27(2):126-34.

357. Tandon R, Cucchiaro J, Phillips D, Hernandez D, Mao Y, Pikalov A, et al. A double-blind, placebo-controlled, randomized withdrawal study of lurasidone for the maintenance of efficacy in patients with schizophrenia. J Psychopharmacol. 2016;30(1):69-77.

358. Tariot PN, Salzman C, Yeung PP, Pultz J, Rak IW. Long-Term use of quetiapine in elderly patients with psychotic disorders. Clin Ther. 2000;22(9):1068-84.

359. Tauscher-Wisniewski S, Kapur S, Tauscher J, Jones C, Daskalakis ZJ, Papatheodorou G, et al. Quetiapine: an effective antipsychotic in first-episode schizophrenia despite only transiently high dopamine-2 receptor blockade. J Clin Psychiatry. 2002;63(11):992-7.

360. Teff KL, Rickels MR, Grudzial J, Fuller C, Nguyen H.L., Rickels K. Antipsychotic-Induced Insulin Resistance and Postprandial Hormonal Dysregulation Independent of Weight Gain or Psychiatric Disease. Diabetes. 2013;62:3232 - 40.

361. Thase ME, Jonas A, Khan A, Bowden CL, Wu X, McQuade RD, et al. Aripiprazole monotherapy in nonpsychotic bipolar I depression: results of 2 randomized, placebo-controlled studies. J Clin Psychopharmacol. 2008;28(1):13-20.

362. Thomas P, Srivastava V, Singh A, Mathur P, Nimgaonkar VL, Lerer B, et al. Correlates of response to Olanzapine in a North Indian Schizophrenia sample. Psychiatry Res. 2008;161(3):275-83.

363. Tohen M, Jacobs TG, Grundy SL, McElroy SL, Banov MC, Janicak PG, et al. Efficacy of olanzapine in acute bipolar mania: a double-blind, placebo-controlled study. The Olanzipine HGGW Study Group. Arch Gen Psychiatry. 2000;57(9):841-9.

364. Tohen M, Goldberg JF, Gonzalez-Pinto Arrillaga AM, Azorin JM, Vieta E, Hardy-Bayle MC, et al. A 12-week, double-blind comparison of olanzapine vs haloperidol in the treatment of acute mania. Arch Gen Psychiatry. 2003;60(12):1218-26.

365. Tohen M, Calabrese JR, Sachs GS, Banov MD, Detke HC, Risser R, et al. Randomized, placebo-controlled trial of olanzapine as maintenance therapy in patients with bipolar I disorder responding to acute treatment with olanzapine. Am J Psychiatry. 2006;163(2):247-56.

366. Tohen M, McDonnell DP, Case M, Kanba S, Ha K, Fang YR, et al. Randomised, double-blind, placebo-controlled study of olanzapine in patients with bipolar I depression. Br J Psychiatry. 2012;201(5):376-82.

367. Tollefson GD, Beasley CM, Jr., Tran PV, Street JS, Krueger JA, Tamura RN, et al. Olanzapine versus haloperidol in the treatment of schizophrenia and schizoaffective and schizophreniform disorders: results of an international collaborative trial. Am J Psychiatry. 1997;154(4):457-65.

368. Tollefson GD, Birkett MA, Kiesler GM, Wood AJ. Double-blind comparison of olanzapine versus clozapine in schizophrenic patients clinically eligible for treatment with clozapine. Biol Psychiatry. 2001;49(1):52-63.

369. Tran PV, Tollefson GD, Sanger TM, Lu Y, Berg PH, Beasley CM, Jr. Olanzapine versus haloperidol in the treatment of schizoaffective disorder. Acute and long-term therapy. Br J Psychiatry. 1999;174:15-22.

370. Tsai SJ, Yu YW, Lin CH, Wang YC, Chen JY, Hong CJ. Association study of adrenergic beta3 receptor (Trp64Arg) and G-protein beta3 subunit gene (C825T) polymorphisms and weight change during clozapine treatment. Neuropsychobiology. 2004;50(1):37-40.

371. Tybura P, Trzesniowska-Drukala B, Bienkowski P, Beszlej A, Frydecka D, Mierzejewski P, et al. Pharmacogenetics of adverse events in schizophrenia treatment: comparison study of ziprasidone, olanzapine and perazine. Psychiatry Res. 2014;219(2):261-7.

372. Tzimos A, Samokhvalov V, Kramer M, Ford L, Gassmann-Mayer C, Lim P, et al. Safety and tolerability of oral paliperidone extended-release tablets in elderly patients with schizophrenia: a double-blind, placebo-controlled study with six-month open-label extension. Am J Geriatr Psychiatry. 2008;16(1):31-43.

373. Ucok A, Saka MC, Bilici M. Effects of paliperidone extended release on

functioning level and symptoms of patients

with recent onset schizophrenia:

An open-label, single-arm, fl exible-dose,

12-months follow-up study. Nordic Journal of Psychiatry. 2015;69:426 - 32.

374. Umbricht DS, Pollack S, Kane JM. Clozapine and weight gain. J Clin Psychiatry. 1994;55 Suppl B:157-60.

375. Van Ameringen M, Mancini C, Patterson B, Bennett M, Oakman J. A randomized, double-blind, placebo-controlled trial of olanzapine in the treatment of trichotillomania. J Clin Psychiatry. 2010;71(10):1336-43.

376. van Bruggen J, Tijssen J, Dingemans KP, Gersons B, Linszen D. Symptom response and side-effects of olanzapine and risperidone in young adults with recent onset schizophrenia. International Clinical Psychopharmacology. 2003;18:341 - 6.

377. Vazquez-Bourgon J, Perez-Iglesias R, Ortiz-Garcia de la Foz V, Suarez Pinilla P, Diaz Martinez A, Crespo-Facorro B. Long-term metabolic effects of aripiprazole, ziprasidone and quetiapine: a pragmatic clinical trial in drug-naive patients with a first-episode of non-affective psychosis. Psychopharmacology (Berl). 2018;235(1):245-55.

378. Vieta E, Nuamah IF, Lim P, Yuen EC, Palumbo JM, Hough DW, et al. A randomized, placebo- and active-controlled study of paliperidone extended release for the treatment of acute manic and mixed episodes of bipolar I disorder. Bipolar Disord. 2010;12(3):230-43.

379. Villarreal G, Calais LA, Canive JM, Lundy SL, Pickard J, Toney G. Prospective study to evaluate the efficacy of aripiprazole as a monotherapy in patients with severe chronic posttraumatic stress disorder: an open trial. Psychopharmacol Bull. 2007;40(2):6-18.

380. Villarreal G, Hamner MB, Canive JM, Robert S, Calais LA, Durklaski V, et al. Efficacy of Quetiapine Monotherapy in Posttraumatic Stress Disorder: A Randomized, Placebo-Controlled Trial. Am J Psychiatry. 2016;173(12):1205-12.

381. Villeneuve E, Lemelin S. Open-label study of atypical neuroleptic quetiapine for treatment of borderline personality disorder: impulsivity as main target. J Clin Psychiatry. 2005;66(10):1298-303.

382. Waitzkin L. Glucose tolerance in man during chlorpromazine therapy. Diabetes. 1970;19(3):186-8.

383. Wampers M, Hanssens L, van Winkel R, Heald A, Collette J, Peuskens J, et al. Differential effects of olanzapine and risperidone on plasma adiponectin levels over time: results from a 3-month prospective open-label study. Eur Neuropsychopharmacol. 2012;22(1):17-26.

384. Wang F, Mi W, Ma W, Ma C, Yang Y, Zhang H, et al. A pharmacogenomic study revealed an association between SLC6A4 and risperidone-induced weight gain in Chinese Han population. Pharmacogenomics. 2015;16(17):1943-9.

385. Wang X, Savage R, Borisov A, Rosenberg J, Woolwine B, Tucker M, et al. Efficacy of risperidone versus olanzapine in patients with schizophrenia previously on chronic conventional antipsychotic therapy: a switch study. J Psychiatr Res. 2006;40(7):669-76.

386. Wang M, Tong JH, Huang DS, Zhu G, Liang GM, Du H. Efficacy of olanzapine monotherapy for treatment of bipolar I depression: a randomized, double-blind, placebo controlled study. Psychopharmacology (Berl). 2014;231:2811 - 8.

387. Wang HH, Cai M, Wang HN, Chen YC, Zhang RG, Wang Y, et al. An assessor-blinded, randomized comparison of efficacy and tolerability of switching from olanzapine to ziprasidone and the combination of both in schizophrenia spectrum disorders. J Psychiatr Res. 2017;85:59-65.

388. Weiden PJ, Simpson GM, Potkin SG, O'Sullivan RL. Effectiveness of switching to ziprasidone for stable but symptomatic outpatients with schizophrenia. J Clin Psychiatry. 2003;64(5):580-8.

389. Weiden PJ, Citrome L, Alva G, Brams M, Glick ID, Jackson R, et al. A trial evaluating gradual- or immediate-switch strategies from risperidone, olanzapine, or aripiprazole to iloperidone in patients with schizophrenia. Schizophr Res. 2014;153(1-3):160-8.

390. White MP, Koran LM. Open-label trial of aripiprazole in the treatment of trichotillomania. J Clin Psychopharmacol. 2011;31(4):503-6.

391. Wirshing DA, Wirshing WC, Kysar L, Berisford MA, Goldstein D, Pashdag J, et al. Novel antipsychotics: comparison of weight gain liabilities. J Clin Psychiatry. 1999;60(6):358-63.

392. Woo YS, Bahk WM, Park YM, Chung S, Yoon BH, Won S, et al. Effects of switching to aripiprazole from current atypical antipsychotics on subsyndromal symptoms and tolerability in patients with bipolar disorder. Int Clin Psychopharmacol. 2016;31(5):275-86.

393. Yang J, Bahk WM, Cho HS, Jeon YW, Jon DI, Jung HY, et al. Efficacy and tolerability of Blonanserin in the patients with schizophrenia: a randomized, double-blind, risperidone-compared trial. Clin Neuropharmacol. 2010;33(4):169-75.

394. Yang F, Chen L, Fang X, Zheng K, Zhu C, Xu C, et al. Influence of olanzapine on serum prolactin levels and BMI in female patients with schizophrenia. Neuropsychiatr Dis Treat. 2018;14:3373-9.

395. Yap HL, Mahendran R, Lim D, Liow PH, Lee A, Phang S, et al. Risperidone in the treatment of first episode psychosis. Singapore Med J. 2001;42(4):170-3.

396. Young AH, Oren DA, Lowy A, McQuade RD, Marcus RN, Carson WH, et al. Aripiprazole monotherapy in acute mania: 12-week randomised placebo- and haloperidol-controlled study. Br J Psychiatry. 2009;194(1):40-8.

397. Youssef NA, Marx CE, Bradford DW, Zinn S, Hertzberg MA, Kilts JD, et al. An open-label pilot study of aripiprazole for male and female veterans with chronic post-traumatic stress disorder who respond suboptimally to antidepressants. Int Clin Psychopharmacol. 2012;27(4):191-6.

398. Zahan T, Akter N, Mullick MSI, Dewan ZF. Metabolic risk factor profile in patients on treatment with second generation antipsychotics. Bangladesh Med Res Counc Bull. 2015;41:144 - 50.

399. Zanarini MC, Frankenburg FR. Olanzapine treatment of female borderline personality disorder patients: a double-blind, placebo-controlled pilot study. J Clin Psychiatry. 2001;62(11):849-54.

400. Zanarini MC, Schulz SC, Detke HC, Tanaka Y, Zhao F, Lin D, et al. A dose comparison of olanzapine for the treatment of borderline personality disorder: a 12-week randomized, double-blind, placebo-controlled study. J Clin Psychiatry. 2011;72(10):1353-62.

401. Zhang Y, Dai G. Ef fi cacy and metabolic influence of paliperidone ER, aripiprazole and ziprasidone to patients with first-episode schizophrenia through 52 weeks follow-up in China. Hum Psychopharmacol. 2012;27(6):605-14.

402. Zhao T, Park TW, Yang JC, Huang GB, Kim MG, Lee KH, et al. Efficacy and safety of ziprasidone in the treatment of first-episode psychosis: an 8-week, open-label, multicenter trial. Int Clin Psychopharmacol. 2012;27(4):184-90.

403. Zhong KX, Sweitzer DE, Hamer RM, Lieberman JA. Comparison of quetiapine and risperidone in the treatment of schizophrenia: A randomized, double-blind, flexible-dose, 8-week study. J Clin Psychiatry. 2006;67(7):1093-103.

404. Zipursky RB, Gu H, Green AI, Perkins DO, Tohen MF, McEvoy JP, et al. Course and predictors of weight gain in people with first-episode psychosis treated with olanzapine or haloperidol. Br J Psychiatry. 2005;187:537-43.

S4 Forest plots per antipsychotic per duration of antipsychotic use

Figures S4a Forest plots AP Naïve studies on weight change


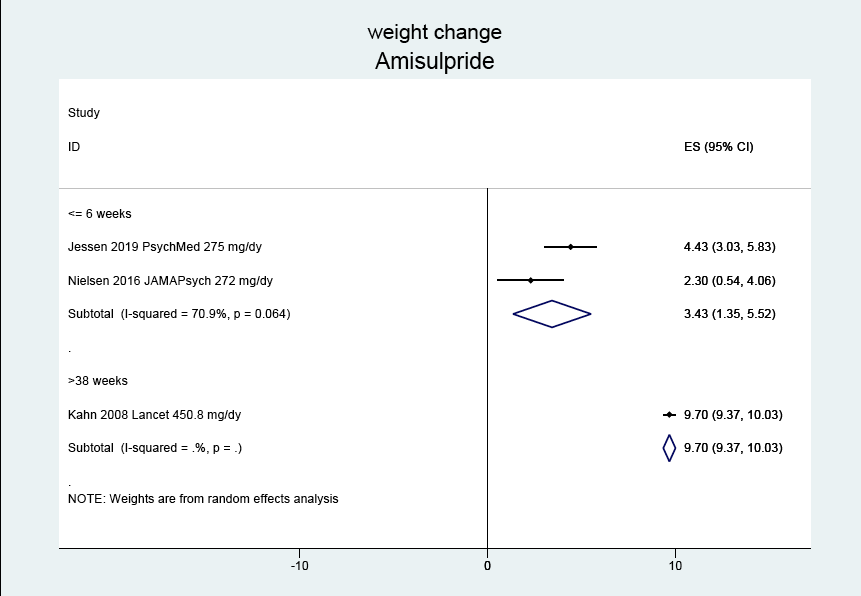


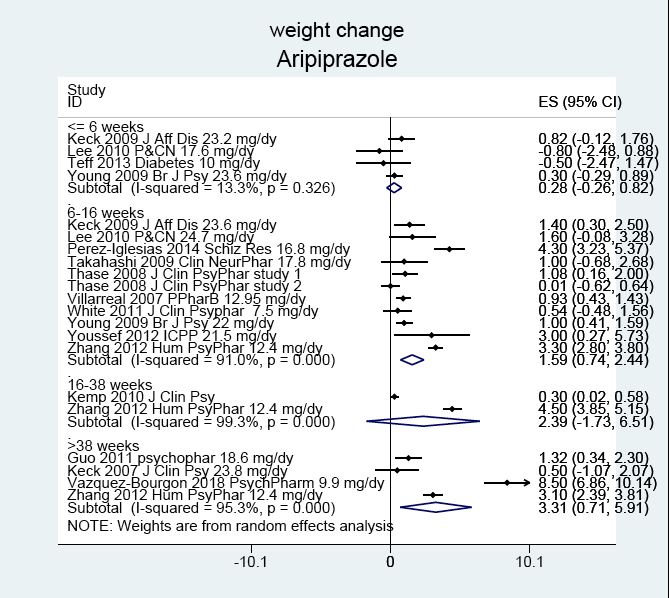


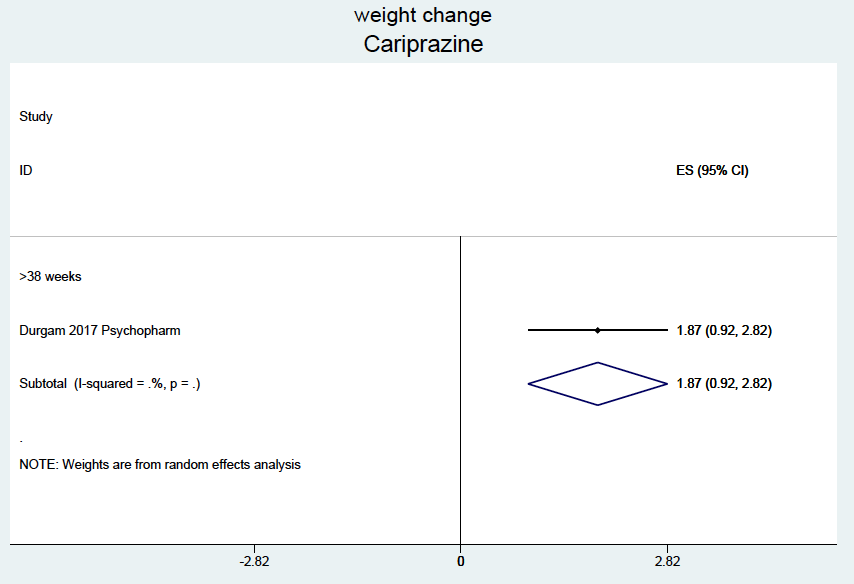


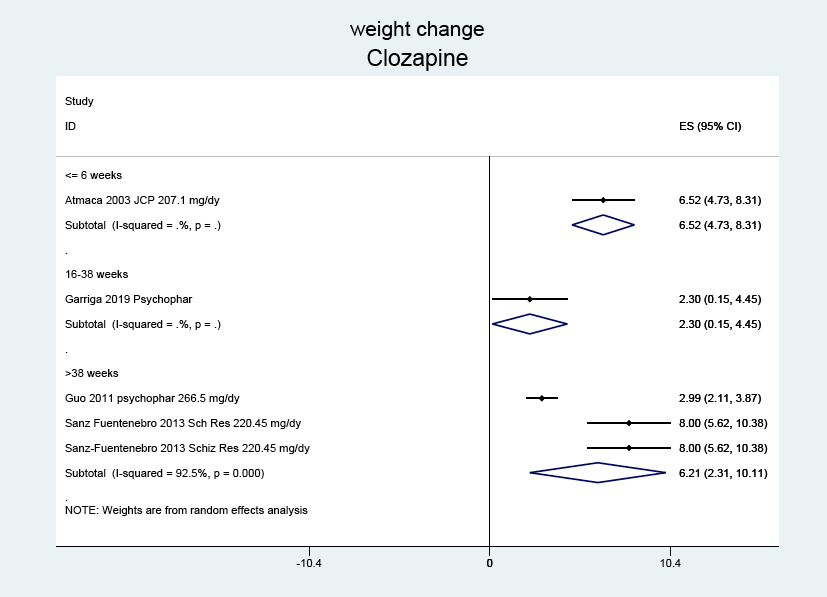


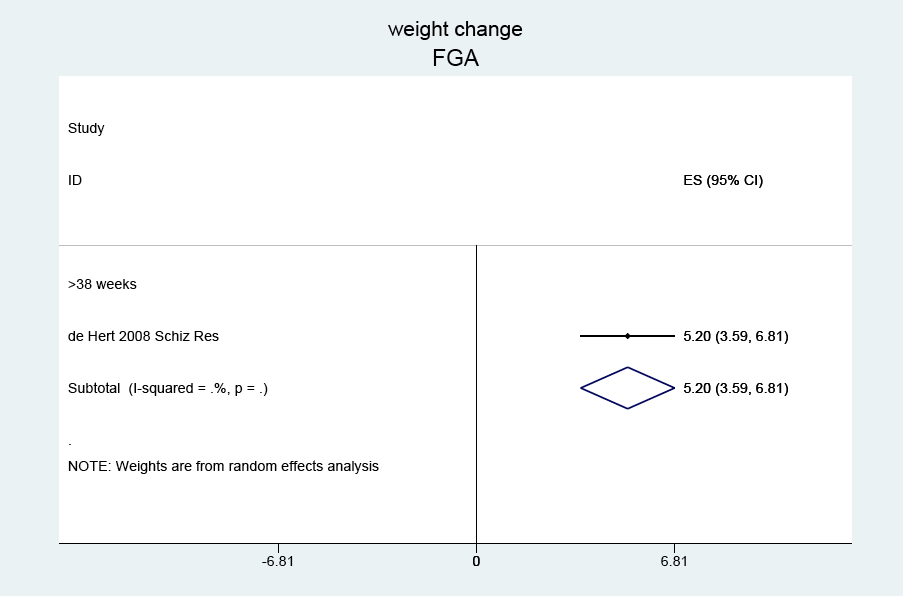


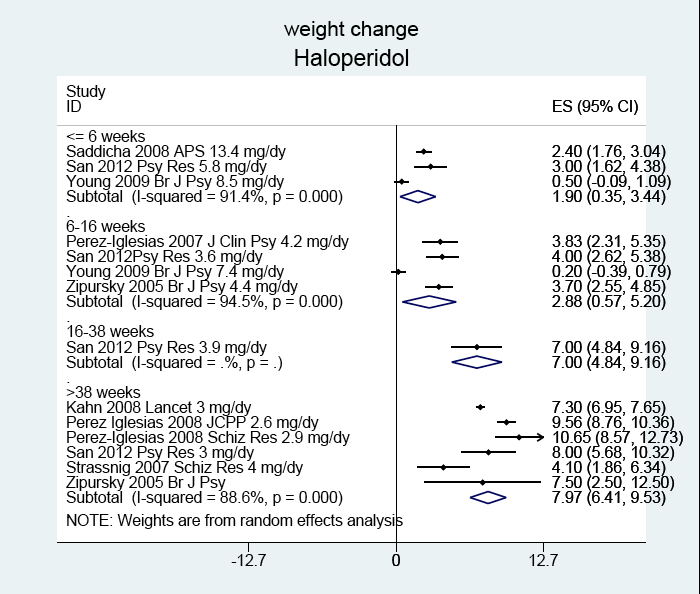


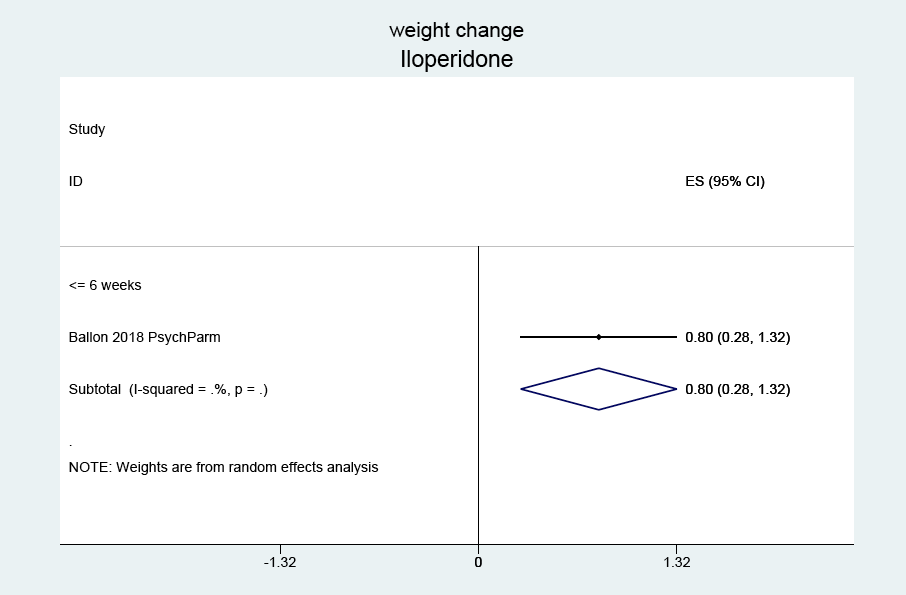


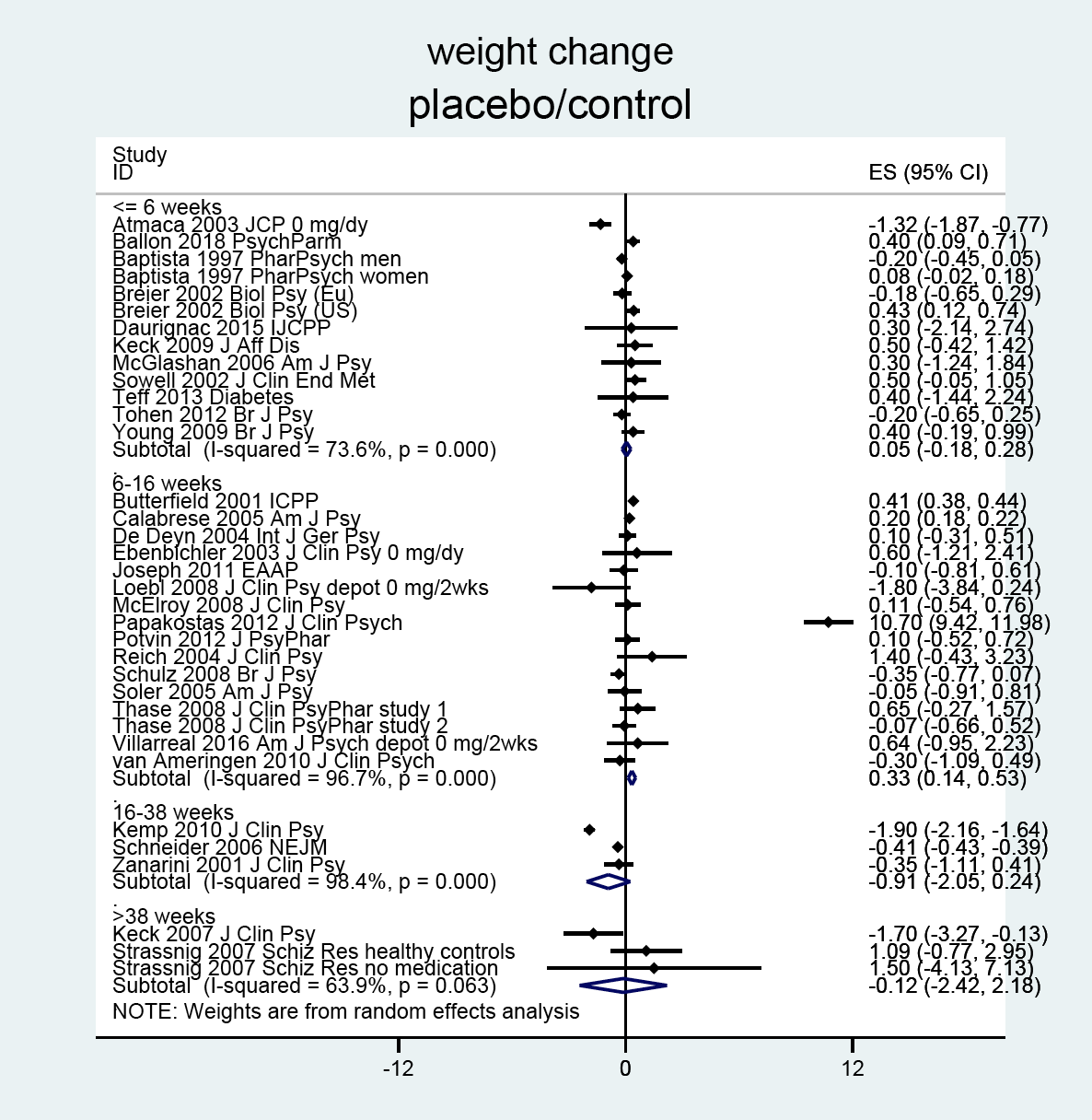


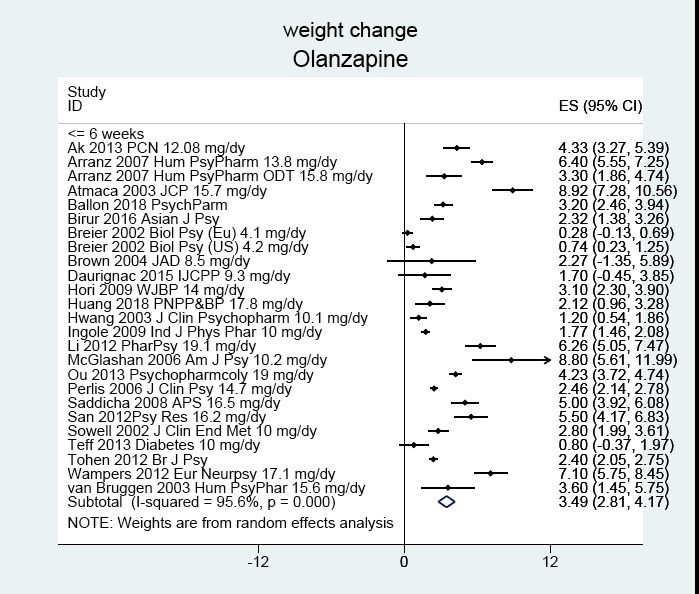


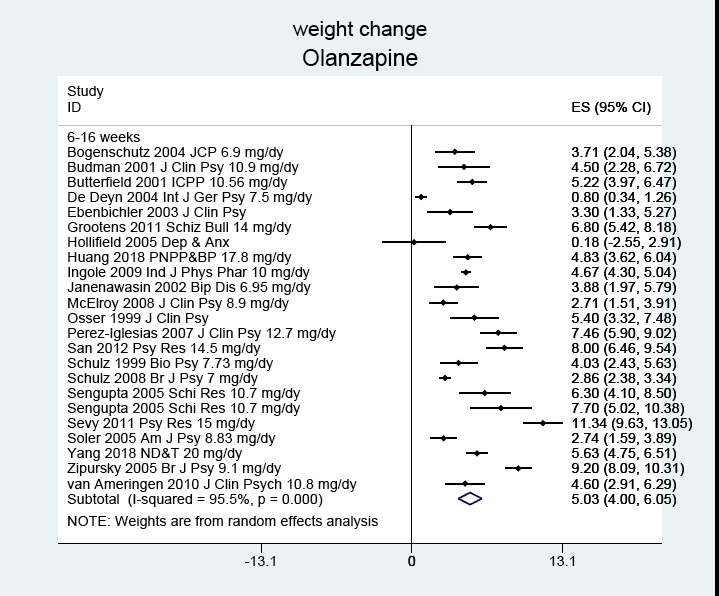


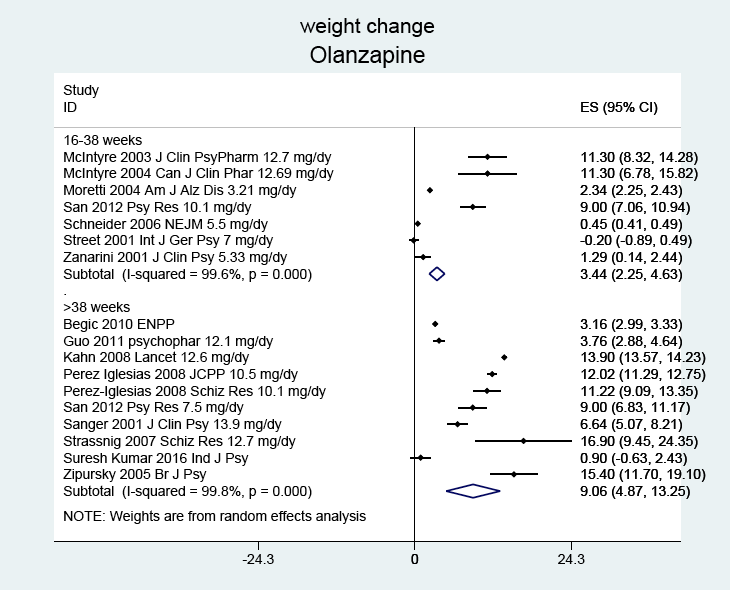


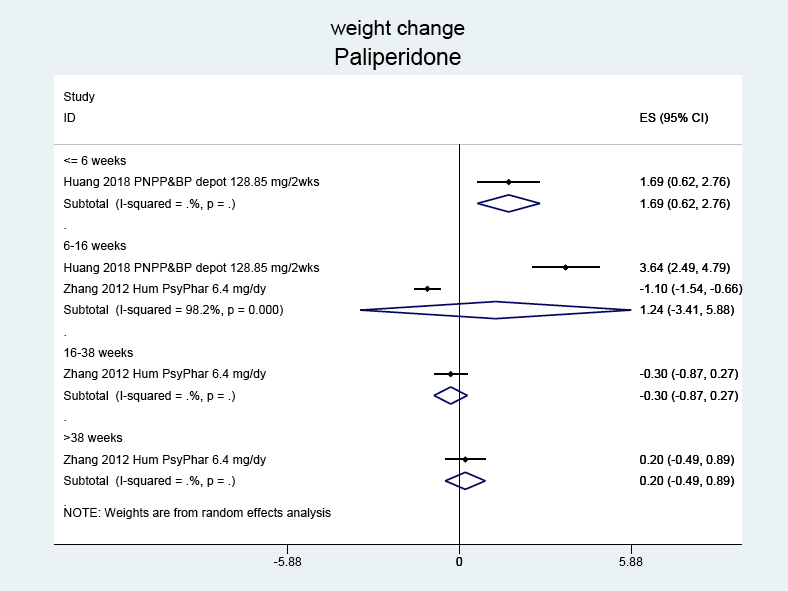


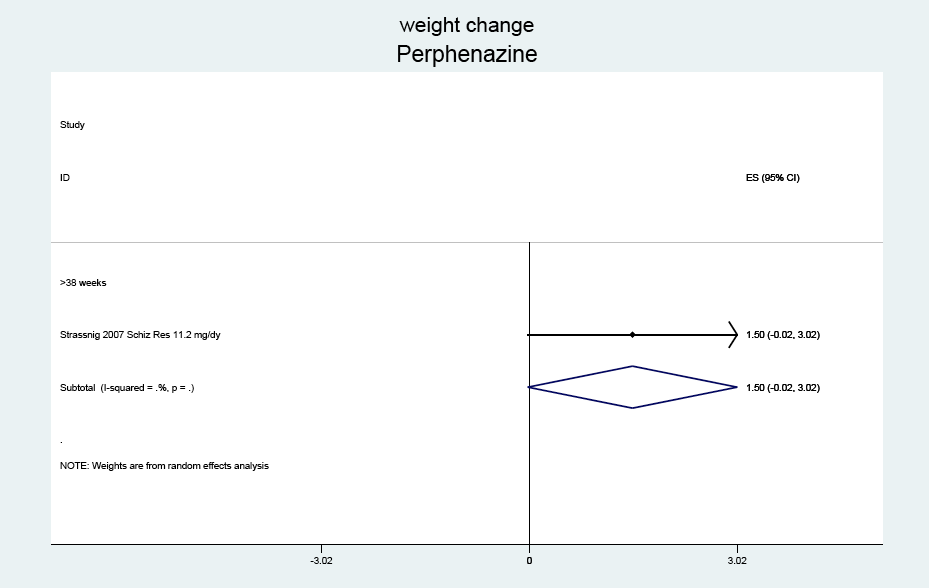


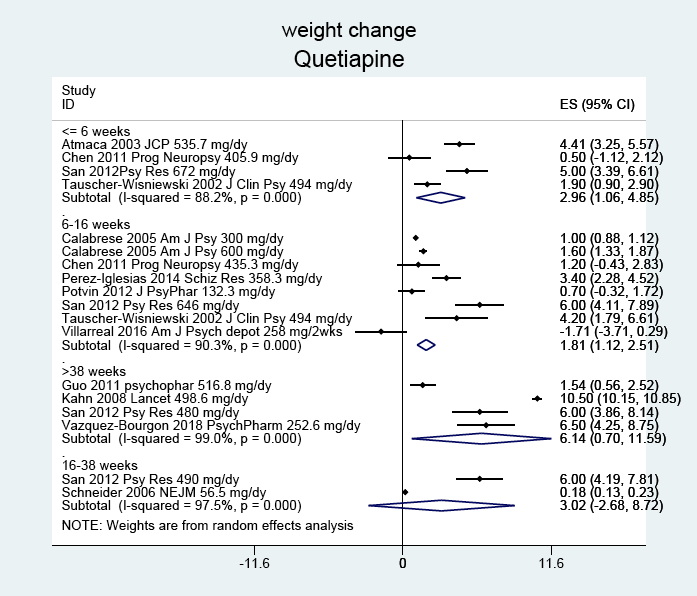


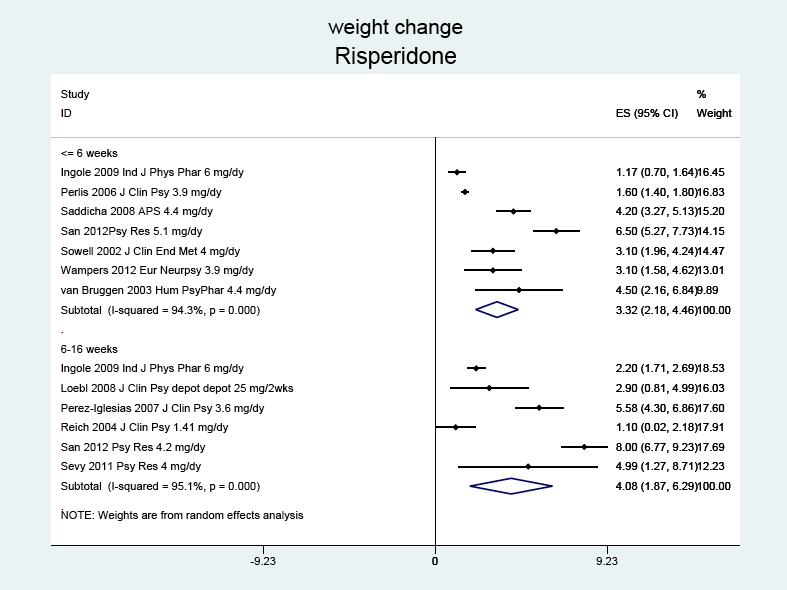


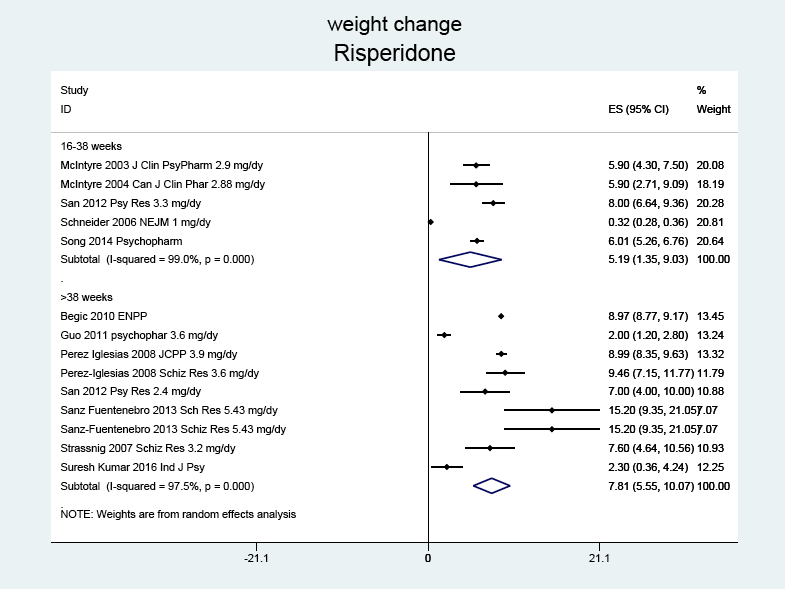


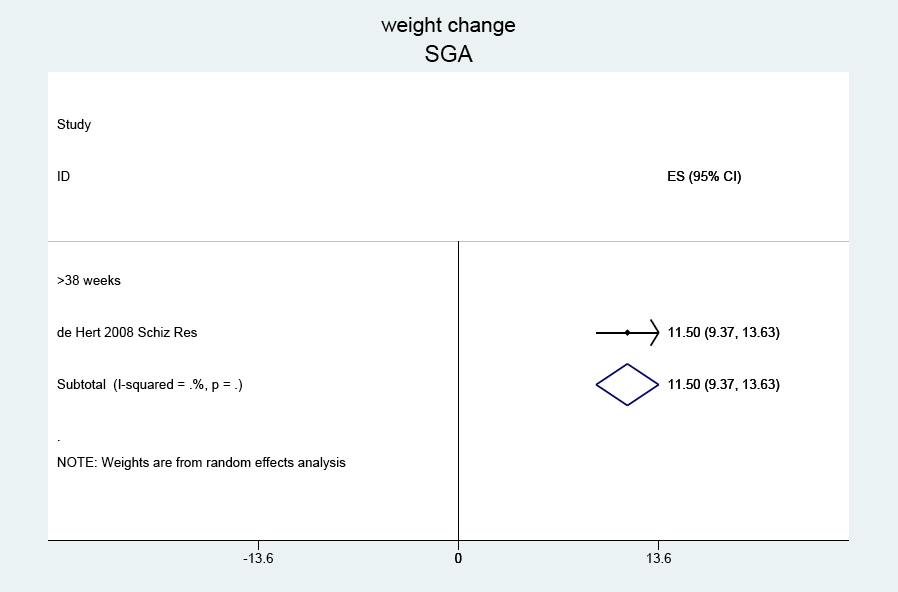


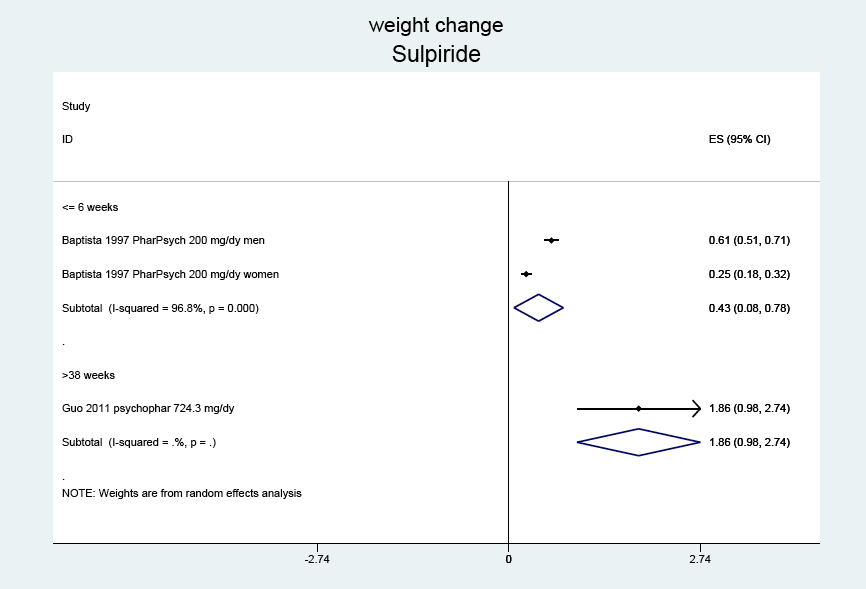


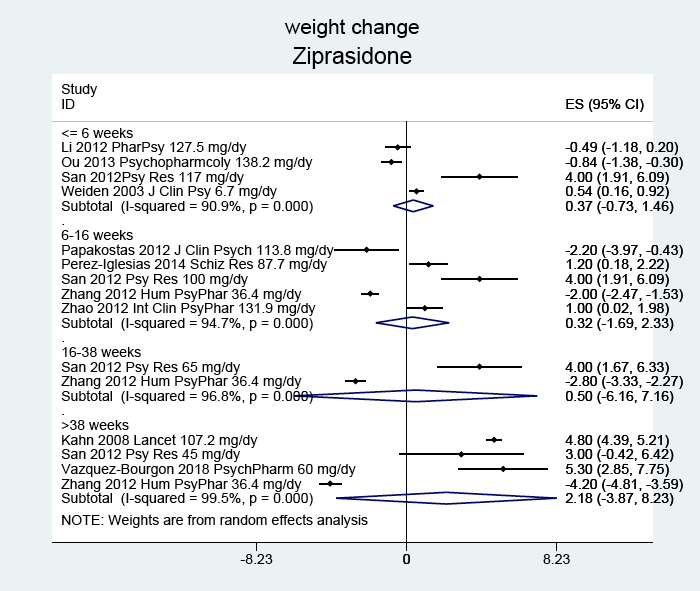


Figures 4b: Forest plots of AP-switch on weight change


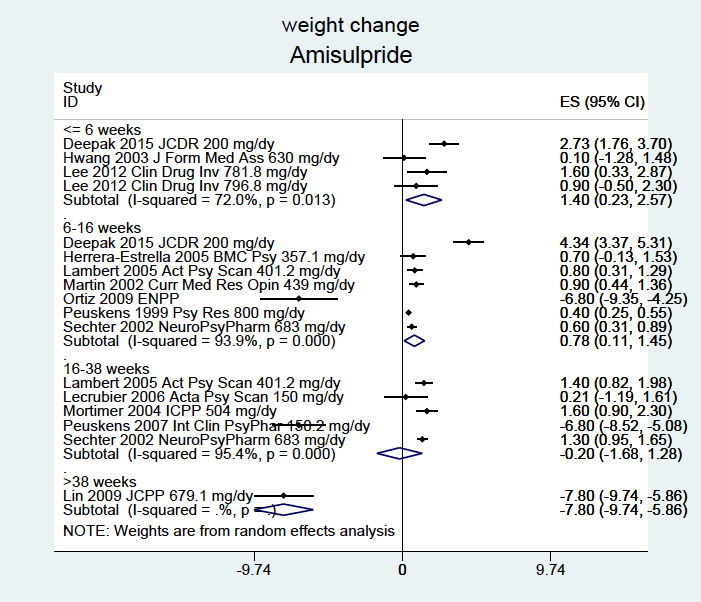


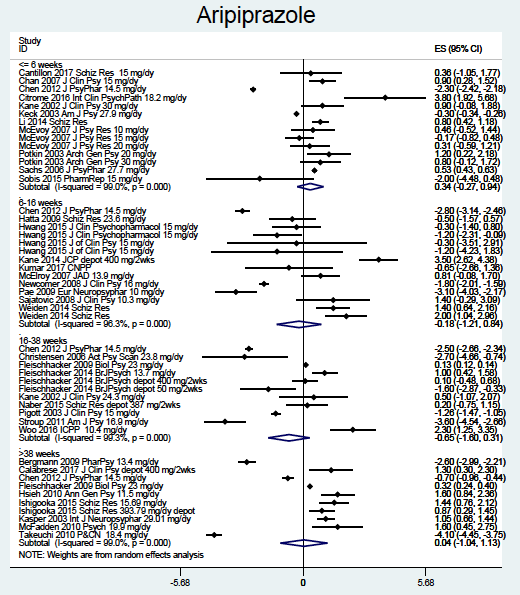


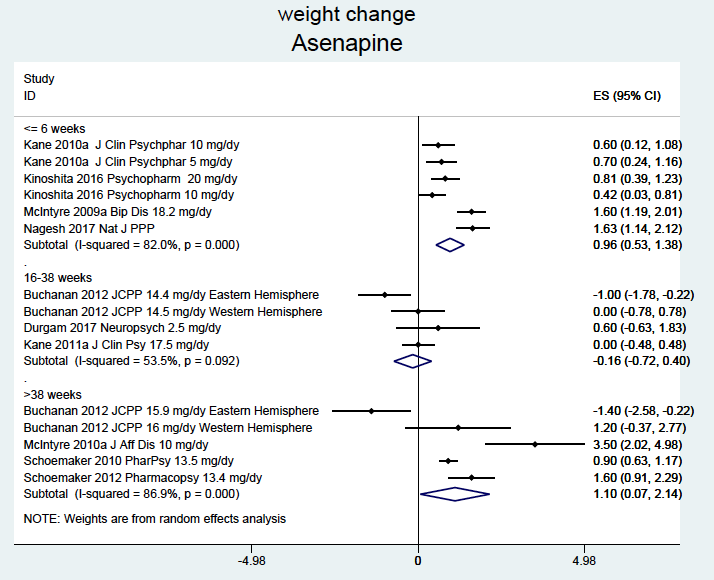


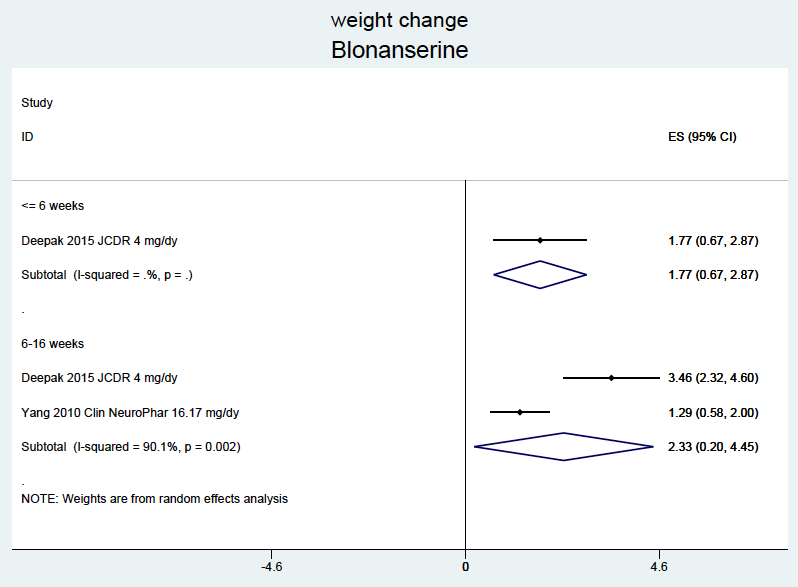


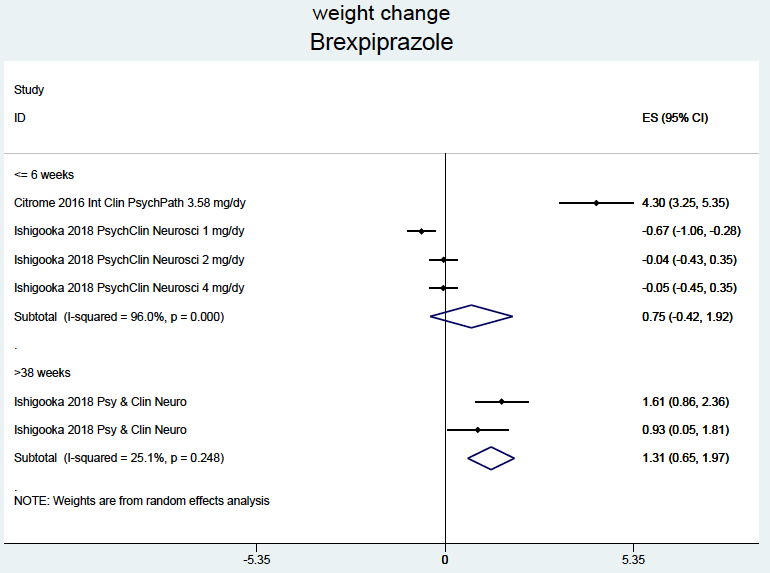


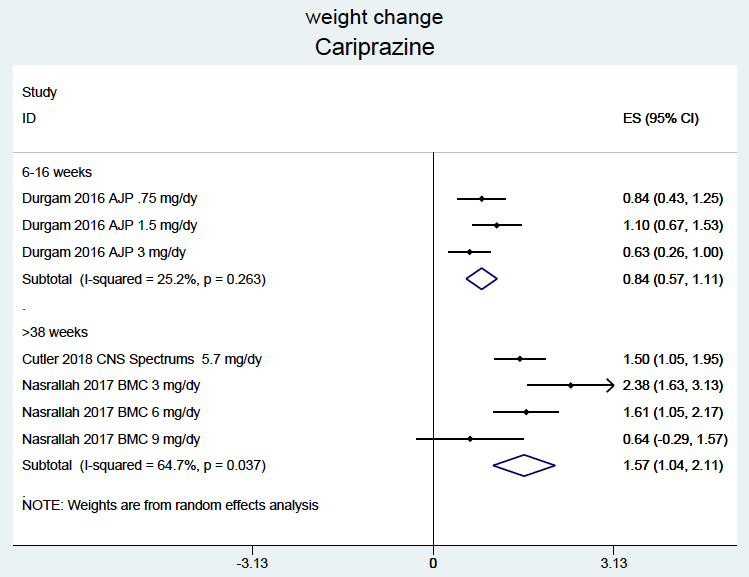


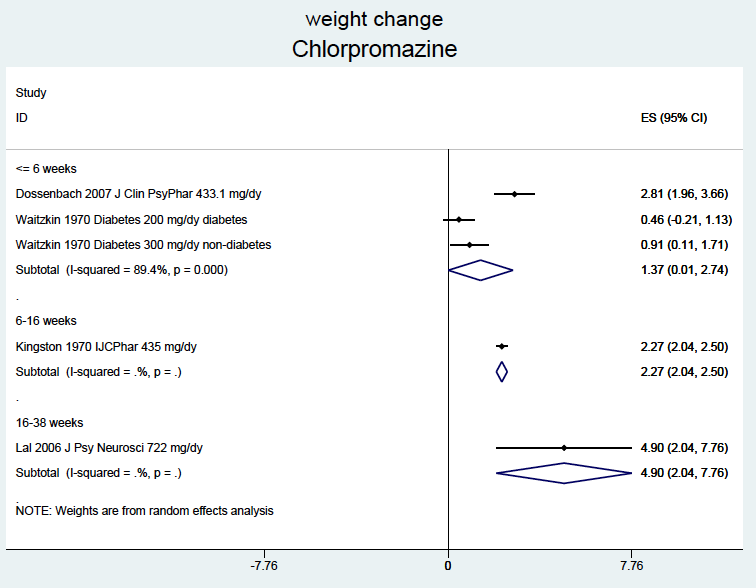


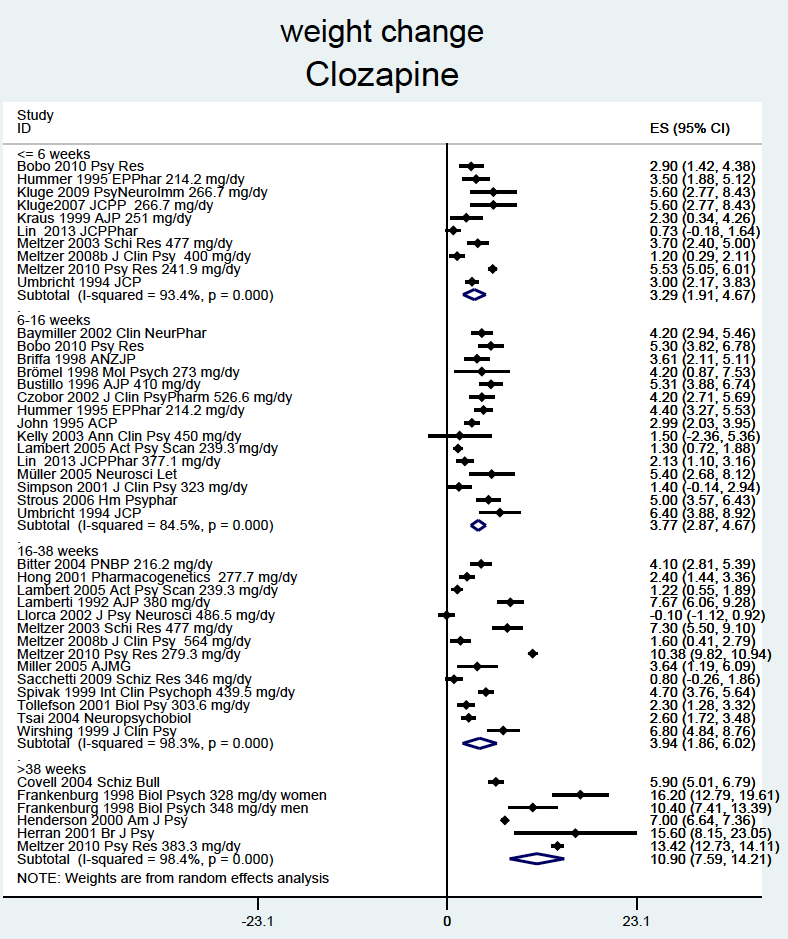


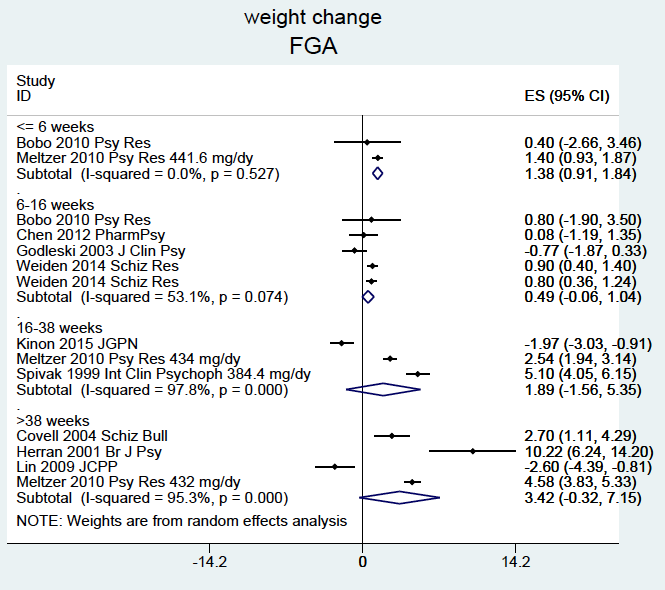


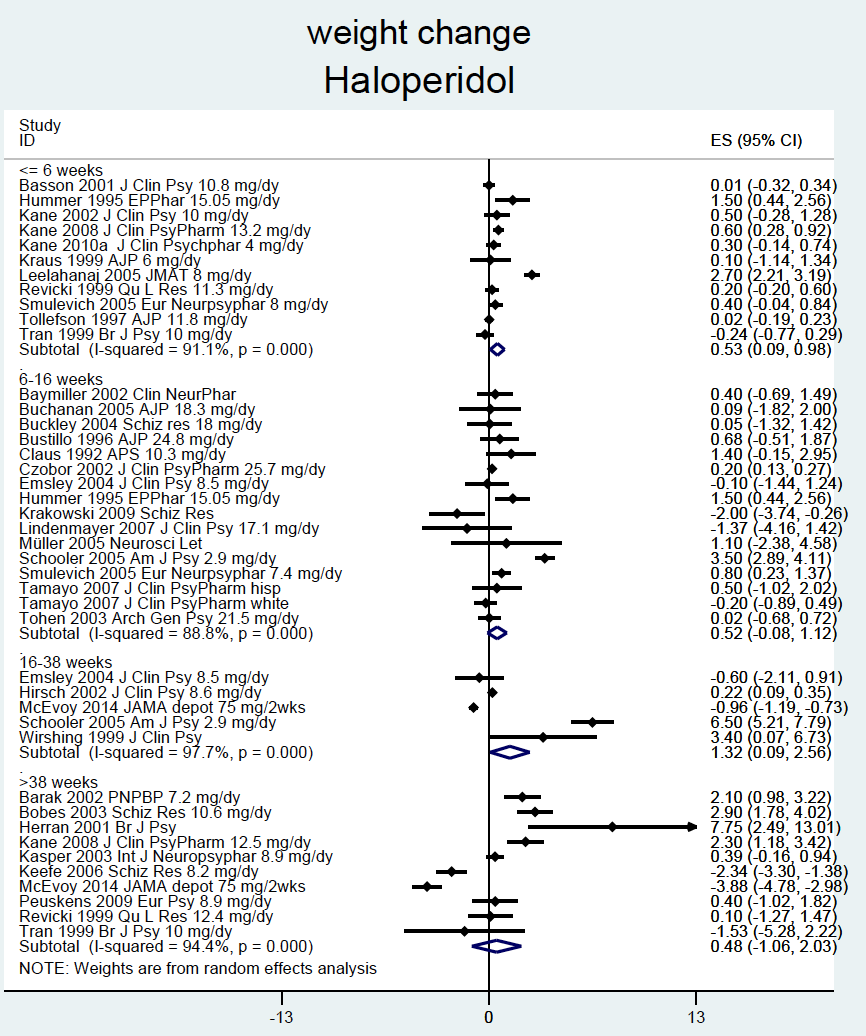


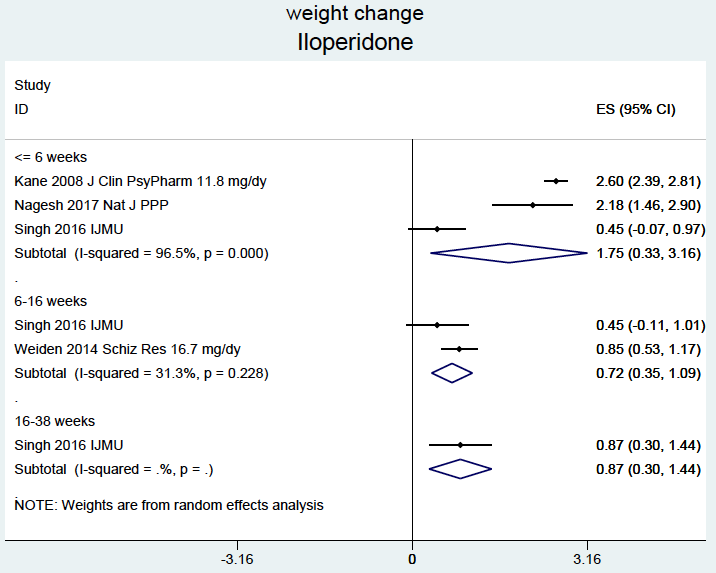


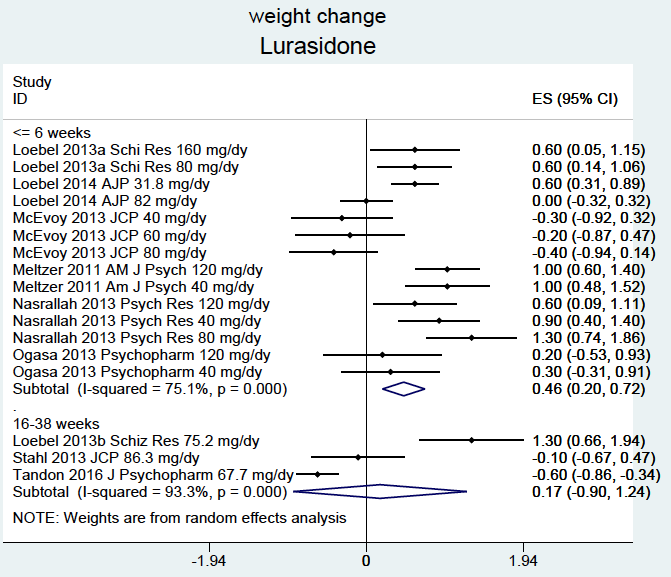


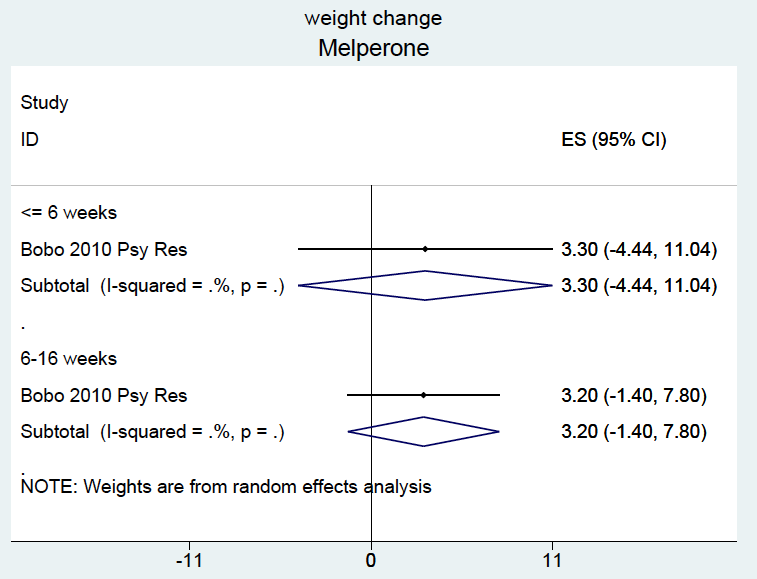


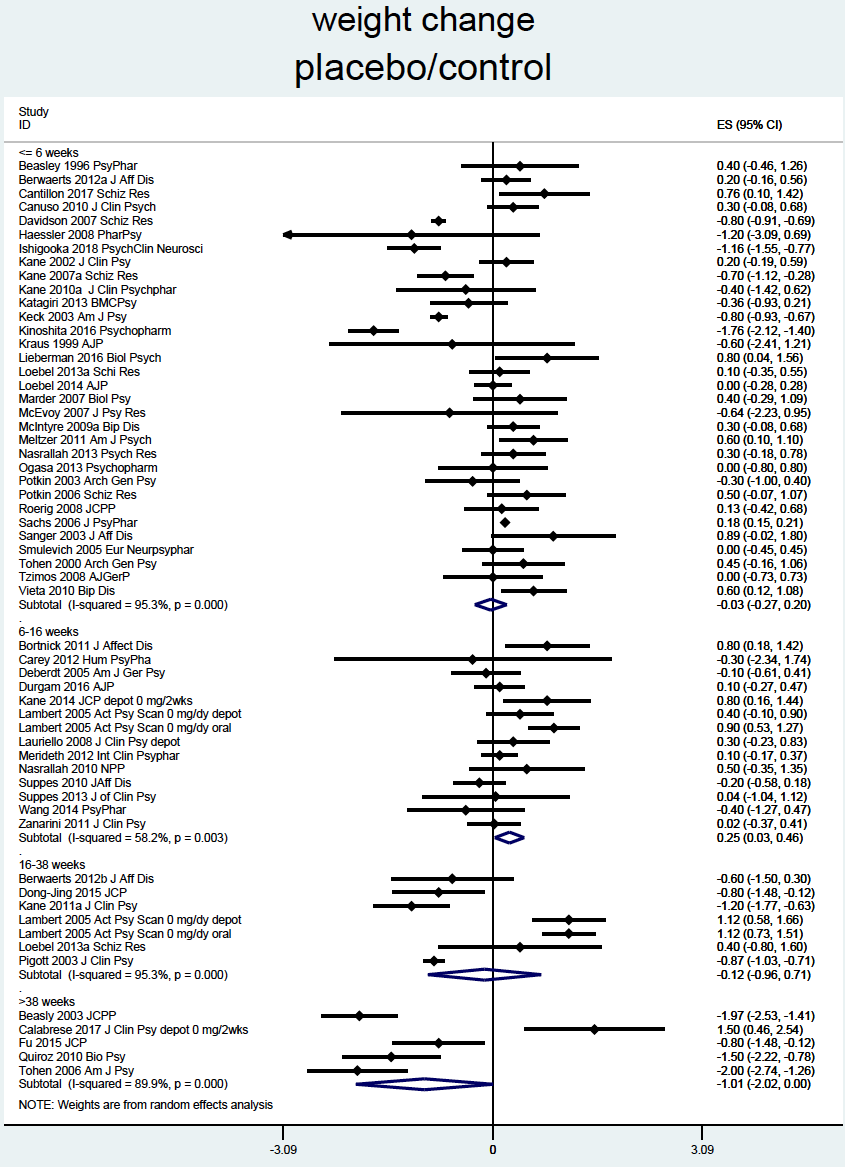


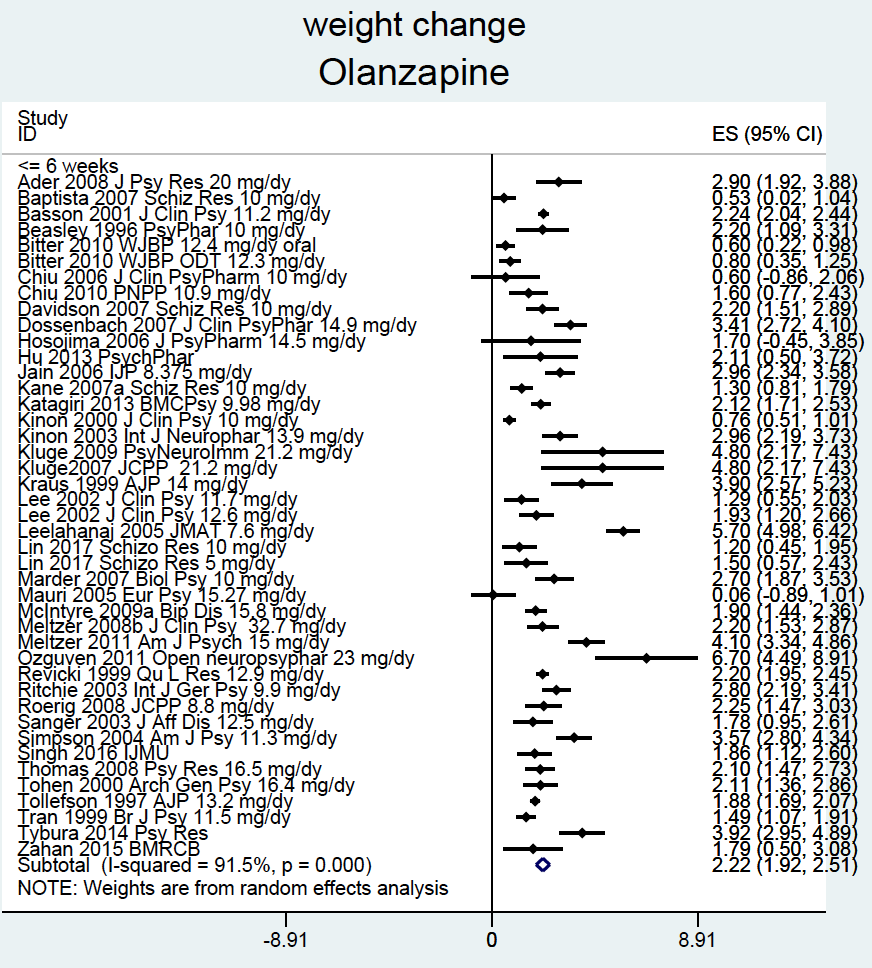


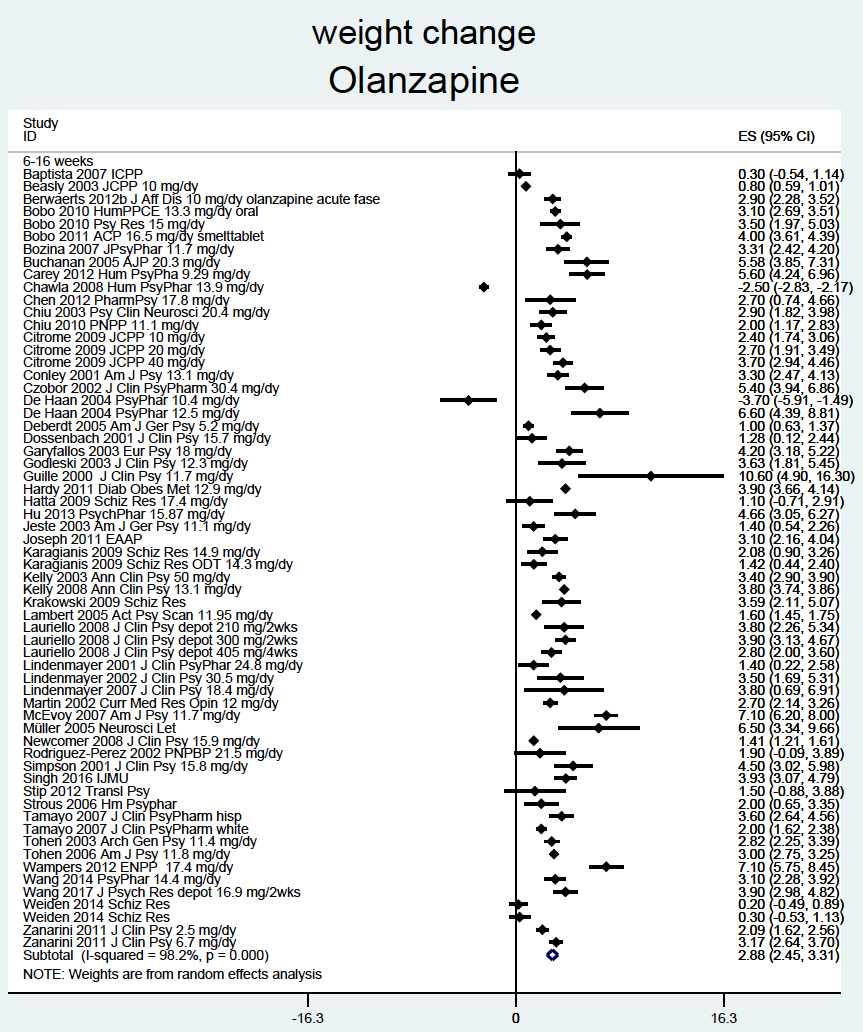


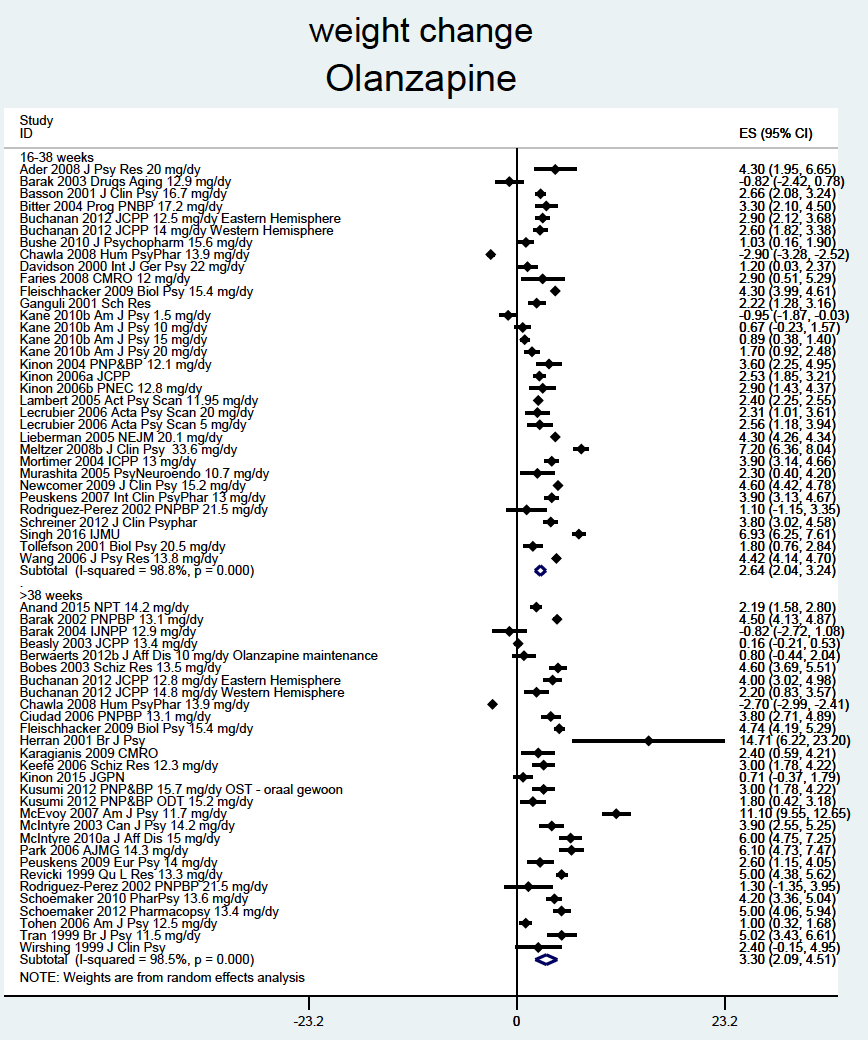


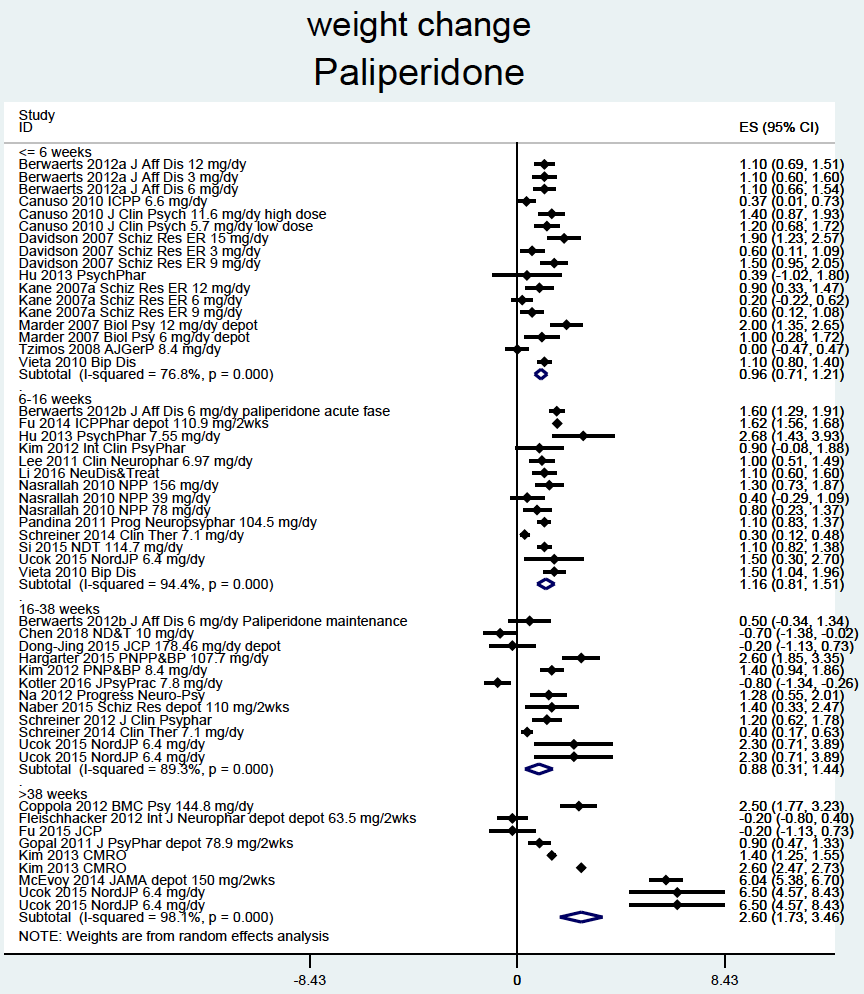


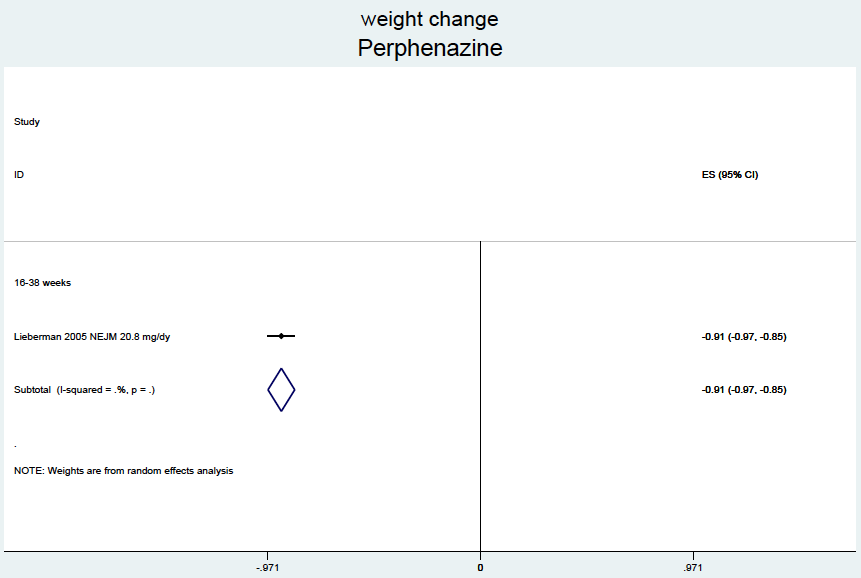


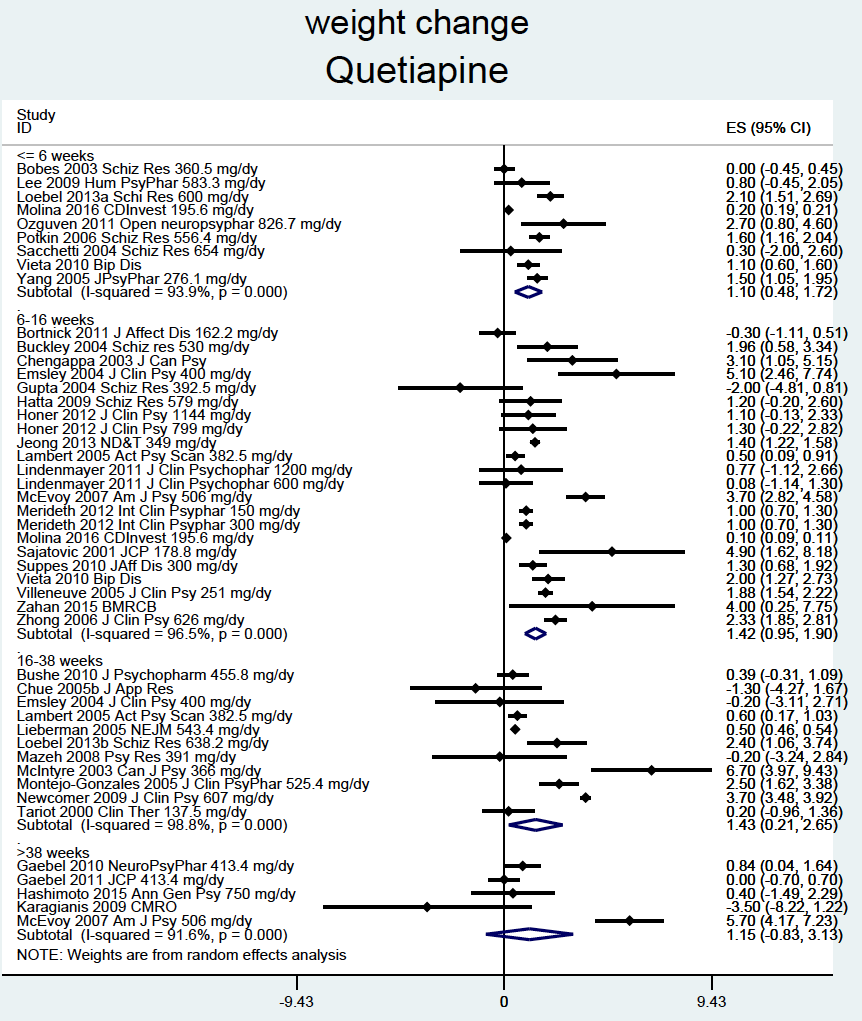


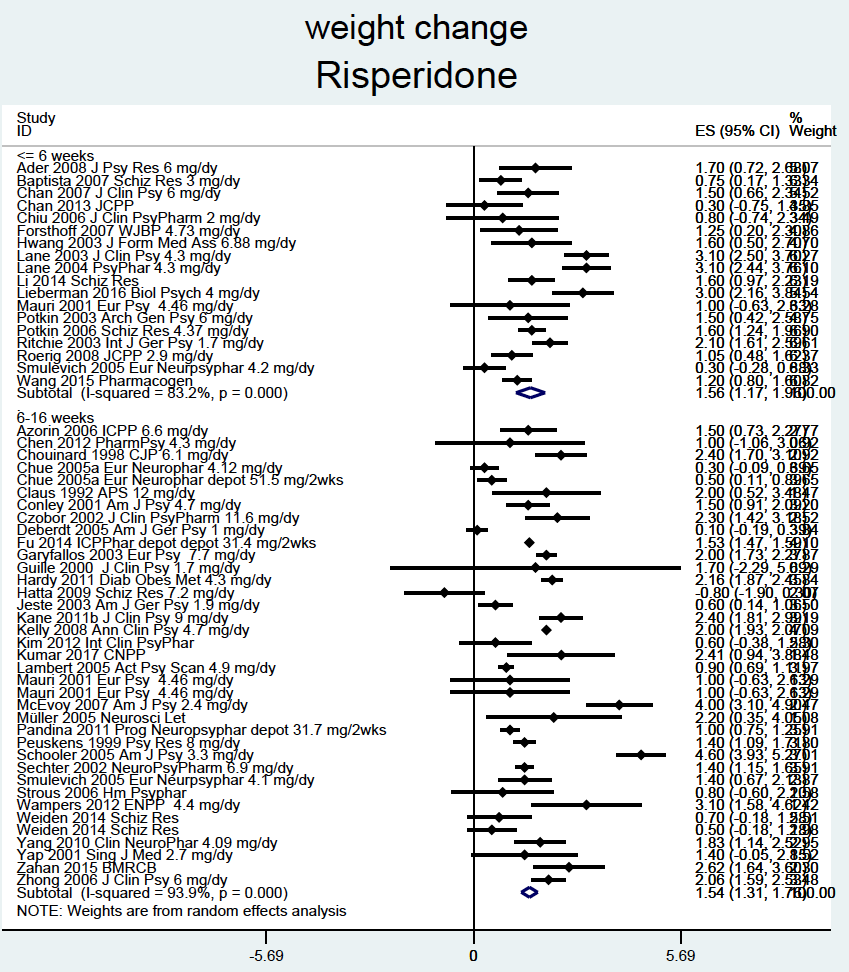


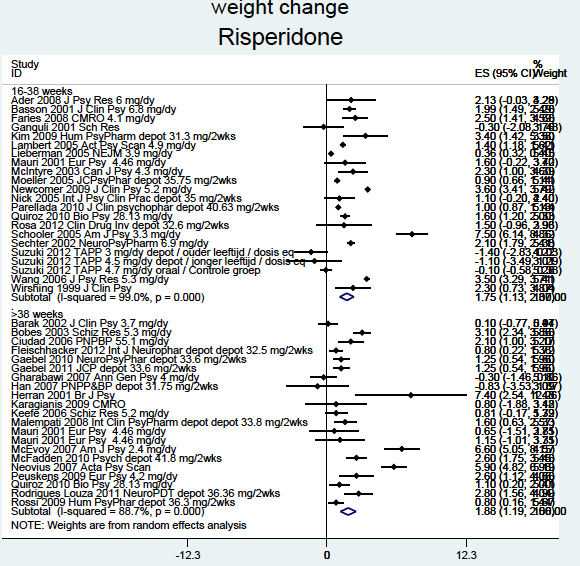


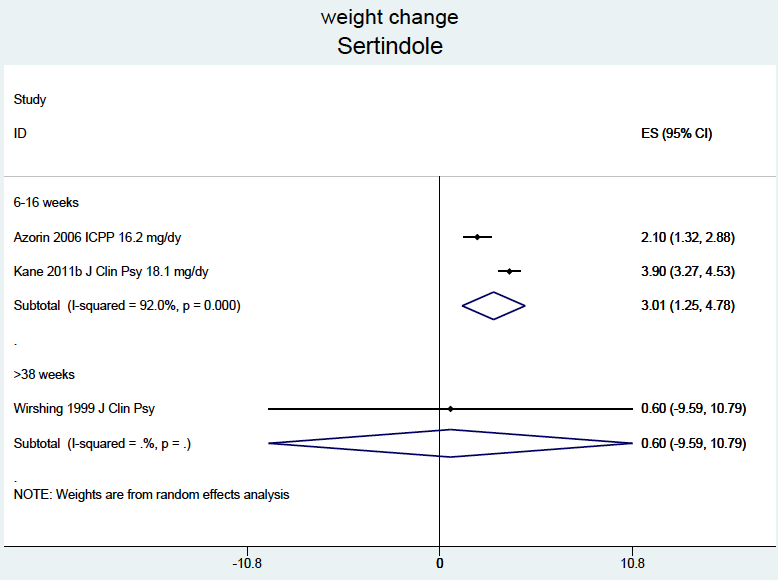


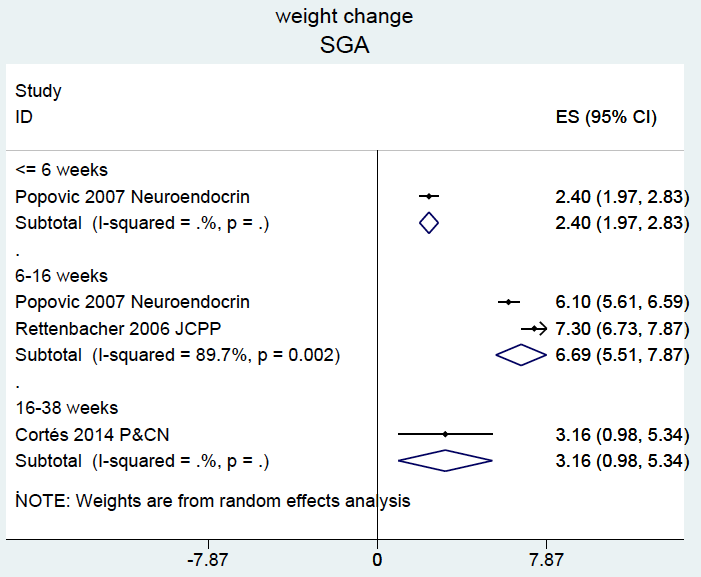


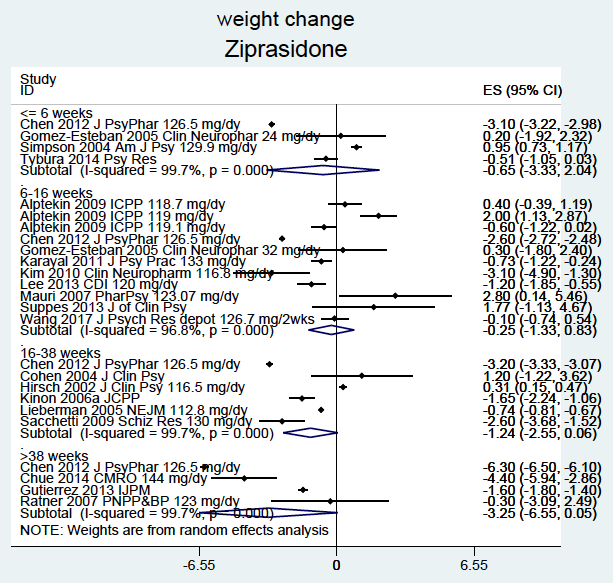


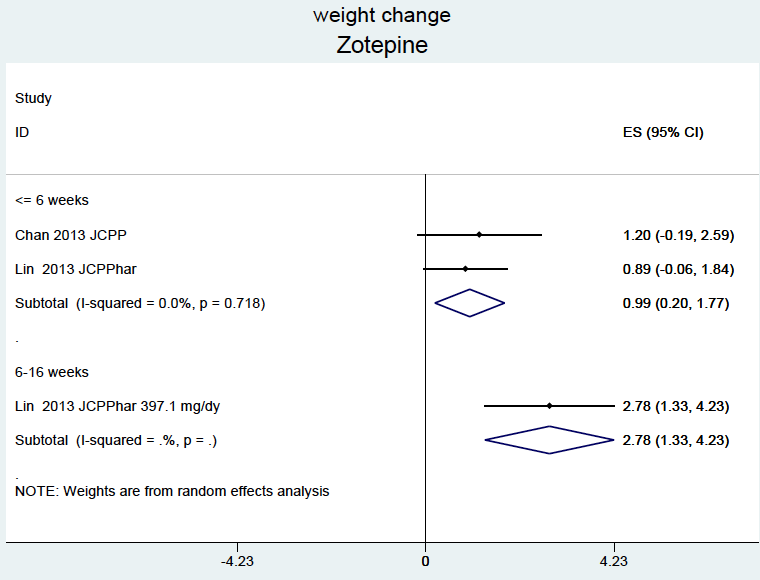


S5. All psychiatric diagnoses of the included papers and grouped diagnoses

| Study diagnosis | code | Group diagnosis |
| --- | --- | --- |
| Schizophrenia, , Schizoaffective disorder, Schizophrenia + Schizo-affective disorder, Schizophrenia + schizo-affective disorder + Schizophreniform, Schizophrenia + Schizo-affective disorder + Tardive dyskinesia, Schizophrenia + Schizophreniform | 1, 2,3,4,18, 19,20, 21, 23 | Schizophrenia |
| Schizophrenia + depressive disorder, Schizophrenia, Schizo-affective disorder + Bipolar + Depressive disorder, Schizophrenia + Schiz-affctive + cocaine dependency. | 24, 27, 41, | Psychosis spectrum |
| Bipolar disorder typ 1, Bipolar disorder type 1 + 2, Bipolar disorder type 1 + 2 + panic disorder + generalized anxiety disorder, Bipolar disorder type 1 + 2 + Depressive disorder | 5,6, 28,35 | Bipolar disorder |
| Cocaine dependency, Tourette’s Syndrome, Autism spectrum, Borderline personality, Trichotillomania, Behavioural disorders, Major depressive disorder, Pathological gambling, Healthy volunteers, Addiction, Dementia, Cognitive disorders, Amnestic disorders, Parkinson’s disease, OCD, PTSS | 8, 22, 25, 26, 29, 30, 33, 34,36, 40, 50, 31, 32,51, 52, 53 | Miscellaneous |

In this table all diagnoses that were mentioned in the various papers that were included in noted. We all coded these diagnosis in the data base and regrouped them in 4 diagnosis groups: schizophrenia, psychosis spectrum, bipolar disorders and miscellaneous.

S6

Regression coefficients of association between BMI-baseline (mean per study group) and weight gain, stratified by duration (Aripiprazole, Olanzapine, Risperidone, , placebo).

|  |  | <6 weeks | 6-16 weeks | 16-38 weeks | > 38 weeks |
| --- | --- | --- | --- | --- | --- |
| Aripiprazole | Switch | -0.14 (-1.07; 0.79) p=0.70 | 0.08 (-0.34; 0.51) p=0.66 | **-0.39 (-0.68; -0.10) p=0.014** | -0.14 (-0.31; 0.04) p=0.10 |
|  | Naive | ^1^ | -0.29 (-1.86; 1.28) 0.26 |  | 35 (-12; 82) 0.09 |
| Clozapine | Switch | -0.72 (-1.69; 0.25) p=0.09 | -0.49 (-2.51; 1.54) p=0.54 | -1.20 (-11; 8.8) p=0.37 | -1.9 (-4.6; 0.81) p=0.12 |
|  | Naive | ^1^ |  |  |  |
| Haloperidol | Switch |  | -0.26 (-0.68; 0.17) p=0.12 |  | **-1.54 (-2.5; -0.59) p=0.014** |
|  | Naive |  |  |  | 1.64 (-9.3; 12.6) p=0.30 |
| Olanzapine | Switch | 0.05 (-0.18; 0.28) p=0.67 | -0.15 (-0.44; 0.13) p=0.28 | **-0.44 (-0.89; -0.002) p=0.049** | **-0.80 (-1.53; -0.07) p=0.03** |
|  | Naive | 0.59 (-0.59; 1.78) p=0.29 | 0.56 (-0.81; 1.93) p=0.37 |  | -2.28 (-23; 18.5) p=0.68 |
| Risperidone | Switch | 0.06 (-0.27; 0.38) p=0.64 | -0.05 (-0.22; 0.11) p=0.50 | 0.39 (-0.07; 0.86) p=0.09 | -0.72 (-1.62; 0.19) p=0.11 |
|  | Naive | **0.94 (0.13; 1.75) p=0.035** | -4.1 (-239; 231) p=0.86 | -0.62 (-11; 10) p=0.59 |  |
| Ziprasidone | Switch |  | -0.12 (-0.38; 0.15) p=0.32 |  |  |
|  | Naive |  |  |  | 4.63 (-42; 51) p=0.43 |
| Placebo | Switch | 0.16 (-0.07; 0.39) p=0.15 | 0.25 (-0.09; 0.59) p=0.12 | -0.38 (-2.20; 1.43) p=0.55 | **0.63 (0.04; 1.21) p=0.04** |
|  | Naive | 0.01 (-0.35; 0.38) p=0.93 |  |  |  |

^1^ no data (insufficient observations)

S7

Funnel plots for six AP and placebo funnel plots were construed to check for publication bias

|  | < 6 weeks | 6-16 weeks | 16-38 weeks | > 38 weeks |
| --- | --- | --- | --- | --- |
|  | Aripiprazole |  |  |  |
| Switch |  | 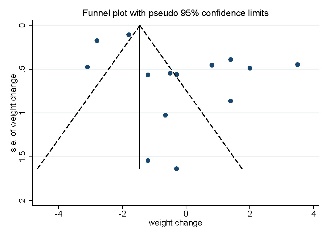 | 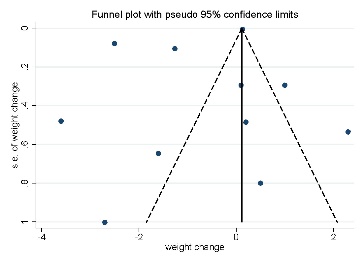 | 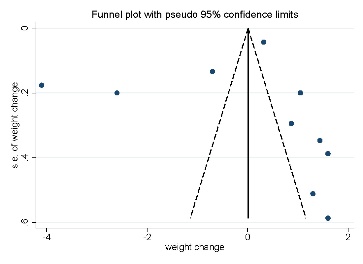 |
| Naive | 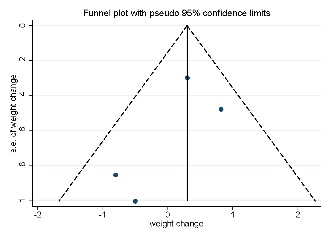 | 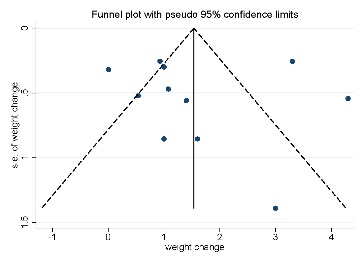 | 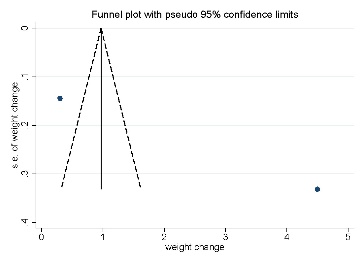 |  |
|  | Clozapine |  |  |  |
| Switch | 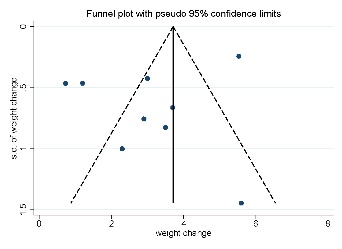 | 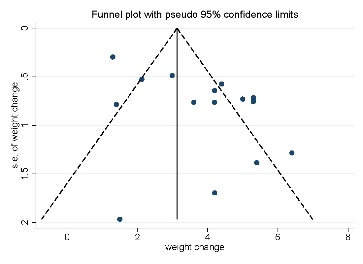 | 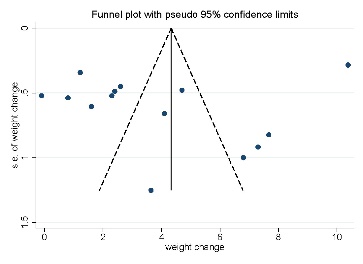 |  |
| Naive |  |  |  |  |
|  | Haloperidol |  |  |  |
| Switch | 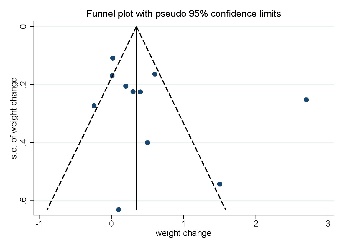 | 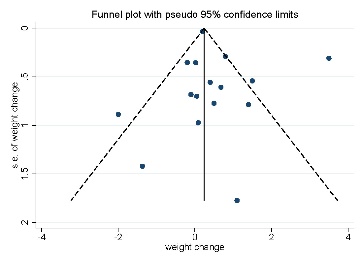 | 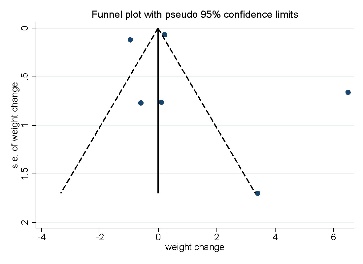 | 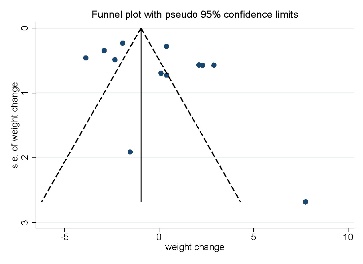 |
| Naive | 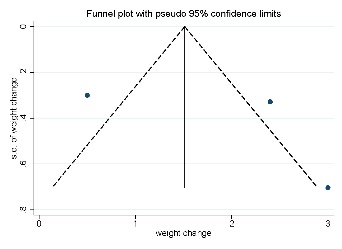 | 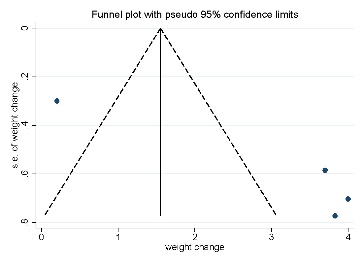 | 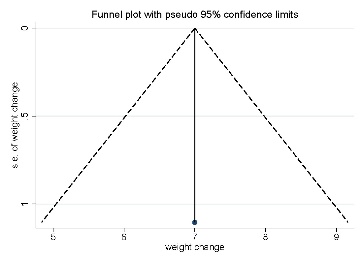 | 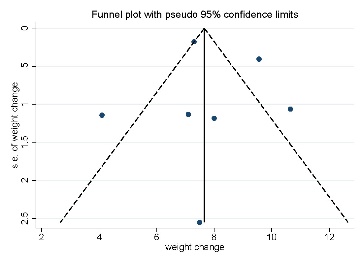 |
|  | Olanzapine |  |  |  |
| Switch | 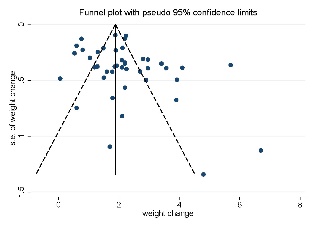 | 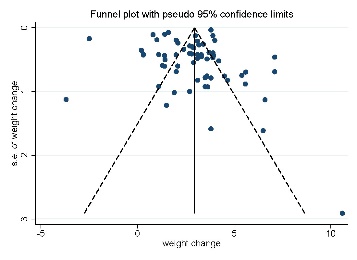 | 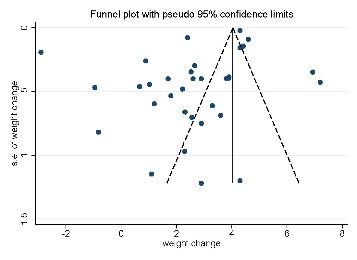 | 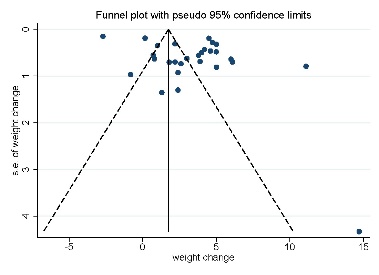 |
| Naïve | 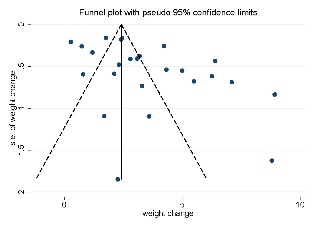 | 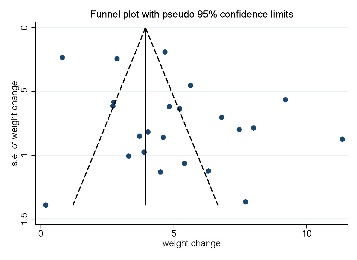 | 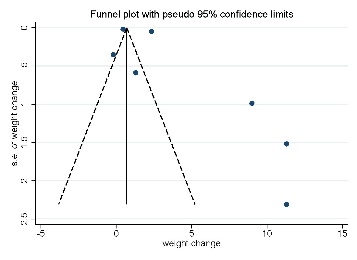 | 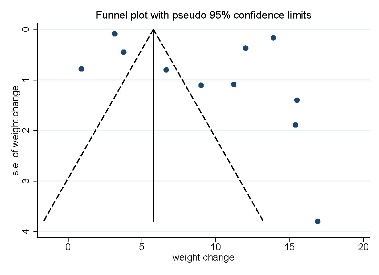 |
|  | Risperidone |  |  |  |
| Switch | 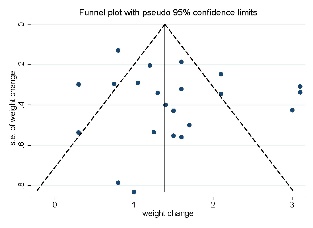 | 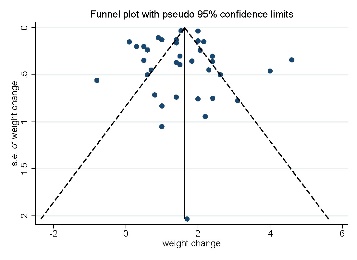 | 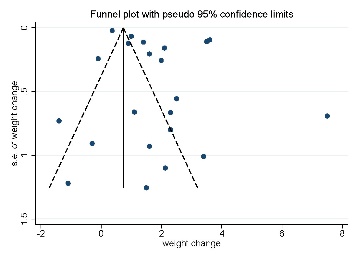 | 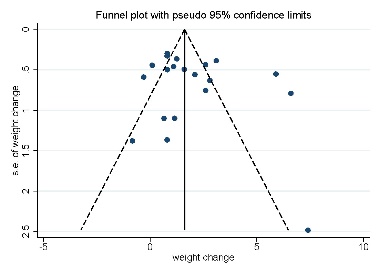 |
| Naïve | 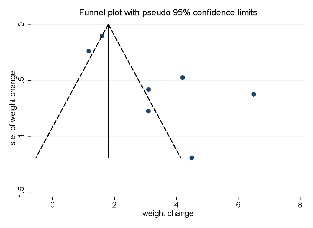 | 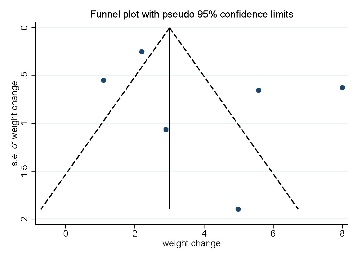 | 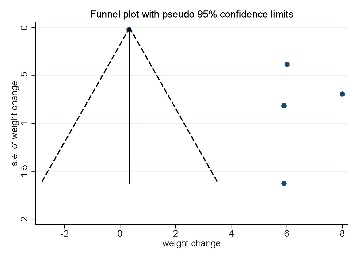 | 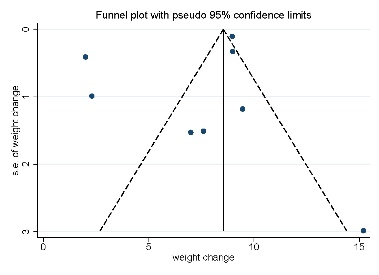 |
|  | Ziprasidone |  |  |  |
| Switch | 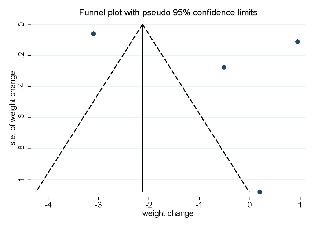 | 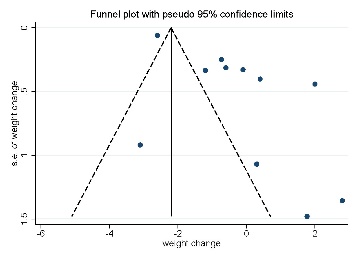 | 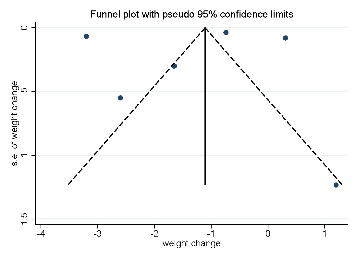 | 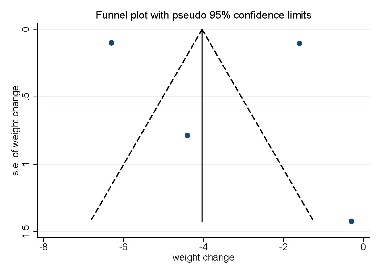 |
| Naïve | 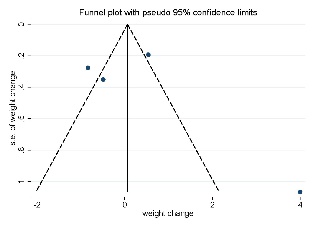 | 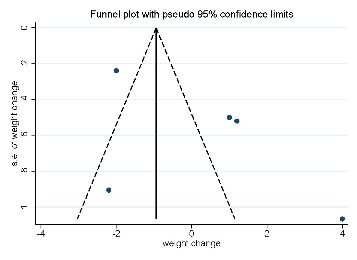 | 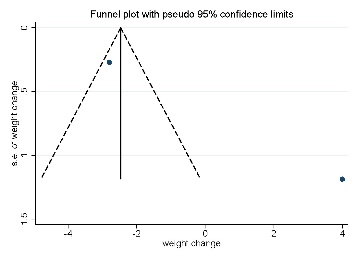 | 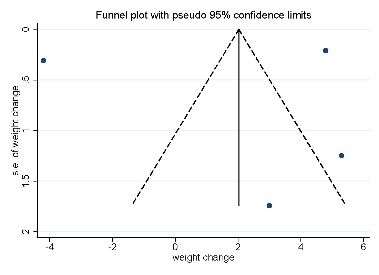 |
|  | Placebo |  |  |  |
| Switch | 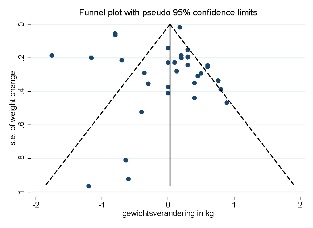 | 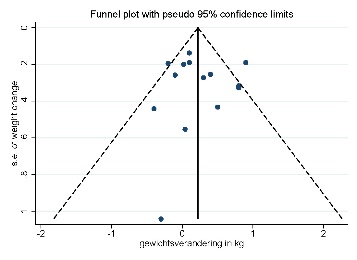 | 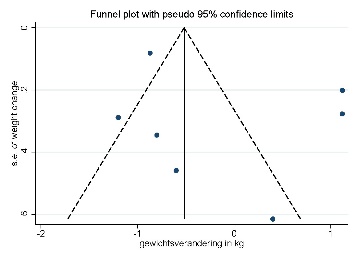 | 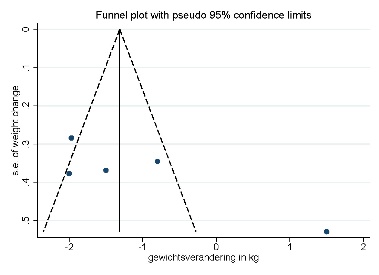 |
| Naive | 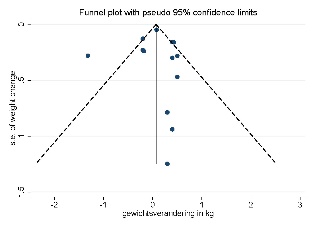 | 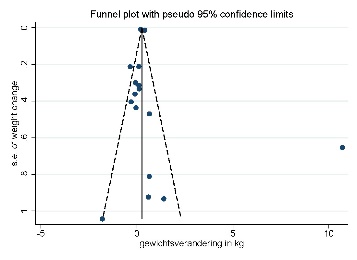 | 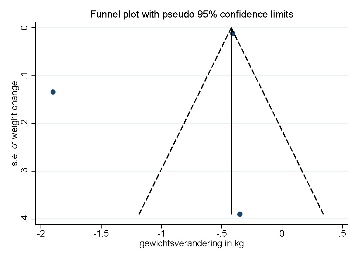 | 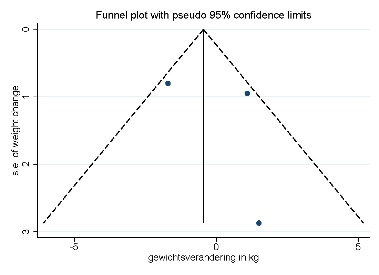 |

S8 Egger tests coefficient and p-value and added studies by trim-and-fill procedu

|  | Duration | Egger test switch group | t&f^1^ | Egger test in naive group | t&f |
| --- | --- | --- | --- | --- | --- |
| Aripiprazole | 1-6 weeks |  |  |  |  |
|  | 6-16 weeks |  |  |  |  |
|  | 16-38 weeks |  |  |  |  |
|  | >38 weeks |  |  |  |  |
| Clozapine | 1-6 weeks |  |  |  |  |
|  | 6-16 weeks |  |  |  |  |
|  | 16-38 weeks |  |  |  |  |
|  | >38 weeks |  |  |  |  |
| Haloperidol | 1-6 weeks |  |  |  |  |
|  | 6-16 weeks |  |  |  |  |
|  | 16-38 weeks |  |  |  |  |
|  | >38 weeks |  |  |  |  |
| Olanzapine | 1-6 weeks | 1.65 p=0.076 | 0 | 4.28 p=0.010 | 0 |
|  | 6-16 weeks | -1.53 p=0.19 | 15 | 3.31 p=0.052 | 0 |
|  | 16-38 weeks | -4.41 p=0.012 | 0 | 7.9 p=0.31 | 1 |
|  | >38 weeks | 6.15 p=0.015 | 0 | 7.37 p=0.35 | 0 |
| Risperidone | 1-6 weeks | 1.48 p=0.24 | 0 | 4.24 p=0.051 | 0 |
|  | 6-16 weeks | -0.72 p=0.39 | 0 | 3.58 p=0.36 | 0 |
|  | 16-38 weeks | 5.18 p=0.039 | 0 | 9.26 p=0.039 | 1 |
|  | >38 weeks | 1.81 p=0.27 | 0 | -2.29 p=0.43 | 2 |
| Ziprasidone | 1-6 weeks |  |  |  |  |
|  | 6-16 weeks |  |  |  |  |
|  | 16-38 weeks |  |  |  |  |
|  | >38 weeks |  |  |  |  |
| Placebo | 1-6 weeks |  |  |  |  |
|  | 6-16 weeks |  |  |  |  |
|  | 16-38 weeks |  |  |  |  |
|  | >38 weeks |  |  |  |  |

1 t&f: added studies using trim and fill

S9

Results of AP with only 1 study for that period.

AP-naive

| Antipsychotic | Time (wk) | N studies | n | Kg | 95%CI | I^2^ | Tau^2^ | Significance test Z | p |
| --- | --- | --- | --- | --- | --- | --- | --- | --- | --- |
| cariprazine | >38wk | 1 | 93 |  |  |  |  |  |  |
| chlorpromazine | >38 | 1 | 169 | 1.91 | 1.20 – 2.62 |  | 0.000 | 5.31 | 0.000 |
| clozapine | <6 | 1 | 14 | 6.52 | 4.73 – 8.31 |  | 0.000 | 7.15 | 0.000 |
| clozapine | 6-38 | 1 | 34 | 2.3 | 0.15 – 4.45 |  | 0.000 | 2.10 | 0.036 |
| FGA | 16-38wk | 1 | 21 | 7.00 | 4.34 – 9.16 |  | 0.000 | 6.34 | 0.000 |
| flupenthixol | >38 | 1 | 107 |  |  |  |  |  |  |
| haloperidol | 16-38 | 1 | 21 | 7.00 | 4.84 – 9.16 |  | 0.000 | 6.34 | 0.000 |
| iloperidone | <6 | 1 | 7 | 0.80 | 0.28 – 1.32 |  |  | 3.02 | 0.002 |
| paliperidone | <6 | 1 | 28 | 1.69 | 0.62 – 2.76 |  |  | 3.09 | 0.002 |
| paliperidone | 16-38 | 1 | 63 | -0.30 | -0.87 – 0.27 |  |  | 1.03 | 0.303 |
| paliperidone | >38 | 1 | 63 | 0.20 | -0.49 – 0.89 |  |  | 0.57 | 0.568 |
| perphenazine | >38 | 1 | 13 | 1.50 | -0.02 – 3.02 |  | 0.000 | 1.93 | 0.053 |
| SGA | >38 | 1 | 108 | 11.50 | 9.37 – 13.63 |  | 0.000 | 10.58 | 0.000 |
| sulpride | >38 | 1 | 162 | 1.86 | 0.98 – 2.74 |  | 4.13 | 0.000 |  |

Time is in weeks. N= number of studies. n=number of patient included in the study.

AP-switch

| amisulpride | >38 | 1 | 46 | -7.80 | -9.74 – -5.86 |  | 0.000 | 7.90 | 0.000 |
| --- | --- | --- | --- | --- | --- | --- | --- | --- | --- |
| blonanserine | <6 | 1 | 25 | 1.77 | 0.67 – 2.87 | --- | 0.000 | 3.15 | 0.002 |
| chlorpromazine | 6-16 | 1 | 21 | 2.27 | 2.04 – 2.50 | --- | 0.000 | 19.26 | 0.000 |
| chlorpromazine | 16-38 | 1 | 19 | 4.90 | 2.04 – 7.76 | --- | 0.000 | 3.36 | 0.001 |
| clopenthixol | 6-16 | 1 | 20 |  |  |  |  |  |  |
| FGA | >38 | 1 | 122 | 5.20 | 3.59 – 6.82 |  | 0.000 | 6.31 | 0.000 |
| iloperidone | 16-38 | 1 | 31 | 0.87 | 0.31 – 1.43 |  | 0.000 | 3.02 | 0.003 |
| levopromazine | 16-38 | 1 | 19 |  |  |  |  |  |  |
| melperone | <6 | 1 | 34 | 3.30 | -4.44 – 11.04 |  | 0.000 | 0.84 | 0.403 |
| melperone | 6-16 | 1 | 34 | 3.20 | -1.41 – 7.81 |  | 0.000 | 1.36 | 0.173 |
| perphenazine | 16-38 | 1 | 60 | -0.91 | -0.97 – 0.85 | --- | 0.000 | 29.18 | 0.000 |
| SGA | <6 | 1 | 13 | 2.40 | 1.97 – 2.84 | --- | 0.000 | 10.82 | 0.000 |
| SGA | 16-38 | 1 | 25 | 3.16 | 0.98 – 5.34 |  | 0.000 | 2.84 | 0.005 |
| sertindole | >38 | 1 | 8 | 0.60 | -9.59 – 10.79 | --- | 0.000 | 0.12 | 0.908 |
| zotepine | 6-16 | 1 | 35 | 2.78 | 1.33 – 4.23 | --- | 0.000 | 3.75 | 0.000 |

Time is in weeks. N= number of studies. n=number of patient included in the study.
