# Supplementary material for: Antipsychotics result in more weight gain in antipsychotic naive patients than in patients after antipsychotic switch and weight gain is irrespective of psychiatric diagnosis: A meta-analysis
Source: PLoS One. 2021 Feb 17;16(2):e0244944. doi: 10.1371/journal.pone.0244944 (PMC7888647; doi:10.1371/journal.pone.0244944)
Supplement: Attachment — Submitted filename: Reaction and answers of the comments _4r.docx [file pone.0244944.s004.docx]

Reaction and answers of the comments on: **Antipsychotics results in more weight gain in antipsychotic naive patients compared to antipsychotic switch and weight gain is irrespective of psychiatric diagnosis.**

PONE-D-20-24708

General points

**3. Thank you for stating the following financial disclosure:**

**"The funders had no role in study design, data collection and analysis, decision to publish, or preparation of the manuscript."**

**At this time, please address the following queries:**

a. **Please clarify the sources of funding (financial or material support) for your study. List the grants or organizations that supported your study, including funding received from your institution.**
b. **State what role the funders took in the study. If the funders had no role in your study, please state: “The funders had no role in study design, data collection and analysis, decision to publish, or preparation of the manuscript.”**
c. **If any authors received a salary from any of your funders, please state which authors and which funders.**
d. **If you did not receive any funding for this study, please state: “The authors received no specific funding for this work.”**

Dear editor,

Unfortunately, I apparently did not fill in the right answer. None of the authors received any funding for the is study or receives funding that is conflicting with this study. I really do apologize for this misunderstanding.

**4. Please include your tables as part of your main manuscript and remove the individual files. Please note that supplementary tables (should remain/ be uploaded) as separate "supporting information" files.**

Dear editor

We added the table into the manuscript and removed the individual files. I do apologize for misunderstanding the author guidelines here.

**Reviewer #1**

Thank you for your thorough comments and queries. It is appreciated.

**1 The reviewer states that: “The authors take a longer period of finding studies (from 1960 onwards) and claim they look at longer follow up periods, but they did the latter also in their previous meta-analysis.”**

Indeed, we extended the search from 1-1-1960 onwards till 30-06-2019. In our previous study the inclusion of papers started from 01-01-1999. At the time we felt we could build on the important paper by Allison 1999. Keeping in mind comments of the reviewers and comments made on that paper we decided to extend the search period from 01-01-1960 onwards. Unfortunately, most papers did not meet current methodological standards and had to be excluded. By extending our search to June 2019, we were able to include more studies. This resulted in more AP in the results and a more extensive analyses in the most used AP, i.e. trim-and-fill and other analyses assessing publication bias (also see comment #7).

**2 The reviewer argues that it is strange to compare two groups the so called AP naive and the AP-switch patient.**

We fully agree with the reviewer that the AP-switch group consist of a very heterogenous groups of patients. As the reviewer states reasons for switching antipsychotics can be multiple: changing an antipsychotic because of lack of effect, because of weight gain or other side effects, because of the research protocol patients participate in, and other reasons. Because the interpretation of the AP-switch group is difficult, we argue that results in the AP-naive group are far more informative considering the influence of antipsychotic on weight gain.

Thus, the fact that the AP-switch group is heterogenous underlines the importance to analyse naive patients and switch patients, separately. To prove that both groups really differ we feel the comparison is important to show that this a true assumption. It sheds another light on studies that do not make a clear distinction between AP-naive and AP switch. The recent studies by Huhn 2019 and Pillinger 2019, but also others before did not do this, explicitly. Thus, in the revised version we emphasize the reason for analysing naive and switch, separately, and we formulated a research question whether results in both groups differed as expected.

Future original studies could identify subgroups within the switch-patients to analyse them separately, but this is not possible in a meta-analysis when not available in the original studies.

Therefore we adapted the title (lines 1-3)

**Antipsychotics results in more weight gain in antipsychotic naive patients *than in patients after* antipsychotic switch and weight gain is irrespective of psychiatric diagnosis: *a meta-analysis***

In the abstract we adapted the text accordingly:

Line 56: “The present meta-analysis aimed to analyse *the impact of weight change of antipsychotics* in antipsychotic-naïve patients and antipsychotics switch patients and whether body weight change depended on diagnosis.”

Line 66-69:*“* *In the antipsychotic naive group, all antipsychotics resulted in body weight gain. In the antipsychotic switch group, most antipsychotics likewise resulted in bodyweight gain, with exception of amisulpride, aripiprazole and ziprasidone that showed no body weight gain or even some weight loss after switching antipsychotics.”*

Line 69-71	“Antipsychotic use resulted in *substantial* increase in body weight in antipsychotic-naive patients. In antipsychotic-switch patients the weight gain was mild to almost absent for amisulpride aripiprazole and ziprasidone.”

We think the comparison is still informative. AP-naives react unbiased and with more severe with weight gain than AP-switch patients. We corrected this in the text, putting more emphasis on the pluriform background of the studies part of the AP-switch groups. We meet the comments of the reviewer adapting the manuscript at the lines 114-122:

“*Furthermore*, the majority of the included *randomised controlled trial* (RCTs) *included in the meta-analyses have been* AP-switch studies*, so far*. *Findings from AP-switch patients are different from findings in AP-naive patients because previous AP-treatment was also associated with weight gain* (19)”

In the discussion we added the following:

*Lines 549 - 575: Nevertheless, the comparison between AP-naive and AP-switch should also capture also the notion that the AP-switch group is essentially a heterogenous group as the reasons for switching antipsychotics are pluriform. These reasons for switching APs may include: changing an antipsychotic because of lack of effect, because of weight gain or other side effects, because of the research protocol patients participate in, and various others. It should be expected that patients who gained a significant amount of body weight as a result of an AP treatment (e.g. olanzapine) may react with loss of body weight after switching to a more metabolically neutral AP. Despite switch groups are heterogeneous, the comparison between AP-naive and AP-switch shows that the differences in results between those two types of patients are important. Therefore it is necessary to analyse these groups of patients separately.*

**3 The reviewer argues that no network is performed, while recent network meta-analyses have been published already.**

Currently, we are performing a network meta-analysis together with an expert statistician. Research question of the network meta-analysis is whether there are significant differences in weight gain between AP. The present paper presents results of other research questions that are as important.

A network analysis using our data needs to take into account the multilevel structure in the data; i.e. some rows in the database are more similar to each other than other rows (e.g. a study has assessments at multiple time points and includes multiple AP). Thus, the assumption of independence is not met. The software package (metafor in R) needed to be revised to analyse this correctly. Previously published meta-analyses (Huhn 2019 and Pillinger 2019) (see refs 17, 18) did not analyse a multilevel structure. Both Huhn and Pillinger did not take into account duration and presented one result per study group only. Furthermore, Huhn included short-term studies only. This is why our analysis needs more time. Only after the analyses are finished, we can start writing results and discussion. For this reason, we studied another research question first.

Other remarks

**4 I don't know whether I agree that most meta-analyses are on switch patients; some of the more recent meta-analyses that I can find are of patients with acute psychotic symptoms.**

We agree with the reviewer that results of studies in AP-naive patients are more interesting (also see comment #2 reviewer 1). Indeed, recent studies and meta-analyses included acute psychotic symptoms (e.g. Huhn 2019 & Pillinger 2019). However, in those papers “patients with acute psychotic symptoms” were not always true AP-naive. Patients with acute psychotic symptoms is a heterogenous groups of patients, ranging from first episode patients who never had antipsychotic medication to patients who had a relapse of symptoms in ongoing history of a psychotic disorder and already used antipsychotic medication. Therefore, in the present paper we analysed AP-naive patients separately. AP-naive patients are patients who never had an AP before. In addition, we also analysed switch-patients, because that does give insight too and far more papers using switch patients are published (see comment #2, reviewer 1).

We thank the reviewer for addressing this issue. Apparently the distinction between AP-naive and AP-switch was not described clear enough. We added this in the method section.

Lines 126 – 131:

*Patients who received antipsychotic medication for the first time and had no history of antipsychotic use before, are defined as AP-naive patients. Patients who ever used an antipsychotic before the study started and were randomised into an antipsychotic are called AP-switch patients. This is a heterogenous group as the reasons for switching may vary: changing an antipsychotic because of lack of effect, because of weight gain or other side effects, because of the research protocol patients participate in, and other reasons.*

**5 The search in PubMed and EMBASE only seems limited. There are so many more literature data bases (e.g. PsychInfo, Cochrane, CiNAHL).**

We performed an extra search in Psychinfo, Cochrane, CiNAHL, Web of Science.

We did not find any new papers that met the inclusion criteria. So, no new papers were retrieved and no new data we added in the data set

**6 Furthermore, authors state they fear for publication bias as null results are less likely to be published, but have they checked the trial registries such as ClinicalTrials.gov, to search for trials that have been carried out but are not published? That would increase the reliability of the results.**

When trying to include unpublished studies, the authors of a systematic review should contact the authors to obtain the results. We contacted authors of published studies when information was missing, they hardly replied. Even when the RCT is registered, authors might not provide the results. So, the search for unpublished studies may not lead to sufficient data. In addition, there are also unregistered trials that are not published. Fortunately, statistical programmes have commands to explore publication bias and correct for it. Because we could include more studies, we did perform those commands and, fortunately, results remained similar (also see comment #7, reviewer 1).

**7 Some of the important information on e.g. publication bias, is presented only in the discussion and not in results. Also other posthoc analyses i.e.** **the role of BMI at baseline, are newly presented in the discussion while I think these belong earlier in the paper.**

We agree with the reviewer that analysis of publication bias deserves a more prominent place in the paper. We added a paragraph on publication bias before presenting the results in naive and switch patients (see lines 400 – 409)

*Publication bias*

*Funnel plots and Egger tests showed some evidence for publication bias in some of the analyses (supplementary data S7 and S8). In AP-switch patients, trim-and fill added studies, only in Olanzapine 6-16 weeks. In AP-naive patients trim and fill added studies in 16-36 weeks (Olanzapine) and >38 weeks (Olanzapine and Risperidone). In the rare case that trim-and-fill added studies, pooled estimate and p-value were rather similar to the original analysis (see also supporting information S7 and S8). Despite only the most frequently included AP were presented in a funnel plot, funnel plots with low numbers of studies could not be interpreted. Despite only the most frequently included AP were presented in a funnel plot, funnel plots with low numbers of studies could not be interpreted.*

In addition, we added two research questions to the main document (differences in weight gain between naive and switch; BMI at baseline). See lines 324-329

Lines 157 - 160:

*Additionally, the psychiatric diagnosis remains the same during the study period and therefore does not affect changes in body weight in RCT’s. Patients who gain body weight as a result of an antipsychotic may switch to another antipsychotic to stop weight gain or induce weight loss. In general, patients with a higher initial BMI may easier lose weight (29).*

Lines 180 – 183: *In addition, two more research questions were studied. (3) The difference in AIWG between AP naive and AP switch patients was analysed to evidence the separate analyses in both patients groups. (4) The difference in AIWG depending on average BMI at baseline was analysed.*

In the Result paragraph was added

Lines 530 - 534: *BMI at baseline as a modifier*

*“In AP switch patients some, but not all strata of Aripiprazole, Clozapine, Haloperidol and Olanzapine showed that a higher initial BMI was associated with less weight gain (see supporting information S6). In AP-naive patients and in patients randomised to Risperidone or placebo, this was not the case (see supporting information S6).”*

**8 On page 5, it is asserted that from the neuropharmacological view neurotransmitter induced interactions are independent of psychiatric diagnoses. This standpoint deserves some explaining.**

We appreciate this remark and might reflect some spurious communication in our paper and in the international literature. We hesitated to discuss this topic to extensive.

In itself an antipsychotic medication has several neurochemical interactions, that all have various and specific consequences for deregulation of appetite and other metabolic aspects, like influences on hypothalamus appetite regulation hormones NPY, AgRP, CART system etc, hormones like leptine and insuline and inflammatory. See for a nice overview Singh 2019 Eur J Pharmacology.

We adapted the paragraph in the paper accordingly:

Lines 143-158 Several neurotransmitter systems are responsible for the increased appetite, decreased satiation, and enlarged meals (24). *Antipsychotics display an array of interactions (through agonism or antagonism) of multiple neurotransmitters systems. Modes of action includes alterations in hypothalamic neuropeptides, striatal and mesolimbic dopamine reward pathways, gut hormones, peptides pituitary hormones, elevated proinflammatory cytokines, altered energy homeostasis and gut-dysbiosis.* (25). Histamine 3, 5HT2c and 5HT1b, M1 and M3 are linked to increased food intake through increasing appetite (26, 27). D2 antagonists, on the other hand, dampen the reward system urging the patient to eat more to experience some reward by food (28). From a neuropharmacological view, neurotransmitters induced interactions *of changes in appetite and metabolic disturbances* must be irrespective of a psychiatric diagnosis. Consequences of the neurotransmitters induced interactions should therefore lead to the same effects in healthy volunteers (20). The question is whether these more theoretical and laboratorial derived knowledge on weight gain by antipsychotics *can be* translated to clinical practice in daily life. *Additionally, the psychiatric diagnosis remains the same during the study period and therefore does not affect changes in body weight in RCT’s.*

In addition, in the discussion section we explain our results and we revised this paragraph in order to meet the reviewer6

In answering this comment we rewrote the paragraph as follows:

Lines 537 – 542:

*The present meta-analysis did not provide evidence that diagnosis is a moderator. This means that the AP related body weight changes are irrespective of the diagnosis. This seems counterintuitive. But the research literature is inconclusive. Some studies conclude that patients with schizophrenia appear more at risk for metabolic syndrome and diabetes mellitus compared with patient with bipolar disorder, (42). Whereas others reported no evidence for differences in body weight change depending on diagnosis (43-45).*

**9 On page 6 (and 7 and 8), authors contradict themselves in stating that they include RCTs only but also look at cohort studies, nonrandomized and other types of non-controlled studies. It should be made clear what types of studies authors include. (On page 9 it is mentioned again that authors looked for randomized studies.)**

We thank the reviewer for noticing this.

The meta-analysis data set only includes RCTs. However, follow-ups of the RCTs were also included to be able to analyse long-term changes in weight.

We agree with reviewer that we were not precise enough here and we revised the text.

Lines 188 – 189: A systematic review and meta-analysis of RCTs, *controlled clinical trials, and randomised open label studies* was performed …….

Lines 227 – 232:

*The inclusion criteria were:*

1. *Assessment of body weight change (continuous).*
2. *Age 18 years or older.*
3. *The data of weight change were available per AP.*
4. *RCT, controlled clinical trial or clinical trial or phase IV clinical trial with adequate control group with an intention to treat (ITT), open label studies with data per AP.*
5. *Publication date: 01-01-1960 / 30-06-2019.*

**10 On page 12, authors write: "For the second aim, meta-regression analyses were performed to assess whether diagnosis was a modifier." This could be described in more detail as not everyone will be acquainted with meta-regression analysis.**

In order to meet the reviewer, we rewrote the text. Also see comment #8, reviewer 1.

Lines 365 – 374: “*The variables diagnosis and naive/switch were added to the analyses to assess whether the association between AP and weight gain was different in different categories of the variables (moderation).”*

*For the second and third aim, meta-regression analyses were performed. Finally, in 6 most frequently used AP and in the placebo groups, a metar-egression was performed with BMI at baseline as a modifier. These 6 AP and placebo also had sufficient data to assess publication bias stratified by naive/swich and duration as in the original analyses (with the exception of Clozapine in naive patients). Funnel plots were obtained and Egger tests were performed (Stata commands meta-funnel and met-abias, respectively). Trim-and-fill analysis was performed to estimate effects of publication bias.*

**11 On page 14, figure 3, it is mentioned that number of patients is also in the figure (just like in figure 2) but this is not the case. Moreover, for amilsupride, the number of studies, for which long term effects (>38 weeks) were analysed, is not mentioned.**

In order to meet the reviewer we added the number of patients to all figures. Earlier, we chose to present the numbers only in the tables to reduce the information in the figures. Adding the number of included patients might decrease the readability of the figure (see comment #5, reviewer 2).

**In figure 2, for haloperidol, the periods are not formulated correctly (twice <6 wks), the number of patients also seem to be missing. The reporting does not seem very concise.**

Thank you. It is appreciated that you noticed this. We apologise.

It is corrected.

**12 On page 16, first paragraph of the discussion, the last sentence is: "The main finding was that all APs were associated with mean weight gain over time, with the exception of ziprasidone and placebo." However, "placebo" is not an antipsychotic.**

The reviewer is right. Placebo is not an antipsychotic. We corrected the sentence as follows:

Lines 542: “*Placebo (a control compound) showed a small weight loss over time*.”

**13 Page 17, first paragraph, the last sentence is repetition, this same sentence is also the last sentence on page 16.**

This is corrected by deleting the repetition.

**14 Page 19, top paragraph, the first sentence starts with 'lifestyle aspects', and then the second sentence starts with "This contributes". It is unclear to what 'this' is referring to. More in general, this paragraph about lifestyle, weight gain, diet, interventions and implication is not very clear. What is the point the authors wish to make here?**

We adapted the text so it becomes more clear what would like to address.

We assume that other factors like lifestyle issues and diagnosis are constant in a RCT or controlled study. In real life influences of lifestyles issues might vary between diagnostic groups and the impact on body weight and metabolic disturbances. See also answer on comment 8.

The paragraph has been change into:

Lines 637 – 642:

*The present meta-analysis did not provide evidence that diagnosis is a moderator. This means that the AP related body weight changes are irrespective of the diagnosis. This seems counterintuitive. Bu the research literature is inconclusive. Some studies conclude that patients with schizophrenia appear more at risk for metabolic syndrome and diabetes mellitus compared with patient with bipolar disorder, (42). Whereas others reported no evidence for differences in body weight change depending on diagnosis (43-45)*

**15 Page 20, line 479 and further, the authors stress the importance of weight loss interventions and subsequent health gains. Indeed, efficacy studies show weight loss is possible in AP users, but studies carried out in practice show weight loss is very hard to achieve when patients are already overweight or obese. I would suggest the authors stress weight gain prevention by closely monitoring weight when starting/switching AP, supporting patients to eat healthy and remain/become active. Losing (a lot of excess) weight is much more difficult than not gaining it.**

We fully agree and appreciate that monitoring and prevention is a first approach. We adapted the text. The sentence “Therefore, attention for weight gain as result of antipsychotic treatment, is key for any treatment plan” is changed into (lines 681 – 683): “*Monitoring and body weight management at the beginning of treatment with an AP are important. Ultimately, prevention of weight gain is many times preferable than painful efforts to lose body weight.* ”

**16 Page 21, line 513, authors mention they are confident Chinese studies are methodologically sound, while other meta-analyses excluded Chinese studies for methodological reasons. Could the authors explain why they are confident about methodological soundness while others are not?**

This is a good point and should disserve international debate. We are familiar that certain most esteemed scientist and researcher exclude Chinese papers and research beforehand. We also understand and acknowledge that Chinese research may show methodological flaws. However, we only included English written papers in peer-reviewed journals we may have some confidence in the peer-review process of these international operating journals. Second, we checked every paper according scientific and methodological strength and shortcomings according the NOS. Lastly, we believe that open not discriminatory mind is important. Otherwise good important papers might have been excluded unnecessarily.

We could check whether this group of studies has different results than the other studies (metaregression with Chinese/non-Chinese as a modifier), but this was not feasible before the deadline. We will study this topic in an separate paper.

We added the following:

Lines 743 – 746: Chinese studies are excluded because of a higher risk of methodological shortcomings (17). In the present meta-analysis, we included studies from China that were *published* in English as we were *able to evaluate and confirm* that the *studies* were methodologically sound. *The included research from China that were conducted in China authored by Chinese authors solely were all published in peer-reviewed journals, thereby ensuring a level of scientific validity and reliability*.

**17 Page 22, line 526, authors are worried about studies with null-findings not being studied. But did authors check trial registries?**

This comment is addressed already (see answer to comment 6)

**18 Page 22, line 542, the authors state "The AP-naive AIWG was noticeable for all antipsychotics in short and longer terms, triggered by APs induced increase of appetite." This latter part, the weight gain being triggered by APs induced increase of appetite, was not topic of this study and should not be mentioned in the conclusion. This is not what this study found (nor is it found in other literature; surely the increased appetite plays a role but this is not the only cause of AIWG).**

We appreciate this remark of the reviewer and agree that it is not the focus of this study.

We deleted this part of the sentence

Minor remarks

**19 I have read several times: "increased bodyweight gain" but I do not think this is right. Shouldn't in be either increased body weight, or body weight gain.**

The reviewer is right that this is ambiguous language. Throughout the text we corrected this in either “*increased body weight*” “i*ncrease in body weight*” or “*body weight gain*”

**20 Page 5, line 118, the word 'that' does not seem right in this sentence.**

We deleted the word “that”

**21 Page 6, line 138/139, this sentence is not correctly formulated.**

We reformulate the sentence as follows:

Lines 192 – 192: *“To ensure that MOOSE and PRISMA criteria were met*, we followed a protocol intended for internal use and not published.”

**22 Page 10, line 231/232, this sentence does not read well, it seems some words are missing.**

The reviewer is right. There is some text present that should have been deleted therefore causing ambiguity. We corrected this as follows:

Lines 309 – 311: “*Weight change was calculated by subtracting end of study body weight from post-baseline study body weight (body weight baseline - end body weight).* “

**23 Page 13, line 303, increase in weight gain?**

We corrected this in: ……. *an increase in body weight since initation*…….. See also comment reviewer 2. It is corrected thought the manuscript

**24 Page 15, line 361, indicating AP switch do not differ compared.... This sentence is difficult to understand (does not seem correct)**

We deleted the whole sentence as it dos contribute anything the discussion

**25 Page 18, line 448, result should be results.**

We corrected accordingly

**26 Page 20, line 490, underestimation is one word.**

We corrected accordingly

**27 Page 21, line 512, are should be were.**

We corrected accordingly

**28 Page 22, line 530/531, this sentence is not complete and not easy to understand.**

Egger tests showed that there was publication bias, but not in all analyses (16 in total: 2 AP, 4 durations, naive and switch) trim and fill added extra studies (see supplement S6).

We moved all text on publication bias to the methods and results section of the paper (see comment #7, reviewer 1) and we rewrote it.

**29 Page 23, line 551/552, references are not correct**

Strange that references were not converted by Endnote. This corrected.

**30 In the tables, often , is used rather than . for the decimals.**

This is corrected

------------

Reviewer 2

Thank for your encouraging comment. It is appreciated.

**1 The reviewer argues is ambiguous and stilted in some places of the manuscript.**

We checked made adaptations where appropriate. We also asked an experienced English speaker and writer to correct the manuscript accordingly.

**2 Figure legends are usually presented as a separate file, not internally in the text, and then the final print version put each figure legend below the figure.**

The reviewer is right. We adapted this in the revised manuscript

**3 You say you selected APs where there was at least information for 2 studies, (LINE 257), but tables 1 and 2 show many entries for an antipsychotic at a specific longest length of treatment where there was only one study, so I think you have to clarify this, or you expressed it incorrectly in the English.**

We aimed to include only AP where data are available from at least 2 studies. Because we include four time periods, it is possible that both studies have data of different time periods. In this case, results from 1 time period can be from 1 study only. This is in fact not a real meta-analysis, but we chose to include as much as possible.

In addition, in the previous version among others amoxapine and zuclopenthixol in switch and chlorpromazine and flupentixol in naive only included 1 study in total. This was not our intention and we removed these AP from the present results.

All outcome in which only 1 study was available have been removed from the tables and figures.

**4 Both in the tables and figures or legends to the tables and figures you mix up N and n. You seem to say N is for number for studies and n is for number of subjects, but then in legends to the tables or the bottom of the figures you have only N and N, so things are not clear.**

By accident we did not use the correct annotations. N = number of studies and n = number of patients included. This has been corrected in the figures legends and tables.

**5 It is very hard to read the base of figure 3. It is very hard to make out the words and numbers. In Figure 2 things are larger and so can be read although there is still confusion between number of studies and number of subjects because both are in capital N. Can you put the bottom of table 3 in bold, or make the font bigger, so things can be read?**

We agree with reviewer that the X-axis in the figure 3 is small. Of course Figure 2 is much easier to read as less AP are presented. We tried several options to improve readability, but this is the best we could do.

We left results based on only 1 study out of the tables and figures. These result are now available in supporting information S9

**6 Please specify in the legends to tables 1 and 2 how the Es was calculated. Is this mean difference, standardized mean difference or what?**

We revised the legends of tables 1 and 2. We chose to present body weight gain rather than standardised mean differences (SMD) because body weight is uniformly assessed in all studies so converting to SMD was not necessary. In addition, weight gain is far easier to interpret than SMD.

See tables 1 & 2, lines 427 - 431 and 420 – 474 & 970 – 971 and 989 - 990

**7 The results and discussion state that the supposed more weight friendly APs—such as amisulpride, aripiprazole and ziprasidone, were not associated with significant overall weight loss in switch studies. It would help clarify if these switch studies included switches from many different antipsychotics, or only or mostly from olanzapine or clozapine. If one were to restrict the switch studies to switches from olanzapine or clozapine, are there enough studies to make a conclusion, and would these studies show a weight loss when switching to one of these three alternative antipsychotics.**

Why aripiprazole, amisulpride and ziprasidone show no weight gain after switch is a very important topic. As commented by reviewer #1 the switch group is heterogeneous and there are various reasons why patients switch antipsychotic medication. One reason would be increase of body weight and the idea that switching to a metabolic friendly antipsychotic may help in reducing weight.

Analyses stratified by AP before the switch or by reason for the switch would be very interesting. However, if this is not assessed in the original studies, we cannot include it in our meta-analyses.

We added the following lines 533 – 537:

*BMI at baseline as a modifier*

In AP switch patients some, but not all strata of Aripiprazole, Clozapine, Haloperidol and Olanzapine showed that a higher initial BMI was associated with less weight gain S7). In AP-naive patients and in patients randomised to Risperidone or placebo, this was not the case (see supporting information S7).

In the discussion we added line 582 - 590:

*We hypothesized that patients with higher BMI at baseline would gain less weight during the study period. This means that patients with higher BMI including those who gained a significant amount body weight by a previous AP might show weight decrease. Whereas lower BMI may increase the risk for more substantial body weight gain. This might explain more weight gain in the AP-naive as they are thought to be younger and more lean. In this meta-analysis this cannot be tested. The analyses are just explorative, because BMI at the subject level was not available. Future original studies should be performed to replicate this finding. This is in line with the notion that the younger and more lean patients are at risk the most* (37-39).

**8 Maybe the authors have to caution about switching in terms of balancing efficacy and wright gain and side effects. Metaanalyses has generally shown clozapine to be the most efficacious antipsychotic especially for treatment resistant patients, and a few studies show olanzapine may be effective for some treatment resistant patients. In some metaanalyses olanzapine is ranked 2 or 3rd as most efficacious, although some of the differences are not large. So maybe there should be a cautionary comment about weight gain adverse effects vs efficacy, especially since some studies with olanzapine and clozapine show that clinical response was correlated with weight gain.**

We appreciate the comment of the reviewer. He is right, many meta-analyses showed already the beneficial effects of clozapine and olanzapine. We corrected the text in the conclusion so that the advice is better balanced.

In the conclusion we added the following:

Lines 678 – 680: *”* *Our findings reiterate that the monitoring and management of bodyweight from the beginning of AP treatment is extremely important since w*eight gain is easy to measure and follow-up; and weight loss interventions offer direct improvement on *physical and mental functioning.”*

Lines 684 – 686:*” Monitoring and body weight management at the beginning of treatment with an AP are important. Ultimately, prevention of weight gain is many times preferable than painful efforts to lose body weight.*”

Lines 784 – 786: “*However, considering all effects of an AP before initiating or changing an AP is crucial. Therefore, in AP-naive patients take all effects and risk factors for increased liability for adverse effects like weight gain into consideration.*”

In this meta-analysis we cannot compare the AP. So, which AP result in less weight gain after start AP in AP-naive patients or which AP results in most weight loss after AP-switch remains inconclusive. See also answer to reviewer 1 comment 3. We plan a network analysis to answer this question, whether the various AP can be compared and do the AP really statistical significant differ.

The most effective AP clozapine and olanzapine appear also to associated with highest risk for body weight gain.

In the conclusion we added the following:

Lines: 780 – 781: *Troublesome is the most effective AP c*lozapine and olanzapine *also* show the most severe increase in body weight.
